# Prevalence and clonal diversity of carbapenem-resistant *Klebsiella pneumoniae* causing neonatal infections: A systematic review of 128 articles across 30 countries

**DOI:** 10.1371/journal.pmed.1004233

**Published:** 2023-06-20

**Authors:** Ya Hu, Yongqiang Yang, Yu Feng, Qingqing Fang, Chengcheng Wang, Feifei Zhao, Alan McNally, Zhiyong Zong

**Affiliations:** 1 Center of Infectious Diseases, West China Hospital, Sichuan University, Chengdu, China; 2 Division of Infectious Diseases, State Key Laboratory of Biotherapy, Chengdu, China; 3 Center for Pathogen Research, West China Hospital, Sichuan University, Chengdu, China; 4 Institute of Microbiology and Infection, College of Medical and Dental Sciences, University of Birmingham, Birmingham, United Kingdom

## Abstract

**Background:**

*Klebsiella pneumoniae* is the most common pathogen causing neonatal infections, leading to high mortality worldwide. Along with increasing antimicrobial use in neonates, carbapenem-resistant *K*. *pneumoniae* (CRKP) has emerged as a severe challenge for infection control and treatment. However, no comprehensive systematic review is available to describe the global epidemiology of neonatal CRKP infections. We therefore performed a systematic review of available data worldwide and combined a genome-based analysis to address the prevalence, clonal diversity, and carbapenem resistance genes of CRKP causing neonatal infections.

**Methods and findings:**

We performed a systematic review of studies reporting population-based neonatal infections caused by CRKP in combination with a genome-based analysis of all publicly available CRKP genomes with neonatal origins. We searched multiple databases (PubMed, Web of Science, Embase, Ovid MEDLINE, Cochrane, bioRxiv, and medRxiv) to identify studies that have reported data of neonatal CRKP infections up to June 30, 2022. We included studies addressing the prevalence of CRKP infections and colonization in neonates but excluded studies lacking the numbers of neonates, the geographical location, or independent data on *Klebsiella* or CRKP isolates. We used narrative synthesis for pooling data with JMP statistical software. We identified 8,558 articles and excluding those that did not meet inclusion criteria. We included 128 studies, none of which were preprints, comprising 127,583 neonates in 30 countries including 21 low- and middle-income countries (LMICs) for analysis. We found that bloodstream infection is the most common infection type in reported data. We estimated that the pooled global prevalence of CRKP infections in hospitalized neonates was 0.3% (95% confidence interval [CI], 0.2% to 0.3%). Based on 21 studies reporting patient outcomes, we found that the pooled mortality of neonatal CRKP infections was 22.9% (95% CI, 13.0% to 32.9%). A total of 535 neonatal CRKP genomes were identified from GenBank including Sequence Read Archive, of which 204 were not linked to any publications. We incorporated the 204 genomes with a literature review for understanding the species distribution, clonal diversity, and carbapenemase types. We identified 146 sequence types (STs) for neonatal CRKP strains and found that ST17, ST11, and ST15 were the 3 most common lineages. In particular, ST17 CRKP has been seen in neonates in 8 countries across 4 continents. The vast majority (75.3%) of the 1,592 neonatal CRKP strains available for analyzing carbapenemase have genes encoding metallo-β-lactamases and NDM (New Delhi metallo-β-lactamase) appeared to be the most common carbapenemase (64.3%). The main limitation of this study is the absence or scarcity of data from North America, South America, and Oceania.

**Conclusions:**

CRKP contributes to a considerable number of neonatal infections and leads to significant neonatal mortality. Neonatal CRKP strains are highly diverse, while ST17 is globally prevalent and merits early detection for treatment and prevention. The dominance of *bla*_NDM_ carbapenemase genes imposes challenges on therapeutic options in neonates and supports the continued inhibitor-related drug discovery.

## Abstract 2

In an updated systematic review, Zhiyong Zong and colleagues report the prevalence and clonal diversity of carbapenem-resistant Klebsiella pneumoniae responsible for neonatal infections across 30 different countries.

## Author summary

**Why was this study done?:**

- *Klebsiella pneumoniae* is a leading cause of neonatal infections with high mortality, representing a severe global problem.
- Carbapenem-resistant *K*. *pneumoniae* (CRKP) has emerged as a particularly severe challenge for infection control and treatment.
- Understanding the global burden of neonatal infections caused by CRKP and their clonal background could provide critical insights for guiding countermeasures, but no systematic review has been accomplished to address this gap.

**What did the researchers do and find?:**

- We systematically reviewed all published articles addressing CRKP in neonates and included a total of 128 studies comprising 127,583 neonates in 30 countries and 2,057 neonatal CRKP isolates.
- We estimated a 0.3% pooled prevalence of CRKP infections in hospitalized neonates with a 22.9% pooled mortality. We found that neonatal CRKP strains were diverse in clonal background and most lineages did not exhibit intercountry dissemination.
- We utilized publicly available CRKP genomes with neonatal origins in GenBank to complement the literature for data about species, strain types, and carbapenemase types.
- However, we observed intercontinental distribution of ST17, representing a particular widespread CRKP lineage associated with neonates, and most neonatal CRKP strains encode a metallo-β-lactamase, particularly NDM (New Delhi metallo-β-lactamase).

**What do these findings mean?:**

- CRKP is a severe threat to this vulnerable population worldwide, in particular for those in LMICs.
- Most CRKP lineages in neonates appeared to emerge locally.
- Individualized approaches that are informed by local data may be needed for designing countermeasures.
- The dominance of carbapenemase genes impose challenges to effective treatment of neonatal infection. Our findings support the need for ongoing research into effective therapeutics.

## Introduction

Neonatal mortality is a major concern for public health [1], especially in low- and middle-income countries (LMICs) where optimized use of antimicrobial agents is problematic for many neonates [2]. The Every Newborn Action Plan aims for countries to have ≤12 neonatal deaths per 1,000 livebirths by 2030 or ≤10 by 2035 [3]. However, neonatal mortality rates vary significantly across countries, with a substantial proportion of deaths occurring in LMICs. In 2020, an estimated 2.4 million neonates died worldwide at an average global rate of 17 deaths per 1,000 live births (https://data.unicef.org/topic/child-survival/neonatal-mortality/). More than half of neonatal deaths are associated with infections [4]. In particular, neonates in LMICs have infection rates 3 to 20 times higher than those in high-income countries [5].

*Klebsiella pneumoniae* is a leading pathogen for neonatal infections [6] and is actually a complex comprising 5 closely related species including *Klebsiella pneumoniae* (*sensu stricto*), *Klebsiella quasipneumoniae*, *Klebsiella variicola*, *Klebsiella quasivariicola*, and *Klebsiella africana* [7]. Phenotype-based identification kits or systems such as API 20E, Vitek II, and BD Phoenix are widely used in clinical microbiology laboratories but provide limited performance for differentiating *K*. *pneumoniae* complex at the species level [8,9]. Matrix-assisted laser desorption ionization-time of flight mass spectrometry (MALDI-TOF MS) is also widely used for species identification, but misidentification of *Klebsiella* species occurs [10]. Even 16S ribosomal RNA (a constituent of the small subunit of prokaryotic ribosomes) gene sequencing, a common method of the molecular genetic approaches for species identification, does not reliably identify species within the *K*. *pneumoniae* complex [11,12]. Precise species identification relies on whole-genome sequencing and phylogenetic analysis. As such, *K*. *pneumoniae* reported in many studies may actually represent the complex rather than *K*. *pneumoniae* (*sensu stricto*).

In LMICs, the incidence of neonatal *K*. *pneumoniae* infection varies between 4.1 and 6.3 per 1,000 livebirths with a case fatality rate of 18% to 68% [5]. *K*. *pneumoniae* is also responsible for 16% to 28% of neonatal blood culture–confirmed sepsis in different countries [5]. A systematic review has estimated that the pooled proportion of neonatal sepsis caused by gram-negative organisms was 60% and that *Klebsiella* species accounted for 38% of sepsis caused by gram-negatives [13]. Another systematic review has identified that among 84,534 neonates from 26 countries in sub-Saharan Africa, *Klebsiella* spp. accounted for 15% to 21% of culture-positive sepsis [14]. Although the 2 systematic reviews did not specify the species [13,14], it appears that most *Klebsiella* strains causing sepsis were *K*. *pneumoniae* by examining the included studies.

Among bacterial pathogens in neonates, *K*. *pneumoniae* is notorious for the high prevalence of resistance towards most antimicrobial agents [15]. In particular, carbapenem-resistant *K*. *pneumoniae* (CRKP) is associated with increased mortality of patients [16], represents a serious public health threat, and has become the dominant pathogen disseminated in healthcare settings [17]. However, surveillance in many LMICs is often problematic in completeness and fidelity largely due to insufficient local data, inconsistent laboratory quality, and scarce microbiological facilities [18]. To date, the global burden of neonatal infections caused by CRKP has not yet been assessed.

Accordingly, we performed a systematic review of studies reporting population-based neonatal infections caused by CRKP to evaluate the availability of data worldwide and then attempted to provide an estimated prevalence of CRKP in neonatal infection globally. In addition, we utilized publicly available CRKP genomes with neonatal origins to complement the literature for information about species, strain types, and carbapenemases.

## Methods

### Search strategy and selection criteria

This study was designed and is reported according to the Preferred Reporting Items for Systematic Reviews and Meta-Analysis (PRISMA; S1 Text) and registered in PROSPERO (ID: CRD42022346445). The full protocol for this study is available in the supporting information (S2 Text). We searched PubMed, Web of Science, Embase, Ovid MEDLINE, Cochrane, bioRxiv, and medRxiv to identify studies up to June 30, 2022, that reported neonatal infections caused by CRKP. The search strings were “*Klebsiella*” and “neonate,” “*Klebsiella*” and “neonatal,” “*Klebsiella*” and “newborn,” “*Klebsiella*” and “NICU,” “CRKP” and “neonate,” “CRKP” and “neonatal,” “CRKP” and “newborn,” and “CRKP” and “NICU.” We also examined articles in the reference lists of retrieved articles. We included all studies in English or non-English languages. For studies in a language other than English and Chinese, we used Google translate for translation.

We assessed studies addressing the prevalence of CRKP infections and colonization in neonates. We examined all study populations regardless of age, presence of symptoms, or study location. We considered surveillance, cohort, nested cohort, case-control, or cross-sectional, and randomized studies for estimating the prevalence. We also investigated case reports and microbiological and genomic studies for species, strain types, and carbapenemases of CRKP strains in neonates.

We excluded studies where the numbers of neonates in the study or the geographical location are not available, from estimating the prevalence. We also excluded studies lacking independent data on *Klebsiella* or CRKP isolates. For data that were from the same study population but were reported in multiple articles, we extracted those from the most comprehensive report. LMICs were defined using the list of World Bank (https://datahelpdesk.worldbank.org/knowledgebase/articles/906519-world-bank-country-and-lending-groups).

### Data extraction

Two groups (4 investigators, 2 per group) independently reviewed the identified studies to determine eligibility. Disagreement over inclusion was resolved by arbitration by an additional, fifth, investigator.

Using a standardized data abstraction sheet, the following variables were recorded from articles that met our inclusion criteria: study design, country, study setting, study participants, year, population, age, sex, CRKP numbers and/or proportions, source of infection, antimicrobial susceptibility profiles, genomes and data availability, sequence types (STs), antimicrobial resistance genes, and primary outcomes.

### Quality assessment

The quality of each included article (S1 Dataset) was assessed independently by 2 investigators using either the Newcastle–Ottawa Scale (NOS) quality assessment tool for case-control, cross-sectional, and cohort studies or Cochrane risk of bias tool for randomized controlled trials. A consensus was reached by a panel discussion among 5 investigators. The results of quality assessment are summarized in S2 Dataset for cross-sectional studies, S3 Dataset for cohort studies, and S4 Dataset for case-control studies.

### Statistical analysis

We extracted raw data from the included studies and recalculated estimates wherever possible based on the extracted data. Descriptive analyses were used to describe the population. Pooled prevalence, mortality, and colonization rate were calculated and were presented with 95% confidence intervals (CIs). Rates and proportions were compared across groups using the chi-squared test. Analyses were performed using JMP Pro 14 statistical software (JMP; Cary, NC, USA) and Review Manager (RevMan) version 5.4.1 (The Cochrane Collaboration, 2020). Considering the limited number of eligible studies and the heterogeneity of the studies, we employed narrative synthesis to estimate the pooled prevalence, mortality, and colonization rate without measuring the biases with RevMan using the “Generic Inverse Variance” type and “Random” mode. Continuous variables were presented as median and interquartile range (IQR). Categorical variables were presented as numbers and percentages. All *p*-values of <0.05 were considered statistically significant.

### Construction of dataset used for *de novo* genomic analysis

First, all BioProjects (*n* = 2,761) mentioning neonates or neonatal ICUs in their titles or descriptions were retrieved from NCBI by searching strategy “neonatal[All Fields] OR neonate[All Fields] OR neonates[All Fields] OR newborn[All Fields] OR newborns[All Fields] OR nicu[All Fields]” up to June 30, 2022. By applying filter “Bacteria” and manual curation for *K*. *pneumoniae* complex in neonates, 37 BioProjects with short reads in Sequence Read Archive (SRA) or assembly remained. Second, all BioSamples (*n* = 86,635) under the genus *Klebsiella* were retrieved, and those with the presence of keywords neonatal, neonate, newborn, or NICU (neonatal intensive care unit) were included for further curation. Third, genomes with accession numbers mentioned in all included publications (*n* = 21) regarding neonate-associated *K*. *pneumoniae* complex were also included in the dataset. Of note, local assemblies from SRA data were preferred over NCBI-retrieved ones. BioSample duplications, SRA with sequencing depth lower than 30 × and reads obtained from Nanopore sequencing only were discarded.

### Genomic profiling and analysis

All included genomes (*n* = 2,093), each with a unique BioSample number, were subjected to a series of quality control and genome profiling measures. All bioinformatic settings and thresholds were set to the default unless otherwise specified. Among the 2,093 genomes, 264 were assembled and 1,829 had only short reads without assembly available. Therefore, short reads of the 1,829 genomes were retrieved from SRA of NCBI and were trimmed to remove adapters and reads shorter than 30 bp using Trimmomatic v.0.39 [19]. For each of the 1,829 genomes, maximum coverage of 150 × sequencing depth was subsampled using SeqKit v2.2.0 [20] and assembled into a draft genome using SPAdes v3.15.3 [21] under the isolate mode.

The precise species identification for each genome was performed using FastANI v1.33 [22] by comparing genomes with type strain of each species from the genus *Klebsiella*. The completeness and heterogeneity of genomes were assessed using CheckM v1.1.10 [23]. STs and virulence factors were identified using Kleborate v2.2.0 [24]. Antimicrobial resistance genes were detected using AMRFinderPlus v3.10.23 [25]. Plasmid replicons were typed using PlasmidFinder v2.1 [26].

## Results

### There were 128 articles reporting neonatal CRKP infection or colonization in 30 countries of 4 continents

**Fig 1:**
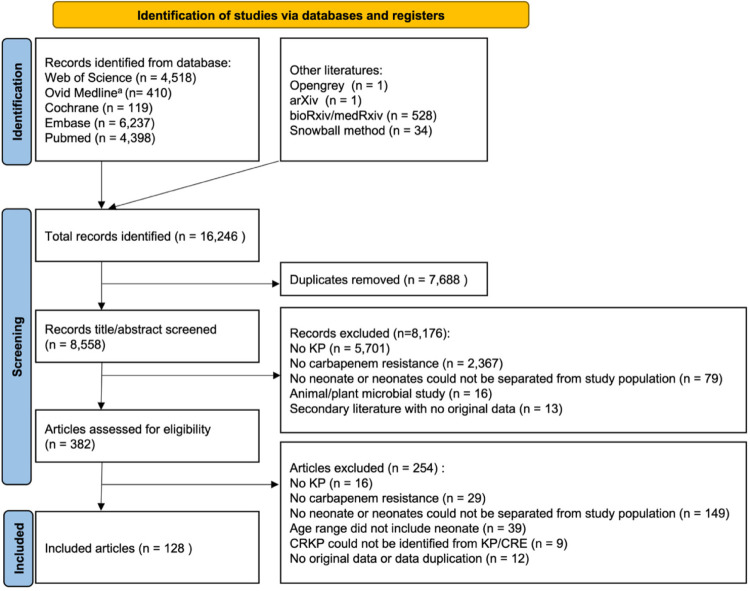
Flow chart of inclusion and exclusion of studies for the systematic reviews. ^a^For Ovid MEDLINE, we defined the limit at age group as “Newborn infant (birth to 1 month).” CRE, carbapenem-resistant *Enterobacterales*; CRKP, carbapenem-resistant *Klebsiella pneumoniae*; KP, *Klebsiella pneumoniae*.

**Fig 2:**
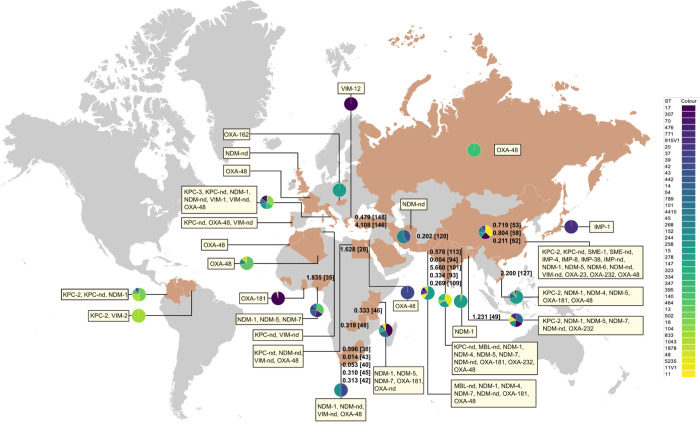
The worldwide distribution of CRKP in neonates. The base layer of the map was downloaded and adapted from the website https://mapsvg.com/maps/world under the CC BY 4.0 license (https://creativecommons.org/licenses/by/4.0/). Countries with the identification of CRKP in neonates are filled with brown. The CRKP infection prevalence in neonates are indicated by percentage (%) followed by the reference number. The pie charts illustrate the proportions of the 5 most common STs seen in the corresponding countries. Carbapenemase types and variants found in neonatal CRKP strains are indicated. CRKP, carbapenem-resistant *Klebsiella pneumoniae*; IMP, imipenemase; KPC, *Klebsiella pneumoniae* carbapenemase; MBL, metallo-β-lactamase; NDM, New Delhi metallo-β-lactamase; OXA, oxacillinase; SME, *Serratia marcescens* enzyme; ST, sequence type; VIM, Verona integron-encoded metallo-β-lactamase; -nd, not determined.

A total of 16,246 articles were identified from various literature sources (Fig 1). After removing duplicates, 8,558 articles were assessed for eligibility by screening the title and the abstract. There were 382 articles eligible for full-text review, among which 254 were excluded (Fig 1). Finally, 128 articles at the time of writing, none of which were preprints, were analyzed (S1 Dataset). The 128 articles comprised a total study population of 127,583 neonates in 30 countries of Africa (*n* = 10; Algeria [27], Egypt [28,29], Ghana [30], Guinea [31], Morocco [32,33], Nigeria [34–37], South Africa [38–45], Tanzania [46], Tunisia [47], and Zambia [48]), Asia (*n* = 10; Bangladesh [34,49], China [50–92], India [34,93–113], Iran [114], Israel [115], Japan [116], Nepal [117], Pakistan [34,118–122], Saudi Arabia [123,124], and Vietnam [125–128]), Europe (*n* = 8; France [129], Greece [130], Hungary [131], Italy [132–142], Portugal [143], Russia [144], Turkey [145–147], and the United Kingdom [148]), and South America (Colombia [149–152] and Venezuela [153]) (Fig 2). Among the 30 countries, 21 were LMICs.

Most (*n* = 112, 87.5%) of the 128 studies were retrospective, while 15 (11.7%) were prospective and one contained both retrospective and prospective data. The study design comprises cross-sectional (*n* = 87, 68.0%), case report or series (*n* = 31, 24.2%), case-control (*n* = 8, 6.3%), and cohort (*n* = 3, 2.3%). Only cross-sectional, cohort, and case-control studies were used to estimate the prevalence of neonatal CRKP infections. The overall quality of included studies varied significantly. For cross-sectional studies, the median of NOS scores was 3 (IQR, 2 to 4) with 3 to 5 seen in most studies (65%, 56/86). Lower scores were mainly present in the selection of CRKP-negative groups for comparability, assessment of the outcome, and appropriately statistical tests (S2 Dataset). The 3 cohort studies were all scored 4 with lacking description of non-CRKP cohort and follow-up for outcomes (S3 Dataset). Six of the 8 case-control studies received a score of 6, one scored 7, and the remaining one scored 8, most of which lacked comparability between CRKP-positive cases and controls (S4 Dataset).

Susceptibilities to 1 carbapenem (any of ertapenem, imipenem, and meropenem) or 2 to 4 carbapenems (any 2 or all of the aforementioned 3 with or without doripenem) were reported in 113 out of the 128 included studies to demonstrate carbapenem resistance of studied CRKP strains. Among the remaining studies, 5, 2, and 1 study used whole genome sequencing, modified Hodge test, and PCR to detect carbapenemase genes or the production of carbapenemases, respectively, while 7 did not specify the methodology to define carbapenem resistance (S5 Dataset).

### Bloodstream infection is the main type of neonatal CRKP infections

The 128 included studies documented 2,057 CRKP isolates from infected neonates or from colonized sites between 2005 and 2020. Of which, 1,504 isolates had clearly documented information of the sample type. Blood (*n* = 561, 37.3%) is the most common sample type of the 1,504 clinical CRKP isolates from infected neonates, followed by sputum (*n* = 262, 17.4%), and urine (*n* = 64, 4.3%) (S1 Table). The findings suggest that bloodstream infection, which often leads to sepsis, is likely the main type of neonatal CRKP infections.

### The pooled prevalence of CRKP infection was 0.3% for hospitalized neonates

Prevalence of neonatal CRKP infections was reported in 23 studies, all of which contained hospitalized neonates only. Unfortunately, we were unable to find a study addressing the prevalence of CRKP infections for nonhospitalized neonates. The 23 studies comprised a total of 123,842 hospitalized neonates in 11 countries, all of which are LMICs (Bangladesh, China, Egypt, India, Nigeria, Pakistan, South Africa, Tanzania, Turkey, Vietnam, and Zambia) (Table 1 and Fig 2). The median number of neonates in the 23 studies was 1,704 (IQR, 409 to 3,836; range 218 to 36,285). The pooled prevalence of CRKP infection in hospitalized neonates was 0.3% (95% CI, 0.2% to 0.3%; range 0.0% to 5.7%). After excluding the BAR-RDS network study, in which data of Asian and African countries could not be separated for analysis, the remaining 22 studies were included for comparison. The prevalence (0.7% [95% CI, 0.4% to 1.0%]) in Asian countries was significantly higher than that (0.3% [95% CI, 0.1% to 0.4%]) in African countries (*p* = 0.006). Among the 23 studies, 15 reported data from NICU. Prevalence of CRKP neonatal infection in NICU was significantly higher than that in non-ICU neonatal care units (0.7% [95% CI, 0.5% to 0.9%] versus 0.0% [95% CI, 0.0% to 0.0%]; *p* < 0.001).

**Table 1:** Prevalence of neonatal infections caused by CRKP^a^.

Country	Study		Ward	no., neonates	Neonatal CRKP infection		Reference
	start	end			no.	prevalence, %	
Bangladesh	Oct-2016	Jan-2017	neonatal	406	5	1.231	[49]
BAR-RDS network^b^	Nov-2015	Dec-2017	-	36,285	70	0.193	[34]
China	Aug-2012	Apr-2013	NICU	474	1	0.211	[92]
China	Jan-2017	Dec-2018	NICU	3,200	23	0.719	[53]
China	Jul-2017	Jun-2018	NICU	5,348	43	0.804	[58]
Egypt	Feb-2019	Sep-2019	NICU	860	14	1.628	[28]
India	Oct-2006	Sep-2008	NICU	1,383	8	0.578	[113]
India	Jan-2008	Dec-2012	-	22,363	1	0.004	[94]
India	Jan-2010	Feb-2012	-	318	18	5.660	[101]
India	Jan-2012	Dec-2012	NICU	1,794	6	0.334	[93]
India	Jan-2015	Dec-2018	NICU	3,347	9	0.269	[109]
Nigeria	May-2011	Dec-2011	SCBU	218	4	1.835	[35]
Pakistan	Jul-2017	Dec-2017	NICU	2,480	5	0.202	[120]
South Africa	Jan-2008	Nov-2008	NICU	503	3	0.596	[38]
South Africa	Jan-2009	Dec-2013	neonatal	28,284	4	0.014	[43]
South Africa	Jan-2012	Nov-2012	neonatal	1,903	1	0.053	[40]
South Africa	Jan-2015	Dec-2016	NICU	6,443	20	0.310	[45]
South Africa	Jan-2017	Aug-2018	neonatal	3,836	12	0.313	[42]
Tanzania	Mar-2009	Sep-2009	-	300	1	0.333	[46]
Turkey	Jan-2010	Dec-2014	PICU/NICU	1,671	8	0.479	[145]
Turkey	Jan-2017	Jul-2018	NICU	1,704	70	4.108	[146]
Vietnam	Oct-2012	Sep-2019	NICU	409	9	2.200	[127]
Zambia	Oct-2013	May-2014	NICU	313	1	0.319	[48]

^a^-, not available; CRKP, carbapenem-resistant *K*. *pneumoniae*; ICU, intensive care unit; NICU, neonatal intensive care unit; PICU, pediatric intensive care unit; SCBU, special care baby unit.

^b^BAR-RDS network, Burden of Antibiotic Resistance in Neonates from Developing Societies. The BAR-RDS network consists of 12 clinical sites in 7 countries in Africa (Ethiopia, Nigeria, Rwanda, South Africa) and South Asia (Bangladesh, India, Pakistan), among which Ethiopia and Rwanda did not report CRKP.

Patient outcomes of neonatal CRKP infections were reported in 21 studies comprising 302 CRKP neonatal infections and 79 neonatal deaths (S2 Table). The pooled mortality of infected neonates was 22.9% (95% CI, 13.0% to 32.9%), ranging from 0% to 100%.

Eleven studies comprising 9,938 neonates also had data about the prevalence of CRKP colonization (S3 Table). Six studies reported the colonization rate of CRKP in neonates via screening of rectal swabs but did not specify the timing of screening [27,140,143,145,147,152]. The pooled CRKP colonization of these studies was 6.2% (95% CI 4.0% to 8.4%; range 0.1% to 31.0%). One additional study based on rectal swabs of neonates (*n* = 326) in Vietnam specified the colonization rates at admission and at discharge, of 1.8% (6/326) and 3.7% (12/326), respectively [126]. The study exhibited an increasing colonization rate following admission, indicating the nosocomial acquisition of CRKP.

### Most neonatal CRKP strains were *K*. *pneumoniae* (*sensu stricto*)

In the 128 included studies, 103 specified the methods used for species identification (S1 Fig). Most studies (*n* = 83) relied on phenotype-based methods and systems for species identification, while 34 studies employed whole genome sequencing allowing precise identification. However, among the 34 studies, only 9 specified the methods for species identification with 3 using the well-recognized average nucleotide identity (ANI) (S4 Table). The 9 studies comprised 191 neonatal CRKP strains with 178 (93.2%) belonging to *K*. *pneumoniae* (*sensu stricto*) and the remaining 13 (6.8%) to *K*. *quasipneumoniae* (S4 Table).

To complement the understanding of species, STs, and carbapenem genes, we also included all genomes of neonatal CRKP available in public databases. A total of 2,093 genomes of *K*. *pneumoniae* complex, each with a unique BioSample number, from neonates were identified. After quality control, 101 genomes were discarded from further analysis for completeness less than 95% (*n* = 37), contamination greater than 5% (*n* = 31), heterogeneity greater than 30% (*n* = 27), or genomes derived from species other than *K*. *pneumoniae* complex (*n* = 6). Therefore, 1,992 genomes of *K*. *pneumoniae* complex were included for analysis. Carbapenemase-encoding genes were identified in 535 (26.9%) genomes (S6 Dataset), of which 331 could be linked to publications (21 studies) and 204 were newly identified here, providing complementary data for strain distribution and carbapenem resistance of neonatal CRKP.

Among the 535 neonatal CRKP strains, 498 (93.1%) belonged to *K*. *pneumoniae* (*sensu stricto*), 33 (6.2%) to *K*. *quasipneumoniae* and 4 (0.7%) to *K*. *variicola*. By combing the results in literature and the genome-based analysis, it becomes evident that most neonatal CRKP strains were indeed *K*. *pneumoniae* (*sensu stricto*) but could also belong to other species within the complex.

### Neonatal CRKP strains exhibited diverse clonal backgrounds with sequence type 17 (ST17), a globally disseminated lineage

Identifying epidemic lineages of CRKP seen in neonates is crucial for informing both treatment and prevention. We collected data on the ST of neonatal CRKP strains from all included studies, among which 69 reported data about STs of 1,145 CRKP strains. We additionally included the 204 neonatal CRKP genomes not linked to any publications. We therefore analyzed a total of 1,349 strains, which were recovered from 20 countries (Africa: Algeria, Ghana, Kenya, Nigeria, and South Africa; Asia: Bangladesh, China, India, Iran, Israel, Japan, Nepal, Pakistan, and Vietnam; Europe: Greece, Hungary, Italy, and Russia; South America: Colombia and Venezuela) (S5 Table). The 1,349 strains could be assigned to a total of 146 STs (S5 Table for all ST and Table 2 for those seen in at least 2 countries), indicating the high diversity of neonatal CRKP. Of note, among the 146 STs, 8 were newly identified here and are unnamed at present (S6 Table). ST15 (*n* = 183), ST17 (*n* = 136), and ST11 (*n* = 123) were the 3 most common types of neonatal CRKP. In particular, neonatal CRKP of ST17 was found in 8 countries across 4 continents (Africa, Asia, Europe, and South America), suggesting that it is a widespread lineage associated with neonatal infections. ST11 was found in 8 countries of 3 continents (Africa, Asia, and Europe). Other STs seen in 3 continents were ST54 (*n* = 45) and ST101 (*n* = 11), but these 2 STs were only found in 3 countries, i.e., 1 country per continent. ST15 (*n* = 183) was present in 6 countries of Asia and Europe, while ST307 (*n* = 42) were seen in 4 countries of Asia and Europe. ST14 (*n* = 34) and ST147 (*n* = 25) were both found in 4 countries of 1 continent. The remaining 138 STs were found in 1 to 3 countries of 1 or 2 continents.

**Table 2:** ST of neonatal CRKP seen in at least 2 countries^a^.

ST	No., strains^b^	Country		Continent		Reference
		List^c^	No.	List^d^	No.	
17	136 (117+19)	Bangladesh, China, Colombia, Ghana, Greece, Italy, Kenya, Vietnam	8	A, E, F, S	4	[30,34,51,53,55,81,82,89,126,128,139,149]
11	123 (108+15)	Bangladesh, China, India, Italy, Nepal, Nigeria, Pakistan, Vietnam	8	A, E, F	3	[34,50,51,58,59,62,64,65,72,75,79,126,128,139]
54	45 (38+7)	China, Italy, Kenya	3	A, E, F	3	[55,70,74,81,88,139]
101	11	China, Colombia, Italy	3	A, E, S	3	[84,133,139,150]
15	183 (160+23)	China, Hungary, India, Nepal, Pakistan, Vietnam	6	A, E	2	[34,57,60,104,106,117,122,126,128,131]
307	42 (39+3)	Bangladesh, China, Italy, Vietnam	4	A, E	2	[64,71,72,75,125,135,137–139]
20	48 (40+8)	China, Nigeria, Pakistan	3	A, F	2	[34,51,55,74,79,81,88]
39	31 (30+1)	Israel, Nigeria, South Africa	3	A, F	2	[39,115]
395	14	Italy, Russia, Nigeria	3	E, F	2	[34,138,139,144]
48	10 (3+7)	Ghana, India, Pakistan	3	A, F	2	[30,106]
35	4 (2+2)	Bangladesh, China, Italy	3	A, E	2	[59,139]
231	3 (2+1)	Bangladesh, India, Italy	3	A, E	2	[106,139]
45	17	Algeria, China	2	A, F	2	[27,51,53,75]
1043	7	Colombia, Vietnam	2	A, S	2	[126,151]
1412	6	China, Italy	2	A, E	2	[56,139]
22	6 (5+1)	Nigeria, Vietnam	2	A, F	2	[126,128]
323	5 (2+3)	Italy, Kenya	2	E, F	2	[135,139]
36	2 (1+1)	Colombia, Kenya	2	F, S	2	[150]
14	34 (27+7)	Bangladesh, China, India, Vietnam	4	A	1	[34,57,106,128]
147	25 (20+5)	Bangladesh, China, India, Pakistan	4	A	1	[34,64,89,100,102]
70	13 (7+6)	Bangladesh, China, Pakistan	3	A	1	[34,88]
16	12 (11+1)	Bangladesh, India, Vietnam	3	A	1	[104,128]
29	8 (2+6)	China, India, Pakistan	3	A	1	[103]
1224^e^	11 (10+1)	China, India	2	A	1	[61,65,103]
334	9	Bangladesh, Vietnam	2	A	1	[34,128]
736^e^	3 (1+2)	Bangladesh, China	2	A	1	[72]

^a^The complete list of all STs of neonatal CRKP is available in S5 Table.

^b^Numbers of strains in literature and those found in genomes only that are underlined are shown in brackets.

^c^Those not reported in literature but found in genomes are underlined.

^d^A, Asia; E, Europe; F, Africa; S, South America.

^e^This ST belongs to *K*. *quasipneumoniae*.

CRKP, carbapenem-resistant *K*. *pneumoniae*; ST, sequence type.

### Most neonatal CRKP strains had genes encoding metallo-β-lactamases, and NDM (New Delhi metallo-β-lactamase) was particularly common

We next investigated the carbapenemase-encoding genes in neonatal CRKP strains. We first examined all included studies and identified 100 studies comprising 1,776 CRKP strains with data on carbapenemases. Carbapenemase genes were reported in 1,388 (78.1%) of the 1,776 strains. We then included 204 genomes not linked to any publications to make up a total of 1,592 strains for analysis of carbapenemases.

Genes encoding 7 known types of carbapenemases, either serine-based (KPC, OXA-23, OXA-48, and SME) or metallo-β-lactamases (MBLs; NDM, IMP, and VIM), and their 26 variants, e.g., KPC-2, NDM-1, and NDM-5, were identified (Table 3). Among the 1,592 neonatal CRKP strains for analysis of carbapenemases, most (*n* = 1,200, 75.3%) encode MBLs. NDM was the most common carbapenemase type, and its encoding gene was identified in 1,024 (64.3%, 1,024/1,592) neonatal CRKP strains, which were recovered in 14 countries (Fig 2). NDM-1 was seen in 643 strains as the most common NDM variant, followed by NDM-5 in 179 strains. KPC (KPC-2, KPC-3, and unspecified variants) and OXA-48-like (OXA-48, OXA-162, OXA-181, and OXA-232) were identified in 327 (20.5%) and 256 (16.1%) strains, representing the second and third most common carbapenemase type in neonatal CRKP, respectively. Of note, 210 strains had genes encoding 2 (*n* = 187) or 3 (*n* = 23) carbapenemases (S7 Table).

**Table 3:** Carbapenemases of CRKP complex in neonates.

Ambler class	Carbapenemases^a^	No. of strains^b^	Country^c^	Reference
A	KPC-2	195	Bangladesh, China, Colombia, India, Vietnam, Venezuela	[34,50,51,53,58,59,62,64,65,68,72,73,102,128,149,150,153]
A	KPC-3	25	Italy	[135–138,140]
A	KPC-nd	109	China, Colombia, Egypt, India, Italy, Portugal, Tunisia	[28,47,66,75,92,109,139,143,152]
A	SME-1	1	China	[64]
A	SME-nd	1	China	[66]
B	IMP-1	1	Japan	[116]
B	IMP-4	62 (59+3)	China	[55,59,68,69,72,74,78,85–87,90,91]
B	IMP-8	13	China	[55,68,74,91]
B	IMP-38	27	China	[51,57,64,71,72]
B	IMP-nd	3	China	[75]
B	MBL-nd	25	India, Pakistan	[101,119]
B	NDM-1	638 (519+119)	Bangladesh, China, Colombia, India, Italy, Kenya, Nepal, Nigeria, Pakistan, South Africa, Vietnam	[34,39,41,43,49,51,53,55–59,64–66,68,70,72,74,77–79,81–85,88,89,97,103–105,107,108,110–112,117,118,122,126,128,133,134,151]
B	NDM-4	58 (57+1)	India, Vietnam, Pakistan	[104,112,128]
B	NDM-5	179 (125+54)	Bangladesh, China, India, Kenya, Nigeria, Vietnam	[34,36,58,67,69,73,76,78,104,106,112,128]
B	NDM-6	1	China	[64]
B	NDM-7	27 (10+17)	Bangladesh, India, Kenya, Nigeria, Pakistan	[34,107]
B	NDM-nd	119 (111+8)	Bangladesh, China, Egypt, India, Iran, Italy, Pakistan, South Africa, UK	[28,29,42,44,75,98,99,114,139,148]
B	VIM-1	20	Italy	[132]
B	VIM-2	14	Venezuela	[153]
B	VIM-12	1 (1)	Greece	
B	VIM-nd	46	China, Egypt, Italy, Portugal, South Africa, Tunisia	[28,44,47,66,142,143]
D	OXA-162	1	Hungary	[131]
D	OXA-181	83 (59+24)	Ghana, India, Kenya, Pakistan, Vietnam	[30,34,100,106,128]
D	OXA-23	23	China	[53]
D	OXA-232	24 (17+7)	Bangladesh, China, India	[34,57,60,96,100,106]
D	OXA-48	110 (107+3)	Algeria, China, Egypt, France, India, Israel, Italy, Morocco, Pakistan, Portugal, Russia, South Africa, Vietnam	[27,29,32,39,42,64,95,97,98,104,115,125,129,133,134,139,143,144]
D	OXA-48-nd	1 (1)	Kenya	

^a^nd, not determined. MBL-nd, metallo-β-lactamases, but the exact types have not been determined. The same rule is also applied for IMP-nd, KPC-nd, NDM-nd, OXA-48-nd, SME-nd, and VIM-nd. Some strains had 2 or more carbapenemases, which are listed in S7 Table.

CRKP, carbapenem-resistant *K*. *pneumoniae*; IMP, imipenemase; KPC, *Klebsiella pneumoniae* carbapenemase; MBL, metallo-β-lactamase; NDM, New Delhi metallo-β-lactamase; OXA, oxacillinase; SME, *Serratia marcescens* enzyme; VIM, Verona integron-encoded metallo-β-lactamase.

^b^Numbers of strains in literature and those found in genomes only that are underlined are shown in brackets.

^c^Those not reported in literature but found in genomes are underlined.

In addition, there are 3 studies that reported CRKP without known carbapenemases [61,63,88]. Two studies have described 23 neonatal CRKP strains, all of which belonged to ST37, but did not specify the mechanisms for carbapenem resistance [61,88]. The other study has reported a single CRKP strain of ST65, in which carbapenem resistance was due to production of the extended-spectrum β-lactamase (ESBL) TEM-53 in combination with decreased expression of outer membrane porins OmpK35 and OmpK36 [63].

We also examined the carbapenemase-encoding plasmids by reviewing literature and examining genomes. Unfortunately, there are no large-scale studies exploring the association of plasmid replicon types and carbapenemase genes for neonatal CRKP. Nonetheless, we found a few common associations (seen in ≥10 strains) of certain carbapenemases with certain plasmid replicon types including KPC-2 with IncN [154], IMP-38 with IncHI5 [155], NDM-1 with IncC [156], IncFI [85], IncFIIK [103], IncHI1B/IncFIB [117], or IncX3 [57,157], NDM-5 with IncX3 [67,76,158], OXA-48 with IncL/M [27,156], OXA-232 with ColKP3 [30,96,159,160], and VIM-1 with IncC [132] (S8 Table). All of these carbapenemase-encoding plasmids except IMP-38-encoding IncHI5, NDM-1-encoding IncHI1B/IncFIB, and VIM-1-encoding IncC were present in neonatal CRKP strains of multiple STs (S8 Table).

### Few neonatal CRKP strains carried hypervirulent determinants

Only 2 studies have reported hypervirulent CRKP with a single strain from each study [63,96]. The 2 strains belonged to either K1 or K2 capsular type, the classical types of hypervirulent *K*. *pneumoniae* [161], had aerobactin-encoding gene *iuc* and the mucoid regulator *rmpA*, and caused bloodstream infection [63,96]. The presence of *iuc* with *rmpA* and/or *rmpA2* (another mucoid regulator) could be used to predict the hypervirulence phenotype [161], and, therefore, we screened their presence for the 535 genomes (results shown in S6 Dataset). We identified 35 (6.5%) possible hypervirulent CRKP strains carrying *iuc* with *rmpA* (*n* = 4), *rmpA2* (*n* = 21), or both (*n* = 10) (S9 Table). These strains were mainly of ST11 with KPC-2 in China, ST15 with KPC-2 in Vietnam, and several STs (ST11, ST15, ST23, and ST231) with NDM-1 or OXA-232 in India (S9 Table). However, the virulence of these strains remains to be determined.

## Discussion

In this study, we found that neonatal CRKP infection has been reported from many countries, in particular LMICs, highlighting a global problem. Importantly, we estimated that the pooled global prevalence of CRKP infections in hospitalized neonates is 0.3% with a 22.9% mortality based on the pooled data. We also found that neonatal CRKP strains are diverse in clonal background, but few had determinants for hypervirulence. Furthermore, we demonstrated that most neonatal CRKP strains have genes encoding MBLs, in particular NDM.

To the best of our knowledge, this is the first comprehensive systematic review to assess neonatal infections caused by CRKP globally to date. About 135 million babies are born annually in the world [162], among which over 15 million new births are early or premature (https://www.gminsights.com/industry-analysis/neonatal-infant-care-market) and therefore are likely to be admitted to hospitals. Global data on neonatal admissions are not available but when considering these early or premature neonates only, there could be at least 60,000 estimated cases of neonatal CRKP infections with at least 5,880 estimated deaths worldwide.

For CRKP in neonates, most (120/146, 82.2%) STs were only seen in a single country (S5 Table). This is likely to be due to the fact that only limited data were available but could also suggest that CRKP seen in neonates have emerged multiple times in many different places, many countries may have their own specific lineages of CRKP in neonates, and most CRKP lineages in neonates are not globally disseminated. Nonetheless, we identified several particularly widespread lineages. ST17 CRKP is of a particular concern as it was identified in neonates in 8 countries of 4 continents and has also caused outbreaks of neonatal infections [81,82]. ST17 CRKP has also been sporadically reported in nonneonatal patients [163,164], animals [165,166], and wastewater [167]. Non-carbapenem-resistant ST17 *K*. *pneumoniae* has been found to commonly carry genes encoding ESBLs [168–173]. The vast majority of neonatal ST17 CRKP strains also carry ESBL genes [30,128], and there were 48 neonatal ST17 CRKP strains with genome sequences available, among which ESBL gene *bla*_CTX-M_ was found in 43 (89.6%; S7 Dataset). This suggests that the ST17 CRKP may emerge as a result of acquisition of carbapenemase genes by ESBL-producing progenitors. Among other commonly identified STs for neonatal CRKP, ST11, ST15, and ST307 have also been frequently seen in adult patients. ST11 is particularly prevalent in Asia and South America and is typically associated with KPC-2 [174,175]. In contrast, neonatal CRKP of ST11 appears to have NDM more commonly than KPC (S6 Dataset). ST15 is an international high-risk lineage associated with a variety of carbapenemases including KPC, NDM, IMP, and OXA-48-like [176–178]. In neonates, ST15 appears to be particularly common in South and Southeast Asia such as Nepal (with NDM-1) [117], Pakistan (with both NDM-1 and OXA-181) [34], and Vietnam (with KPC-2; [128]) but has different carbapenemases. ST307 is another international high-risk lineage, endemic in Colombia, southern Europe (Italy, Portugal, and Spain), South Africa, and Texas of United States of America [179,180], and is associated with various carbapenemases. However, neonatal CRKP of ST307 was mainly reported from Asia (China, Bangladesh, and Vietnam) [72,181] (S5 Table and S6 Dataset). On the other hand, ST258 and its derivate ST512 are widely acknowledged as the major international high-risk type of CRKP in adults [174,182] but were only sporadically found in neonates (S5 Table). The above findings suggest that major CRKP lineages in neonates may be different from those in adults, which warrants further studies. In addition, the common associations of carbapenemases with particular plasmid replicon types in strains of different clonal background suggest possible major vehicles mediating the horizontal transmission of certain carbapenemase-encoding genes, resulting in CRKP affecting neonates. Well-designed epidemiological and genomic studies are required to untangle such plasmid-mediated transmissions of carbapenem resistance to/from and among pathogens for neonatal infections.

As we focused on the prevalence, we did not examine risk factors of neonatal CRKP infection in this study, but such risk factors have been identified in previous studies [44,99,146,183,184]. Some of these risk factors are identical to those for CRKP infection in nonneonatal patients including mechanical ventilation, prolonged length of stay, and prior exposure to antimicrobial agents (in particular carbapenems), while some others are specific for neonates including premature birth, low birth weight, and the use of umbilical vein catheters [44,99,146,183,184]. In this study, we focused on carbapenem resistance, while ESBLs also represent a challenge for clinical management [185]. Although large-scale multisite studies addressing the prevalence of ESBL-producing *K*. *pneumoniae* in neonates are still lacking, several single-site studies have demonstrated a 15% to 90.6% [186–190] prevalence of ESBL genes in neonatal *K*. *pneumoniae* strains. We also found 77.01% of the 535 neonatal CRKP genomes containing genes encoding CTX-M, the major type of ESBLs (S6 Dataset). This suggests a high prevalence, which is not within the scope of the present study but warrants further investigation.

The strength of this study is the comprehensive review of all available studies comprising 127,583 neonates across 30 countries and the hybrid approach of incorporating publicly available genomes of *K*. *pneumoniae* complex of neonatal origins and carrying carbapenemase genes into analysis. Such a hybrid approach allows maximizing the utilization of all data and then generating more extensive and in-depth views of neonatal CRKP infections. We are aware of limitations of this study. First, although we attempted to estimate the prevalence of CRKP infections in all neonates, we were unable to identify relevant studies comprising nonhospitalized ones. As a result, we could only estimate the prevalence of CRKP infections in hospitalized neonates. Second, only a limited number of studies were eligible for analysis. Due to the limited available and obtainable data, we were unable to perform a meta-analysis in addition to a systematic review for the prevalence of neonatal CRKP infections. Third, the geographic coverage of eligible studies is incomplete and biased. Most included studies were from LMICs in Africa and Asia, while no data from North America and Oceania and very few from South America were eligible for analysis. This is largely due to the fact that although CRKP have been well reported in North America and Oceania, these articles reporting CRKP there did not specify whether neonates were involved (e.g., [191,192]), data specific for neonates could not be separated from other populations (e.g., [193,194]), or data on CRKP could not be separated from those on carbapenem-resistant Enterobacterales (CRE) (e.g., [195,196]). To mitigate this problem, we also utilized all publicly available CRKP genomes of neonatal origins for providing complementary information for strain typing and carbapenemases. Notably, 38% genomes included for analysis were not linked to studies pooled for estimating the prevalence, publicly available genomes themselves could be largely biased, and we only included CRKP genomes with carbapenemases. Therefore, the genomes were unable to provide information about the prevalence of neonatal CRKP infections but are still precious sources for providing crucial information for clonal background and carbapenemase types complementing that from literature. Fourth, the included studies were remarkably heterogeneous in quality, design, study populations (NICU and non-NICU), and methods for defining carbapenem resistance, identifying species and typing strains. The heterogeneity generates a wide range of estimated prevalence of neonatal CRKP infections, which was subjected to variations when new data become available. These limitations are primarily driven by data gaps and data sparsity. Well-designed studies of neonatal CRKP infections using standardized protocols are needed to be performed in and reported from more countries, in particular those in Americas, Europe, and Oceania, and such data could be incorporated for future iterations to increase the robustness of estimating the global prevalence. Networks comprising multiple countries such as the BARNARDS network for LMICs [15,34] could be a viable choice. Despite these limitations, we believe that this study is a unique contribution for understanding CRKP in hospitalized neonates and provides much-needed insights for addressing disease burden, informing infection control, and guiding clinical management.

We believe that our findings have important implications for research, surveillance, treatment, and infection control. First, our study highlights that neonatal CRKP infection is a significant burden for public health worldwide. The problem of neonatal CRKP infection may be exaggerated along with the increased consumption of antimicrobial agents. The global antimicrobial consumption in children has increased by 46% between 2000 and 2018 [197], which could be largely attributed to the increase in LMICs [198]. Alarmingly, as pathogens resistant to WHO-recommended ampicillin and gentamicin have been increasingly identified, carbapenems have become the first-line choice for treating neonatal cases of sepsis in some LMICs [2]. Consequently, the emergence of CRKP in neonatal infections is of increasing concern. Therefore, CRKP in neonates warrant further studies and more, large-scale, rigorous surveillance at national or regional levels. Second, unlike the well-known high-risk CRKP lineages ST258 [199], ST11 [200], and ST307 [179], ST17 receives much less attentions. The association of ST17 CRKP with neonates is of particular interest, and more studies are required to elucidate the emergence and dissemination of this lineage in neonates. Third, the finding that most neonatal CRKP strains have genes encoding MBLs could inform treatment. It is worth of pointing out that the distribution of carbapenemases varies across regions, and even hospitals and the prediction of the predominant carbapenemases should be based on the local epidemiology. However, the dominance of MBLs in neonatal CRKP in many parts of the world is alarmingly. Combinations of a β-lactam and a newer β-lactamase inhibitor including ceftazidime-avibactam [201,202], meropenem-vaborbactam [201,202], and imipenem-relebactam [201] are recommended as the major choice to treat infections caused by CRE including CRKP [201]. The newer β-lactamase inhibitors avibactam, vaborbactam, and relebactam have good activities against most serine-based β-lactamases such as carbapenemase KPC but are unable to inhibit MBLs such as IMP, NDM, and VIM [203]. For MBL-producing CRE, ceftazidime-avibactam, meropenem-vaborbactam, and imipenem-relebactam are therefore not effective. The combination of ceftazidime-avibactam and aztreonam, which is stable to the hydrolysis by MBLs [204,205], is recommended against MBL-producing CRE [201]. Cefiderocol is another choice against MBL-producing CRKP but is not available in many countries [201,202]. Polymyxin B or E (colistin) could be an alternative but is commonly associated with poorer outcomes comparing with β-lactams [201]. Nonetheless, the common presence of MBLs in neonatal CRKP strains accentuates the call for developing MBL inhibitors, which have not been approved for clinical use, but significant advances have been made in this realm [206].

In conclusion, we estimated that the pooled prevalence of neonatal CRKP infection was 0.3% (95% CI, 0.2% to 0.3%) with a 22.9% (95% CI, 13.0% to 32.9%) pooled mortality of infected neonates based on literature. We observed very diverse clonal backgrounds of neonatal CRKP strains, which indicates multiple origins. We identified that ST17, ST11, and ST15 were widespread CRKP lineages in neonates, and such lineages warrant rigorous monitoring. We found that most neonatal CRKP strains have genes encoding MBLs, in particular NDM, rendering approved antimicrobial agents containing newer β-lactamase inhibitors such as ceftazidime-avibactam, the mainstream choices against CRKP, ineffective. The above findings highlight that CRKP is a significant threat to neonates, in particular to those in LMICs, and characterize neonatal CRKP infections to inform infection control and treatment. To combat neonatal CRKP infections, rigorous surveillance will lay a foundation by revealing the local epidemiology of the strains and the carbapenemases and needs to be established or enhanced with considering measures like environmental sampling and active screening for those in high risks. For managing neonatal CRKP infections, antimicrobial agents with coverage for MBLs may be the initial choice and then could be tailored when the susceptibility testing results become available.

## Supporting information

S1 Text PRISMA checklist. (DOCX)

S2 Text Protocol for this study. (DOCX)

S1 Fig Species identification methods. MALDI-TOF MS, matrix-assisted laser desorption/ionization time-of-flight mass-spectrometer; mNGS, metagenomic next-generation sequencing; WGS, whole genome sequencing.
(DOCX)

S1 Table Sample types of neonatal CRKP strains. (DOCX)

S2 Table Mortality of CRKP-infected neonates. (DOCX)

S3 Table CRKP colonization rates in neonates. (DOCX)

S4 Table Species identification for strains of CRKP in studies using whole genome sequencing. (DOCX)

S5 Table All STs of neonatal CRKP strains. (DOCX)

S6 Table Profiles of new STs compared with their nearest neighbour. (DOCX)

S7 Table Carbapenemases in neonatal CRKP strains with information for STs. (DOCX)

S8 Table The association of carbapenemases with plasmid replicon types. (DOCX)

S9 Table Strain types and virulence factors of the 35 identified possibly hypervirulent neonatal CRKP strains. (DOCX)

S1 Dataset All included articles. (XLSX)

S2 Dataset Quality assessment for cross-sectional studies. (XLSX)

S3 Dataset Quality assessment for cohort studies. (XLSX)

S4 Dataset Quality assessment for case-control studies. (XLSX)

S5 Dataset Methods for determining carbapenem resistance. (XLSX)

S6 Dataset All 535 genomes. (XLSX)

S7 Dataset ST17 harboring CTX-M. (XLSX)

## Abbreviations

ANI: average nucleotide identity
CI: confidence interval
CRE: carbapenem-resistant Enterobacterales
CRKP: carbapenem-resistant *K*. *pneumoniae*
ESBL: extended-spectrum β-lactamase
IQR: interquartile range
LMIC: low- and middle-income country
MALDI-TOF MS: matrix-assisted laser desorption ionization-time of flight mass spectrometry
MBL: metallo-β-lactamase
NDM: New Delhi metallo-β-lactamase
NICU: neonatal intensive care unit
NOS: Newcastle–Ottawa Scale
SRA: Sequence Read Archive
ST: sequence type
