# Supplementary material for: Prevalence and clonal diversity of carbapenem-resistant Klebsiella pneumoniae causing neonatal infections: A systematic review of 128 articles across 30 countries
Source: PLoS Med. 2023 Jun 20;20(6):e1004233. doi: 10.1371/journal.pmed.1004233 (PMC10281588; doi:10.1371/journal.pmed.1004233)
Supplement: S2 Text — (DOCX) [file pmed.1004233.s002.docx]

S2 Text. Study protocol.

**Prevalence and clonal diversity of carbapenem-resistant *Klebsiella pneumoniae* causing neonatal infections: A systematic review and genomic analysis**

**Study Protocol**

**OBJECTIVES**

This study is to investigate the clinical epidemiology, genetic diversity, and carbapenem resistance of carbapenem-resistant *Klebsiella pneumoniae* (CRKP) causing neonatal infections.

**Primary objective**

To determine clinical epidemiology of CRKP in neonates, the listed outcome variants were included:

- - Prevalence of CRKP infections in neonates
  - Colonization rate of CRKP in neonates
  - Mortality of CRKP infected neonates

**Secondary objective**

To determine molecular epidemiology of CRKP in neonates, the listed outcome variants were included:

- - CRKP precise species
  - CRKP sequence type
  - CRKP carbapenemases

**METHODS**

**Study selection**

Inclusion criteria:

- Study design: All randomized controlled trials, case-control studies, prospective and retrospective cohort studies, cross-section studies, and case reports (only if it supplies molecular data associated to neonatal CRKP)
- Population of interest: neonate or newborn (from birth to 1 month)
- Outcomes:
  - Prevalence of CRKP infections in neonates
  - Colonization rate of CRKP in neonates
  - Mortality of CRKP infected neonates
  - Sample type and infection type
  - CRKP precise species
  - CRKP sequence type
  - CRKP carbapenemases

Exclusion criteria:

- No original data or data duplication
- No neonate or age range did not include neonate
- Neonates could not be separated from study population
- No *Klebsiella pneumoniae* or *Klebsiella pneumoniae* complex
- No carbapenem resistance
- CRKP could not be identified from *Klebsiella pneumoniae* or *Klebsiella pneumoniae* complex or from other groups

**Data abstraction**

The following information will be collected where available:

- Name or title of reports/author/journal
- Year of publication
- Study setting (country, study period)
- Study design
- Randomization process, if any
- Sample size
- Characteristics of study participants (Ward/department, sub-group, if any)
- Outcomes: CRKP infection prevalence, CRKP colonization rate, Mortality of CRKP infected neonates, Sample type and infection type, CRKP sequence type, CRKP carbapenemases

**Search strategy**

Literature search will involve Web of science, Medline, Cochrane, Embase, Pubmed, grey literature from Opengrey, preprint databases and snowball method via searching references of published systematic reviews and other review articles. Search will be limited to literatures with at least title, abstract and key words in English language. Search terms combination will include: “Klebsiella” and “neonate”, “Klebsiella” and “neonatal”, “Klebsiella” and “newborn”, “Klebsiella” and “NICU”, “CRKP” and “neonate”, “CRKP” and “neonatal”, “CRKP” and “newborn”, and “CRKP” and “NICU”.

**Analysis plan**

- The following data will be illustrated (if data is available):
  - Prevalence of CRKP infections in neonates
  - Colonization rate of CRKP in neonates
  - Mortality of CRKP infected neonates
  - Sample type and infection type
  - CRKP precise species
  - CRKP sequence type
  - CRKP carbapenemases
- Quality assessment
  - The Cochrane Risk of Bias tool will be used to assess the quality of all randomized controlled trials
  - The Newcastle Ottawa Scale will be used to assess the quality of cohort studies
  - The quality of evidence for each outcome of interest will be assessed using GRADE methodology

**Data management**

The initial data analysis will address only the objectives specified above. Preliminary findings from these analyses will be circulated among contributing investigators for their comments and suggestions about further analysis. Any additional analysis will be proposed to all investigators, even though related to the objectives above we will not seek unanimous approval of any such additional analyses. However, objections to the new analysis will be addressed and resolved before proceeding. (In other words, if a collaborator does not respond, that is taken as implicit approval). If we think of analyses to address entirely different and novel objectives that are not considered or foreseen here, we will seek approval from ALL investigators before embarking on any such analyses.

All proposed publications will be reviewed and approved by all investigators before public presentation or submission for publication. The authorship will include all responsible investigators contributing data.
