# Supplementary material for: Prevalence and clonal diversity of carbapenem-resistant Klebsiella pneumoniae causing neonatal infections: A systematic review of 128 articles across 30 countries
Source: PLoS Med. 2023 Jun 20;20(6):e1004233. doi: 10.1371/journal.pmed.1004233 (PMC10281588; doi:10.1371/journal.pmed.1004233)

**S1 Fig. Species identification methods.** WGS, whole genome sequencing. mNGS, metagenomic next-generation sequencing. MALDI-TOF MS, Matrix-Assisted Laser Desorption/Ionization Time-of-Flight mass-spectrometer.


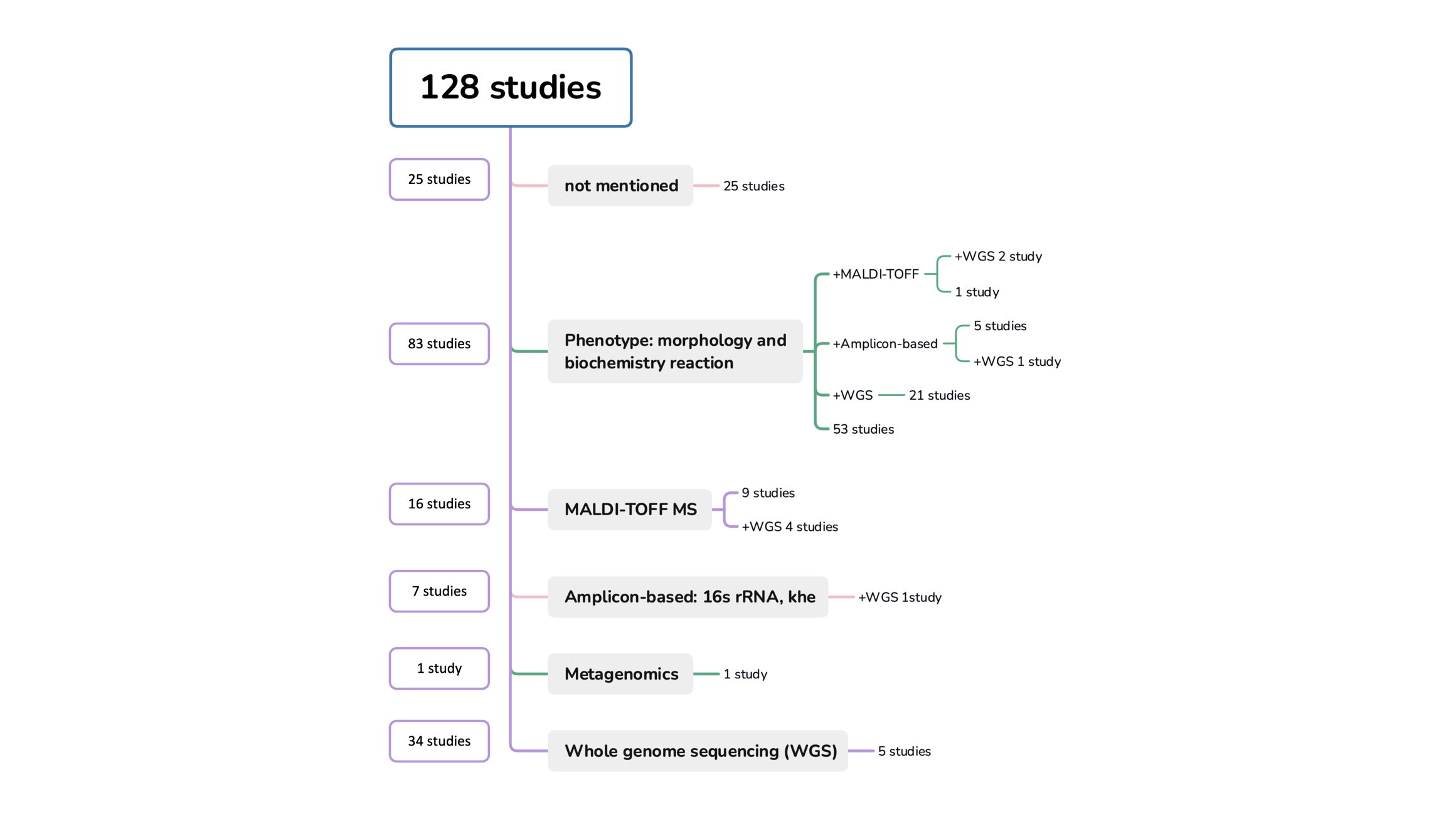

Supplement: S1 Fig — MALDI-TOF MS, matrix-assisted laser desorption/ionization time-of-flight mass-spectrometer; mNGS, metagenomic next-generation sequencing; WGS, whole genome sequencing. (DOCX) [file pmed.1004233.s003.docx]
