# Supplementary material for: Prevalence and clonal diversity of carbapenem-resistant Klebsiella pneumoniae causing neonatal infections: A systematic review of 128 articles across 30 countries
Source: PLoS Med. 2023 Jun 20;20(6):e1004233. doi: 10.1371/journal.pmed.1004233 (PMC10281588; doi:10.1371/journal.pmed.1004233)
Supplement: S1 Table — (DOCX) [file pmed.1004233.s004.docx]

S1 Table. Sample types

| Sample type | no. |
| --- | --- |
| Blood and catheters | 573 |
| Blood | 561 |
| Catheter | 4 |
| Umbilical vein catheter | 2 |
| Umbilicus | 6 |
| Respiratory | 330 |
| Aspiration catheter | 5 |
| Broncho-alveolar lavage | 1 |
| Bronchial aspirate | 1 |
| Endotracheal aspirate | 1 |
| Endotracheal tube | 5 |
| Pharyngeal aspiration | 1 |
| Respiratory secretion | 10 |
| Sputum | 262 |
| Tracheal cannula | 2 |
| Tracheal aspirate | 13 |
| Tracheal fluid | 2 |
| Tracheal intubation | 2 |
| Tracheal intubation head | 2 |
| Tracheal secretion | 22 |
| Tracheal tube | 1 |
| Urinary | 64 |
| Urine | 64 |
| Other clinical sample types | 51 |
| Abdominal fluid | 1 |
| Abdominal wound swab | 1 |
| Ascites | 3 |
| Catheter-associated secretion | 2 |
| CSF | 4 |
| Feces | 11 |
| Gastric aspirate | 3 |
| Pus | 3 |
| Secretion | 5 |
| Stool | 18 |
| Swabs | 431 |
| Anal swab+/-pharyngeal swab | 110 |
| Conjunctival swabs | 1 |
| Nasal swab | 6 |
| Rectal swab | 261 |
| Swabs | 18 |
| Throat swabs | 57 |
| Total | 1,504 |
