# Supplementary material for: Prevalence and clonal diversity of carbapenem-resistant Klebsiella pneumoniae causing neonatal infections: A systematic review of 128 articles across 30 countries
Source: PLoS Med. 2023 Jun 20;20(6):e1004233. doi: 10.1371/journal.pmed.1004233 (PMC10281588; doi:10.1371/journal.pmed.1004233)
Supplement: S2 Table — (DOCX) [file pmed.1004233.s005.docx]

S2 Table. Mortality of CRKP infected neonates

| Study | Year | Country | Study start | Study  end | Ward | no., neonates | | Mortality, % |
| --- | --- | --- | --- | --- | --- | --- | --- | --- |
|  |  |  |  |  |  | died | infected |  |
| [1] | 2019 | China | Jun-2017 | Jun-2017 | NICU | 0 | 5 | 0.000 |
| [2] | 2020 | China | Mar-2015 | Nov-2019 | PICU | 0 | 13 | 0.000 |
| [3] | 2019 | China | Sep-2015 | Sep-2016 | neonatal | 0 | 12 | 0.000 |
| [4] | 2019 | Pakistan | 2010 | 2012 | - | 0 | 2 | 0.000 |
| [5] | 2021 | China | May-2020 | Aug-2020 | NICU | 0 | 1 | 0.000 |
| [6] | 2014 | China | Aug-2012 | Apr-2013 | NICU | 0 | 1 | 0.000 |
| [7] | 2018 | China | Nov-2015 | Oct-2016 | NICU/neonatal | 0 | 39 | 0.000 |
| [8] | 2017 | Italy | Apr-2014 | - | NICU | 0 | 1 | 0.000 |
| [9] | 2016 | China | Oct-2010 | Nov-2012 | NICU/neonatal | 1 | 22 | 4.545 |
| [10] | 2016 | China | Jan-2014 | Mar-2014 | NICU | 1 | 18 | 5.556 |
| [11] | 2017 | Tunisia | Jan-2014 | Dec-2014 | - | 1 | 14 | 7.143 |
| [12] | 2015 | China | Aug-2012 | Mar-2013 | NICU | 1 | 8 | 12.500 |
| [13] | 2019 | Bangladesh | Oct-2016 | Jan-2017 | neonatal | 1 | 6 | 16.667 |
| [14] | 2016 | India | Mar-2016 | Mar-2016 | NICU | 1 | 4 | 25.000 |
| [15] | 2019 | India | Jul-2016 | Dec-2017 | NICU/PICU/neonatal | 3 | 12 | 25.000 |
| [16] | 2021 | Turkey | Jan-2017 | Jul-2018 | NICU | 22 | 70 | 31.429 |
| [17] | 2013 | Colombia | Aug-2011 | Jan-2012 | neonatal | 2 | 6 | 33.333 |
| [18] | 2020 | Egypt | Nov-2015 | Apr-2016 | NICU | 12 | 23 | 52.174 |
| [19] | 2014 | Nepal | Aug-2011 | Jun-2012 | NICU | 16 | 25 | 64.000 |
| [20] | 2022 | India | Apr-2017 | Jul-2017 | NICU | 12 | 14 | 85.714 |
| [21] | 2019 | Morocco | Feb-2015 | Feb-2015 | NICU | 6 | 6 | 100.000 |

References

1. Chen D, Hu X, Chen F, Li H, Wang D, Li X, et al. Co-outbreak of multidrug resistance and a novel ST3006 *Klebsiella pneumoniae* in a neonatal intensive care unit: A retrospective study. Medicine (Baltimore). 2019;98:e14285.

2. Jin C, Shi R, Jiang X, Zhou F, Qiang J, An C. Epidemic characteristics of carbapenem-resistant *Klebsiella pneumoniae* in the pediatric intensive care unit of Yanbian University Hospital, China. Infect Drug Resist. 2020;13:1439-46.

3. Kong Z, Cai R, Cheng C, Zhang C, Kang H, Ma P, et al. First reported nosocomial outbreak of NDM-5-producing *Klebsiella pneumoniae* in a neonatal unit in China. Infect Drug Resist. 2019;12:3557-66.

4. Heinz E, Ejaz H, Bartholdson Scott J, Wang N, Gujaran S, Pickard D, et al. Resistance mechanisms and population structure of highly drug resistant *Klebsiella* in Pakistan during the introduction of the carbapenemase NDM-1. Sci Rep. 2019;9:2392.

5. Weng B, Zhang X, Hong W, Yan C, Gong X, Cai C. A case of sepsis due to carbapenem-resistant *Klebsiella pneumoniae* in an extremely low-birth weight infant treated with trimethoprim-sulfamethoxazole. Infect Drug Resist. 2021;14:2321-5.

6. Ma MS, Wang DH, Sun XJ, Li ZH, Wang C. [Risk factors for *Klebsiella pneumoniae* carbapenemase-producing *Klebsiella pneumoniae* colonization in neonates]. Zhongguo Dang Dai Er Ke Za Zhi. 2014;16:970-4.

7. Yin D, Zhang L, Wang A, He L, Cao Y, Hu F, et al. Clinical and molecular epidemiologic characteristics of carbapenem-resistant *Klebsiella pneumoniae* infection/colonization among neonates in China. J Hosp Infect. 2018;100:21-8.

8. Bonfanti P, Bellù R, Principe L, Caramma I, Condò M, Giani T, et al. Mother-to-child transmission of KPC carbapenemase-producing *Klebsiella pneumoniae* at birth. Pediatr Infect Dis J. 2017;36:228-9.

9. Pang F, Jia XQ, Song ZZ, Li YH, Wang B, Zhao QG, et al. Characteristics and management of *Enterobacteriaceae* harboring IMP-4 or IMP-8 carbapenemase in a tertiary hospital. Afr Health Sci. 2016;16:153-61.

10. Zheng R, Zhang Q, Guo Y, Feng Y, Liu L, Zhang A, et al. Outbreak of plasmid-mediated NDM-1-producing *Klebsiella pneumoniae* ST105 among neonatal patients in Yunnan, China. Ann Clin Microbiol Antimicrob. 2016;15:10.

11. Battikh H, Harchay C, Dekhili A, Khazar K, Kechrid F, Zribi M, et al. Clonal spread of colistin-resistant *Klebsiella pneumoniae* coproducing KPC and VIM carbapenemases in neonates at a Tunisian university hospital. Microb Drug Resist. 2017;23:468-72.

12. Zhang X, Li X, Wang M, Yue H, Li P, Liu Y, et al. Outbreak of NDM-1-producing Klebsiella pneumoniae causing neonatal infection in a teaching hospital in mainland China. Antimicrob Agents Chemother. 2015;59:4349-51.

13. Farzana R, Jones LS, Rahman MA, Andrey DO, Sands K, Portal E, et al. Outbreak of hypervirulent multidrug-resistant *Klebsiella variicola* causing high mortality in neonates in Bangladesh. Clin Infect Dis. 2019;68:1225-7.

14. Singh SK, Gupta M. *bla*_OXA-48_ carrying clonal colistin resistant-carbapenem resistant *Klebsiella pneumoniae* in neonate intensive care unit, India. Microb Pathog. 2016;100:75-7.

15. Mukherjee S, Bhattacharjee A, Naha S, Majumdar T, Debbarma SK, Kaur H, et al. Molecular characterization of NDM-1-producing *Klebsiella pneumoniae* ST29, ST347, ST1224, and ST2558 causing sepsis in neonates in a tertiary care hospital of North-East India. Infect Genet Evol. 2019;69:166-75.

16. Bor M, Ilhan O. Carbapenem-resistant *Klebsiella pneumoniae* outbreak in a neonatal intensive care unit: risk factors for mortality. J Trop Pediatr. 2021;67.

17. Escobar Pérez JA, Olarte Escobar NM, Castro-Cardozo B, Valderrama Márquez IA, Garzón Aguilar MI, Martinez de la Barrera L, et al. Outbreak of NDM-1-producing *Klebsiella pneumoniae* in a neonatal unit in Colombia. Antimicrob Agents Chemother. 2013;57:1957-60.

18. Ghaith DM, Zafer MM, Said HM, Elanwary S, Elsaban S, Al-Agamy MH, et al. Genetic diversity of carbapenem-resistant *Klebsiella pneumoniae* causing neonatal sepsis in intensive care unit, Cairo, Egypt. Eur J Clin Microbiol Infect Dis. 2020;39:583-91.

19. Stoesser N, Giess A, Batty EM, Sheppard AE, Walker AS, Wilson DJ, et al. Genome sequencing of an extended series of NDM-producing *Klebsiella pneumoniae* isolates from neonatal infections in a Nepali hospital characterizes the extent of community- versus hospital-associated transmission in an endemic setting. Antimicrob Agents Chemother. 2014;58:7347-57.

20. Sharma S, Banerjee T, Kumar A, Yadav G, Basu S. Extensive outbreak of colistin resistant, carbapenemase (*bla*_OXA-48_, *bla*_NDM_) producing *Klebsiella pneumoniae* in a large tertiary care hospital, India. Antimicrob Resist Infect Control. 2022;11:1.

21. Taoufik L, Amrani Hanchi A, Fatiha B, Nissrine S, Mrabih Rabou MF, Nabila S. Emergence of OXA-48 carbapenemase producing *Klebsiella pneumoniae* in a neonatal intensive care unit in Marrakech, Morocco. Clin Med Insights Pediatr. 2019;13:1179556519834524.
