# Supplementary material for: Prevalence and clonal diversity of carbapenem-resistant Klebsiella pneumoniae causing neonatal infections: A systematic review of 128 articles across 30 countries
Source: PLoS Med. 2023 Jun 20;20(6):e1004233. doi: 10.1371/journal.pmed.1004233 (PMC10281588; doi:10.1371/journal.pmed.1004233)
Supplement: S3 Table — (DOCX) [file pmed.1004233.s006.docx]

S3 Table. CRKP colonization rate

| Study | Country | Start | End | Ward | No.,  beds | No., neonatal | | Colonization rate, % | Sample type (no.) |
| --- | --- | --- | --- | --- | --- | --- | --- | --- | --- |
|  |  |  |  |  |  | screened | CRKP +ve |  |  |
| [1] | China | Jan-2017 | Dec-2018 | NICU | - | 1,230 | 110 | 8.943 | Rectal swabs, pharyngeal swabs,  rectal & pharyngeal swabs^a^ |
| [2] | China | Aug-2012 | Apr-2013 | NICU | - | 474 | 9 | 1.899 | rectal swabs (7), throat swab (2) |
| [3] | Turkey | Jan-2010 | Dec-2014 | PICU/NICU | - | 1,671 | 44 | 2.633 | rectal swabs (44) |
| [4] | China | Jul-2017 | Jun-2018 | NICU | 200 | 5,348 | 6 | 0.112 | sputum (6) |
| [5] | Vietnam | - | - | - | - | 326 | 16 | 4.908:  1.840 at admission  3.681 at discharge | rectal swabs (18):  6 at admission,  12 at discharge |
| [6] | India | 2017 | 2018 | SNCU^b^ | - | 38 | 4 | 10.526 | nasal swabs (7) |
| [7] | Colombia | Feb-2019 | Apr-2012 | NICU | - | 57 | 13 | 22.807 | rectal swabs (13) |
| [8] | Turkey | Jun-2012 | Dec-2012 | - | - | 145 | 45 | 31.034 | rectal swabs (45) |
| [9] | Italy | Sep-2012 | Nov-2012 | NICU | 16 | 54 | 10 | 18.500 | rectal swabs (10) |
| [10] | Portugal | Mar-2019 | Oct-2019 | NICU | 14 | 173 | 7 | 4.046 | rectal swabs (7) |
| [11] | Algeria | Jan-2017 | Apr-2017 | - | - | 422 | 7 | 1.659 | rectal swabs (7) |

^a^Numbers of each type of swabs are not available.

^b^SNCU, sick neonatal care unit.

References

1. Yin L, He L, Miao J, Yang W, Wang X, Ma J, et al. Carbapenem-resistant Enterobacterales colonization and subsequent infection in a neonatal intensive care unit in Shanghai, China. Infect Prev Pract. 2021;3:100147.

2. Ma MS, Wang DH, Sun XJ, Li ZH, Wang C. [Risk factors for *Klebsiella pneumoniae* carbapenemase-producing *Klebsiella pneumoniae* colonization in neonates]. Zhongguo Dang Dai Er Ke Za Zhi. 2014;16:970-4.

3. Akturk H, Sutcu M, Somer A, Aydin D, Cihan R, Ozdemir A, et al. Carbapenem-resistant *Klebsiella pneumoniae* colonization in pediatric and neonatal intensive care units: risk factors for progression to infection. Braz J Infect Dis. 2016;20:134-40.

4. Zhou J, Yang J, Hu F, Gao K, Sun J, Yang J. Clinical and molecular epidemiologic characteristics of ceftazidime/avibactam-resistant carbapenem-resistant *Klebsiella pneumoniae* in a neonatal intensive care unit in China. Infect Drug Resist. 2020;13:2571-8.

5. Kk S, Ekedahl E, Hoang NTB, Sewunet T, Berglund B, Lundberg L, et al. High diversity of *bla*_NDM-1_-encoding plasmids in *Klebsiella pneumoniae* isolated from neonates in a Vietnamese hospital. Int J Antimicrob Agents. 2022;59:106496.

6. Bhattacharjee B, Bardhan T, Chakraborty M, Basu M. Resistance profiles and resistome mapping of multidrug resistant carbapenem-hydrolyzing *Klebsiella pneumoniae* strains isolated from the nares of preterm neonates. Int J Antimicrob Agents. 2019;53:535-7.

7. Magda Sánchez DJM. Detección rápida de *Enterobacterias* productoras de carbapenemasas en hisopados rectales de pacientes neonatos colonizados. Infection. 2021;25:89-92.

8. Ulu-Kilic A, Alp E, Percin D, Cevahir F, Altay-Kürkçü C, Ozturk A, et al. Risk factors for carbapenem resistant *Klebsiella pneumoniae* rectal colonization in pediatric units. J Infect Dev Ctries. 2014;8:1361-4.

9. Giuffrè M, Bonura C, Geraci DM, Saporito L, Catalano R, Di Noto S, et al. Successful control of an outbreak of colonization by *Klebsiella pneumoniae* carbapenemase-producing *K. pneumoniae* sequence type 258 in a neonatal intensive care unit, Italy. J Hosp Infect. 2013;85:233-6.

10. Almeida TL, Mendo T, Costa R, Novais C, Marçal M, Martins F, et al. Carbapenemase-producing *Enterobacteriaceae* (CPE) newborn colonization in a Portuguese neonatal intensive care unit (NICU): epidemiology and infection prevention and control measures. Infect Dis Rep. 2021;13:411-7.

11. Mairi A, Touati A, Ait Bessai S, Boutabtoub Y, Khelifi F, Sotto A, et al. Carbapenemase-producing *Enterobacteriaceae* among pregnant women and newborns in Algeria: Prevalence, molecular characterization, maternal-neonatal transmission, and risk factors for carriage. Am J Infect Control. 2019;47:105-8.
