# Supplementary material for: Prevalence and clonal diversity of carbapenem-resistant Klebsiella pneumoniae causing neonatal infections: A systematic review of 128 articles across 30 countries
Source: PLoS Med. 2023 Jun 20;20(6):e1004233. doi: 10.1371/journal.pmed.1004233 (PMC10281588; doi:10.1371/journal.pmed.1004233)
Supplement: S4 Table — (DOCX) [file pmed.1004233.s007.docx]

S4 Table. Species identification for strains of carbapenem-resistant *Klebsiella pneumoniae* complex in 33 studies using whole genome sequencing

| Methods | No., CRKP | Precise species: *K.* | | | Reference |
| --- | --- | --- | --- | --- | --- |
|  |  | *pneumoniae* | *quasipneumoniae* | *variicola* |  |
| ANI | 32 | 27 | 5 | - | [1-3] |
| A k-mer-based tool in CLC Genomics Workbench | 18 | 15 | 3 | - | [4] |
| Kraken, a kmer-based tool | 47 | 47 | - | - | [5] |
| phylogeny-based, PCA-based | 18 | 17 | 1 | - | [6] |
| Speciator, a mash-based tool available on PathogenWatch | 76 | 72 | 4 | - | [7-9] |
| WGS-based species identification but methods not specified | 193 | 180 | 9 | 4 | [10-34] |

References

1. Brinkac LM, White R, D'Souza R, Nguyen K, Obaro SK, Fouts DE. Emergence of New Delhi metallo-β-Lactamase (NDM-5) in *Klebsiella quasipneumoniae* from neonates in a Nigerian hospital. mSphere. 2019;4.

2. Qiao F, Wei L, Feng Y, Ran S, Zheng L, Zhang Y, et al. Handwashing sink contamination and carbapenem-resistant *Klebsiella* infection in the intensive care unit: a prospective multicenter study. Clin Infect Dis. 2020;71:S379-s85.

3. Wei L, Feng Y, Wen H, Ya H, Qiao F, Zong Z. NDM-5-producing carbapenem-resistant *Klebsiella pneumoniae* of sequence type 789 emerged as a threat for neonates: a multicentre, genome-based study. Int J Antimicrob Agents. 2022;59:106508.

4. Kk S, Ekedahl E, Hoang NTB, Sewunet T, Berglund B, Lundberg L, et al. High diversity of *bla*_NDM-1_-encoding plasmids in *Klebsiella pneumoniae* isolated from neonates in a Vietnamese hospital. Int J Antimicrob Agents. 2022;59:106496.

5. Stoesser N, Giess A, Batty EM, Sheppard AE, Walker AS, Wilson DJ, et al. Genome sequencing of an extended series of NDM-producing *Klebsiella pneumoniae* isolates from neonatal infections in a Nepali hospital characterizes the extent of community- versus hospital-associated transmission in an endemic setting. Antimicrob Agents Chemother. 2014;58:7347-57.

6. Pei N, Li Y, Liu C, Jian Z, Liang T, Zhong Y, et al. Large-scale genomic epidemiology of *Klebsiella pneumoniae* identified clone divergence with hypervirulent plus antimicrobial-resistant characteristics causing within-ward strain transmissions. Microbiol Spectr. 2022;10:e0269821.

7. Sands K, Carvalho MJ, Portal E, Thomson K, Dyer C, Akpulu C, et al. Characterization of antimicrobial-resistant Gram-negative bacteria that cause neonatal sepsis in seven low- and middle-income countries. Nat Microbiol. 2021;6:512-23.

8. Nagaraj G, Shamanna V, Govindan V, Rose S, Sravani D, Akshata KP, et al. High-resolution genomic profiling of carbapenem-resistant *Klebsiella pneumoniae* isolates: a multicentric retrospective Indian study. Clin Infect Dis. 2021;73:S300-s7.

9. Saavedra SY, Bernal JF, Montilla-Escudero E, Arévalo SA, Prada DA, Valencia MF, et al. Complexity of genomic epidemiology of carbapenem-resistant *Klebsiella pneumoniae* isolates in Colombia urges the reinforcement of whole genome sequencing-based surveillance programs. Clin Infect Dis. 2021;73:S290-s9.

10. Principe L, Meroni E, Conte V, Mauri C, Di Pilato V, Giani T, et al. Mother-to-child transmission of KPC-producing *Klebsiella pneumoniae*: potential relevance of a low microbial urinary load for screening purposes. J Hosp Infect. 2018;98:314-6.

11. Berglund B, Hoang NTB, Tärnberg M, Le NK, Welander J, Nilsson M, et al. Colistin- and carbapenem-resistant *Klebsiella pneumoniae* carrying *mcr-1* and *bla*_OXA-48_ isolated at a paediatric hospital in Vietnam. J Antimicrob Chemother. 2018;73:1100-2.

12. Liu J, Yu J, Chen F, Yu J, Simner P, Tamma P, et al. Emergence and establishment of KPC-2-producing ST11 Klebsiella pneumoniae in a general hospital in Shanghai, China. Eur J Clin Microbiol Infect Dis. 2018;37:293-9.

13. Maida CM, Bonura C, Geraci DM, Graziano G, Carattoli A, Rizzo A, et al. Outbreak of ST395 KPC-producing *Klebsiella pneumoniae* in a neonatal intensive care unit in Palermo, Italy. Infect Control Hosp Epidemiol. 2018;39:496-8.

14. Monaco F, Mento GD, Cuscino N, Conaldi PG, Douradinha B. Infant colonisation with *Escherichia coli* and *Klebsiella pneumoniae* strains co-harbouring *bla*_OXA-48_ and *bla*_NDM-1_ carbapenemases genes: a case report. Int J Antimicrob Agents. 2018;52:121-2.

15. Farzana R, Jones LS, Rahman MA, Andrey DO, Sands K, Portal E, et al. Outbreak of hypervirulent multidrug-resistant *Klebsiella variicola* causing high mortality in neonates in Bangladesh. Clin Infect Dis. 2019;68:1225-7.

16. Bhattacharjee B, Bardhan T, Chakraborty M, Basu M. Resistance profiles and resistome mapping of multidrug resistant carbapenem-hydrolyzing *Klebsiella pneumoniae* strains isolated from the nares of preterm neonates. Int J Antimicrob Agents. 2019;53:535-7.

17. Chen D, Hu X, Chen F, Li H, Wang D, Li X, et al. Co-outbreak of multidrug resistance and a novel ST3006 *Klebsiella pneumoniae* in a neonatal intensive care unit: A retrospective study. Medicine (Baltimore). 2019;98:e14285.

18. Heinz E, Ejaz H, Bartholdson Scott J, Wang N, Gujaran S, Pickard D, et al. Resistance mechanisms and population structure of highly drug resistant *Klebsiella* in Pakistan during the introduction of the carbapenemase NDM-1. Sci Rep. 2019;9:2392.

19. Gona F, Bongiorno D, Aprile A, Corazza E, Pasqua B, Scuderi MG, et al. Emergence of two novel sequence types (3366 and 3367) NDM-1- and OXA-48-co-producing *K. pneumoniae* in Italy. Eur J Clin Microbiol Infect Dis. 2019;38:1687-91.

20. Li J, Hu X, Yang L, Lin Y, Liu Y, Li P, et al. New Delhi metallo-β-Lactamase 1-producing *Klebsiella pneumoniae* ST719 isolated from a neonate in China. Microb Drug Resist. 2020;26:492-6.

21. Naha S, Sands K, Mukherjee S, Roy C, Rameez MJ, Saha B, et al. KPC-2-producing *Klebsiella pneumoniae* ST147 in a neonatal unit: Clonal isolates with differences in colistin susceptibility attributed to AcrAB-TolC pump. Int J Antimicrob Agents. 2020;55:105903.

22. Ramsamy Y, Mlisana KP, Allam M, Amoako DG, Abia ALK, Ismail A, et al. Genomic analysis of carbapenemase-producing extensively drug-resistant *Klebsiella pneumoniae* isolates reveals the horizontal spread of p18-43_01 plasmid encoding *bla*_NDM-1_ in South Africa. Microorganisms. 2020;8.

23. Mukherjee S, Naha S, Bhadury P, Saha B, Dutta M, Dutta S, et al. Emergence of OXA-232-producing hypervirulent *Klebsiella pneumoniae* ST23 causing neonatal sepsis. J Antimicrob Chemother. 2020;75:2004-6.

24. Wang S, Zhao J, Liu N, Yang F, Zhong Y, Gu X, et al. IMP-38-producing high-risk sequence type 307 *Klebsiella pneumoniae* strains from a neonatal unit in China. mSphere. 2020;5.

25. Labi AK, Nielsen KL, Marvig RL, Bjerrum S, Enweronu-Laryea C, Bennedbæk M, et al. Oxacillinase-181 carbapenemase-producing *Klebsiella pneumoniae* in neonatal intensive care unit, Ghana, 2017-2019. Emerg Infect Dis. 2020;26:2235-8.

26. Rada AM, De La Cadena E, Agudelo C, Capataz C, Orozco N, Pallares C, et al. Dynamics of *bla*_KPC-2_ dissemination from non-CG258 *Klebsiella pneumoniae* to other *Enterobacterales* via IncN plasmids in an area of high endemicity. Antimicrob Agents Chemother. 2020;64.

27. Kopotsa K, Mbelle NM, Osei Sekyere J. Epigenomics, genomics, resistome, mobilome, virulome and evolutionary phylogenomics of carbapenem-resistant *Klebsiella pneumoniae* clinical strains. Microb Genom. 2020;6.

28. Luo K, Tang J, Qu Y, Yang X, Zhang L, Chen Z, et al. Nosocomial infection by *Klebsiella pneumoniae* among neonates: a molecular epidemiological study. J Hosp Infect. 2021;108:174-80.

29. Naha S, Sands K, Mukherjee S, Saha B, Dutta S, Basu S. OXA-181-like carbapenemases in *Klebsiella pneumoniae* ST14, ST15, ST23, ST48, and ST231 from septicemic neonates: coexistence with NDM-5, resistome, transmissibility, and genome diversity. mSphere. 2021;6.

30. Abe R, Oyama F, Akeda Y, Nozaki M, Hatachi T, Okamoto Y, et al. Hospital-wide outbreaks of carbapenem-resistant *Enterobacteriaceae* horizontally spread through a clonal plasmid harbouring *bla*_IMP-1_ in children's hospitals in Japan. J Antimicrob Chemother. 2021;76:3314-7.

31. Berglund B, Hoang NTB, Lundberg L, Le NK, Tärnberg M, Nilsson M, et al. Clonal spread of carbapenem-resistant *Klebsiella pneumoniae* among patients at admission and discharge at a Vietnamese neonatal intensive care unit. Antimicrob Resist Infect Control. 2021;10:162.

32. Bai Y, Shao C, Hao Y, Wang Y, Jin Y. Using whole genome sequencing to trace, control and characterize a hospital infection of IMP-4-producing *Klebsiella pneumoniae* ST2253 in a neonatal unit in a tertiary hospital, China. Front Public Health. 2021;9:755252.

33. Cienfuegos-Gallet AV, Zhou Y, Ai W, Kreiswirth BN, Yu F, Chen L. Multicenter genomic analysis of carbapenem-resistant *Klebsiella pneumoniae* from bacteremia in China. Microbiol Spectr. 2022;10:e0229021.

34. Dubodelov DV, Lubasovskaya LA, Shubina ES, Mukosey IS, Korostin DO, Kochetkova TO, et al. [Genetic determinants of resistance of hospital-associated strains of *Klebsiella pneumoniae* to β-lactam antibiotics isolated in neonates]. Genetika. 2016;52:1097-102.
