# Supplementary material for: Prevalence and clonal diversity of carbapenem-resistant Klebsiella pneumoniae causing neonatal infections: A systematic review of 128 articles across 30 countries
Source: PLoS Med. 2023 Jun 20;20(6):e1004233. doi: 10.1371/journal.pmed.1004233 (PMC10281588; doi:10.1371/journal.pmed.1004233)
Supplement: S5 Table — (DOCX) [file pmed.1004233.s008.docx]

S5 Table. All sequence types (STs) of carbapenem-resistant *Klebsiella pneumoniae* from neonates^a^

| ST | No.,  strains^a^ | Country |  | Continent | | Reference |
| --- | --- | --- | --- | --- | --- | --- |
|  |  | List^b^ | No. | List^c^ | No. |  |
| 17 | 136 (117+19) | Bangladesh, China, Colombia, Ghana, Greece, Italy, Kenya, Vietnam | 8 | A/E/F/S | 4 | [1-12] |
| 11 | 123 (108+15) | Bangladesh, China, India, Italy, Nepal, Nigeria, Pakistan, Vietnam | 8 | A/E/F | 3 | [7-11, 13-21] |
| 54 | 45 (38+7) | China, Italy, Kenya | 3 | A/E/F | 3 | [3, 5, 7, 22-24] |
| 101 | 11 | China, Colombia, Italy | 3 | A/E/S | 3 | [7, 25-27] |
| 15 | 183 (160+23) | China, Hungary, India, Nepal, Pakistan, Vietnam | 6 | A/E | 2 | [8-10, 28-34] |
| 307 | 42 (39+3) | Bangladesh, China, Italy, Vietnam | 4 | A/E | 2 | [7, 15, 19, 21, 35-39] |
| 20 | 48 (40+8) | China, Nigeria, Pakistan | 3 | A/F | 2 | [3, 5, 10, 11, 16, 22, 24] |
| 39 | 31 (30+1) | Israel, Nigeria, South Africa | 3 | A/F | 2 | [40, 41] |
| 395 | 14 | Italy, Russia, Nigeria | 3 | E/F | 2 | [7, 10, 39, 42] |
| 48 | 10 (3+7) | Ghana, India, Pakistan | 3 | A/F | 2 | [6, 32] |
| 35 | 4 (2+2) | Bangladesh, China, Italy | 3 | A/E | 2 | [7, 17] |
| 231 | 3 (2+1) | Bangladesh, India, Italy | 3 | A/E | 2 | [7, 32] |
| 45 | 17 | Algeria, China | 2 | A/F | 2 | [11, 12, 15, 43] |
| 1043 | 7 | Colombia, Vietnam | 2 | A/S | 2 | [9, 44] |
| 1412 | 6 | China, Italy | 2 | A/E | 2 | [7, 45] |
| 22 | 6 (5+1) | Nigeria, Vietnam | 2 | A/F | 2 | [8, 9] |
| 323 | 5 (2+3) | Italy, Kenya | 2 | E/F | 2 | [7, 38] |
| 36 | 2 (1+1) | Colombia, Kenya | 2 | F/S | 2 | [25] |
| 14 | 34 (27+7) | Bangladesh, China, India, Vietnam | 4 | A | 1 | [8, 10, 32, 34] |
| 147 | 25 (20+5) | Bangladesh, China, India, Pakistan | 4 | A | 1 | [1, 10, 19, 46, 47] |
| 70 | 13 (7+6) | Bangladesh, China, Pakistan | 3 | A | 1 | [10, 22] |
| 16 | 12 (11+1) | Bangladesh, India, Vietnam | 3 | A | 1 | [8, 31] |
| 29 | 8 (2+6) | China, India, Pakistan | 3 | A | 1 | [48] |
| 1224^d^ | 11 (10+1) | China, India | 2 | A | 1 | [20, 48, 49] |
| 334 | 9 | Bangladesh, Vietnam | 2 | A | 1 | [8, 10] |
| 736^d^ | 3 (1+2) | Bangladesh, China | 2 | A | 1 | [21] |
| 789 | 69 (40+29) | China | 1 | A | 1 | [50, 51] |
| 278 | 52 (51+1) | China | 1 | A | 1 | [11, 12, 34] |
| 37 | 41 (35+6) | China | 1 | A | 1 | [13, 22, 34, 49, 52] |
| 4144 | 26 | China | 1 | A | 1 | [11] |
| 104 | 20 | Italy | 1 | E | 1 | [53] |
| 105 | 18 | China | 1 | A | 1 | [54] |
| 5235 | 18 | India | 1 | A | 1 | [55, 56] |
| 833 | 14 | Venezuela | 1 | S | 1 | [57] |
| 12 | 12 | China | 1 | A | 1 | [1] |
| 337 | 12 | China | 1 | A | 1 | [58] |
| 258 | 10 | Italy | 1 | E | 1 | [59] |
| 502 | 10 | Colombia | 1 | S | 1 | [25] |
| 464 | 9 (7+2) | Nigeria | 1 | F | 1 | [10] |
| 76 | 9 | China | 1 | A | 1 | [17, 52] |
| 268 | 9 | Iran | 1 | A | 1 | [60] |
| 2253 | 7 | China | 1 | A | 1 | [61] |
| 2735 | 7 | China | 1 | A | 1 | [34] |
| 43 | 6 | Iran | 1 | A | 1 | [60] |
| 244 | 6 | Bangladesh | 1 | A | 1 |  |
| 442 | 6 | Nigeria | 1 | F | 1 | [10] |
| 476 | 6 | Nigeria | 1 | F | 1 | [62] |
| 3350 | 6 | China | 1 | A | 1 | [15, 21] |
| 13 | 5 | Algeria | 1 | F | 1 | [43] |
| 347 | 5 | India | 1 | A | 1 | [48] |
| 771^e^ | 5 | Bangladesh | 1 | A | 1 | [63] |
| 2407 | 5 | China | 1 | A | 1 |  |
| 3003 | 5 | Vietnam | 1 | A | 1 | [8, 9] |
| 394 | 4 | Bangladesh | 1 | A | 1 |  |
| 397 | 4 | Vietnam | 1 | A | 1 | [8] |
| 705 | 4 | China | 1 | A | 1 | [3, 24] |
| 611 | 3 (2+1) | Pakistan | 1 | A | 1 | [10] |
| 2355^d^ | 3 (1+2) | Bangladesh | 1 | A | 1 | [10] |
| 23 | 3 | India | 1 | A | 1 | [32, 64] |
| 140 | 3 | Colombia | 1 | S | 1 | [4, 25] |
| 512 | 3 | Italy | 1 | E | 1 | [7, 65] |
| 571^d^ | 3 | Nigeria | 1 | F | 1 |  |
| 995 | 3 | Pakistan | 1 | A | 1 | [10] |
| 1998^d^ | 3 | Bangladesh | 1 | A | 1 |  |
| 3366 | 3 | Italy | 1 | E | 1 | [26] |
| 4141 | 3 | China | 1 | A | 1 | [11] |
| 4410 | 3 | Nigeria | 1 | F | 1 | [10] |
| 1031^d^ | 2 (1+1) | Nigeria | 1 | F | 1 | [62] |
| 11V1 | 2 | Kenya | 1 | F | 1 |  |
| 24 | 2 | Bangladesh | 1 | A | 1 |  |
| 42 | 2 | Kenya | 1 | F | 1 |  |
| 234 | 2 | China | 1 | A | 1 | [45] |
| 290 | 2 | China | 1 | A | 1 | [3, 24] |
| 485 | 2 | China | 1 | A | 1 | [19] |
| 726 | 2 | Pakistan | 1 | A | 1 |  |
| 1419 | 2 | China | 1 | A | 1 | [13, 27] |
| 2236 | 2 | China | 1 | A | 1 | [21] |
| 2558 | 2 | India | 1 | A | 1 | [48] |
| 2736 | 2 | China | 1 | A | 1 | [34] |
| 2738 | 2 | China | 1 | A | 1 | [34] |
| 3344 | 2 | India | 1 | A | 1 | [31] |
| 3367 | 2 | Italy | 1 | E | 1 | [26] |
| 4132 | 2 | China | 1 | A | 1 | [11] |
| 4134 | 2 | China | 1 | A | 1 | [11] |
| 4145 | 2 | China | 1 | A | 1 | [11] |
| 5015 | 2 | Vietnam | 1 | A | 1 | [9] |
| 15V1 | 1 | Pakistan | 1 | A | 1 | [10] |
| 15V2 | 1 | Pakistan | 1 | A | 1 | [10] |
| 15V3 | 1 | Pakistan | 1 | A | 1 |  |
| 15V4 | 1 | Pakistan | 1 | A | 1 | [10] |
| 25 | 1 | India | 1 | A | 1 | [10] |
| 34 | 1 | China | 1 | A | 1 | [17] |
| 38 | 1 | Kenya | 1 | F | 1 |  |
| 64 | 1 | China | 1 | A | 1 | [19] |
| 65 | 1 | China | 1 | A | 1 | [66] |
| 152 | 1 | South Africa | 1 | F | 1 | [67] |
| 160 | 1 | Italy | 1 | E | 1 | [7] |
| 188 | 1 | China | 1 | A | 1 | [34] |
| 241 | 1 | China | 1 | A | 1 | [13] |
| 324 | 1 | China | 1 | A | 1 | [13] |
| 340 | 1 | Bangladesh | 1 | A | 1 |  |
| 377 | 1 | Nigeria | 1 | F | 1 | [10] |
| 413 | 1 | Kenya | 1 | F | 1 |  |
| 433 | 1 | China | 1 | A | 1 | [2] |
| 437 | 1 | India | 1 | A | 1 | [46] |
| 462 | 1 | China | 1 | A | 1 | [15] |
| 464V1 | 1 | Nigeria | 1 | F | 1 | [10] |
| 466 | 1 | Italy | 1 | E | 1 | [7] |
| 477^d^ | 1 | Nigeria | 1 | F | 1 | [10] |
| 628 | 1 | China | 1 | A | 1 |  |
| 657 | 1 | India | 1 | A | 1 | [31] |
| 711 | 1 | India | 1 | A | 1 | [68] |
| 719 | 1 | China | 1 | A | 1 | [69] |
| 778 | 1 | China | 1 | A | 1 | [19] |
| 782 | 1 | China | 1 | A | 1 | [13] |
| 784 | 1 | China | 1 | A | 1 | [13] |
| 846 | 1 | China | 1 | A | 1 | [12] |
| 873 | 1 | India | 1 | A | 1 | [31] |
| 888 | 1 | China | 1 | A | 1 | [16] |
| 915V1 | 1 | Japan | 1 | A | 1 | [70] |
| 967 | 1 | India | 1 | A | 1 | [10] |
| 997 | 1 | Bangladesh | 1 | A | 1 |  |
| 1027 | 1 | China | 1 | A | 1 | [50] |
| 1164 | 1 | Italy | 1 | E | 1 | [7] |
| 1310 | 1 | Pakistan | 1 | A | 1 |  |
| 1317 | 1 | Pakistan | 1 | A | 1 |  |
| 1322 | 1 | Pakistan | 1 | A | 1 |  |
| 1710 | 1 | China | 1 | A | 1 | [13] |
| 1878 | 1 | Algeria | 1 | F | 1 | [43] |
| 2053 | 1 | Colombia | 1 | S | 1 | [4] |
| 2194 | 1 | China | 1 | A | 1 | [13] |
| 2534 | 1 | India | 1 | A | 1 | [10] |
| 2574 | 1 | China | 1 | A | 1 | [13] |
| 2816 | 1 | India | 1 | A | 1 | [68] |
| 2823 | 1 | China | 1 | A | 1 | [19] |
| 3228 | 1 | Vietnam | 1 | A | 1 | [9] |
| 3348V1 | 1 | Pakistan | 1 | A | 1 |  |
| 3444 | 1 | China | 1 | A | 1 | [1] |
| 4494 | 1 | Bangladesh | 1 | A | 1 |  |
| 4511 | 1 | Bangladesh | 1 | A | 1 |  |
| 4854 | 1 | China | 1 | A | 1 | [17] |
| 4979^d^ | 1 | Nigeria | 1 | F | 1 |  |
| 4981 | 1 | Pakistan | 1 | A | 1 |  |
| 5014 | 1 | Vietnam | 1 | A | 1 | [9] |
| 5016 | 1 | Vietnam | 1 | A | 1 | [9] |
| 5017 | 1 | Vietnam | 1 | A | 1 | [9] |

^a^Numbers of strains in literature and those found in genomes only that are underlined are shown in brackets.

^b^Those not reported in literature but found in genomes are underlined.

^c^A, Asia; E, Europe; F, Africa; S, South America.

^d^This ST belongs to *Klebsiella quasipneumoniae*

^e^This ST belongs to *Klebsiella variicola*

References

1. Zou H, Shen Y, Li C, Li Q. Two phenotypes of *Klebsiella pneumoniae* ST147 outbreak from neonatal sepsis with a slight increase in virulence. Infect Drug Resist. 2022;15:1-12. Epub 2022/01/14. doi: 10.2147/idr.S343292. PubMed PMID: 35023933; PubMed Central PMCID: PMCPMC8748007.

2. Zhang X, Li X, Wang M, Yue H, Li P, Liu Y, et al. Outbreak of NDM-1-producing Klebsiella pneumoniae causing neonatal infection in a teaching hospital in mainland China. Antimicrob Agents Chemother. 2015;59(7):4349-51. Epub 2015/05/06. doi: 10.1128/AAC.03868-14. PubMed PMID: 25941224; PubMed Central PMCID: PMCPMC4468712.

3. Jin Y, Song X, Liu Y, Wang Y, Zhang B, Fan H, et al. Characteristics of carbapenemase-producing *Klebsiella pneumoniae* as a cause of neonatal infection in Shandong, China. Exp Ther Med. 2017;13(3):1117-26. Epub 2017/04/30. doi: 10.3892/etm.2017.4070. PubMed PMID: 28450951; PubMed Central PMCID: PMCPMC5403258.

4. Saavedra SY, Bernal JF, Montilla-Escudero E, Arévalo SA, Prada DA, Valencia MF, et al. Complexity of genomic epidemiology of carbapenem-resistant *Klebsiella pneumoniae* isolates in Colombia urges the reinforcement of whole genome sequencing-based surveillance programs. Clin Infect Dis. 2021;73(Suppl_4):S290-s9. Epub 2021/12/02. doi: 10.1093/cid/ciab777. PubMed PMID: 34850835; PubMed Central PMCID: PMCPMC8634422.

5. Jin Y, Shao C, Li J, Fan H, Bai Y, Wang Y. Outbreak of multidrug resistant NDM-1-producing Klebsiella pneumoniae from a neonatal unit in Shandong Province, China. PLoS One. 2015;10(3):e0119571. Epub 2015/03/24. doi: 10.1371/journal.pone.0119571. PubMed PMID: 25799421; PubMed Central PMCID: PMCPMC4370709.

6. Labi AK, Nielsen KL, Marvig RL, Bjerrum S, Enweronu-Laryea C, Bennedbæk M, et al. Oxacillinase-181 carbapenemase-producing *Klebsiella pneumoniae* in neonatal intensive care unit, Ghana, 2017-2019. Emerg Infect Dis. 2020;26(9):2235-8. Epub 2020/08/21. doi: 10.3201/eid2609.200562. PubMed PMID: 32818427; PubMed Central PMCID: PMCPMC7454046.

7. Agosta M, Bencardino D, Argentieri M, Pansani L, Sisto A, Ciofi Degli Atti ML, et al. Prevalence and molecular typing of carbapenemase-producing *Enterobacterales* among newborn patients in Italy. Antibiotics (Basel). 2022;11(4). Epub 2022/04/24. doi: 10.3390/antibiotics11040431. PubMed PMID: 35453183; PubMed Central PMCID: PMCPMC9032973.

8. Berglund B, Hoang NTB, Lundberg L, Le NK, Tärnberg M, Nilsson M, et al. Clonal spread of carbapenem-resistant *Klebsiella pneumoniae* among patients at admission and discharge at a Vietnamese neonatal intensive care unit. Antimicrob Resist Infect Control. 2021;10(1):162. Epub 2021/11/22. doi: 10.1186/s13756-021-01033-3. PubMed PMID: 34801068; PubMed Central PMCID: PMCPMC8606094.

9. Kk S, Ekedahl E, Hoang NTB, Sewunet T, Berglund B, Lundberg L, et al. High diversity of *bla*_NDM-1_-encoding plasmids in *Klebsiella pneumoniae* isolated from neonates in a Vietnamese hospital. Int J Antimicrob Agents. 2022;59(2):106496. Epub 2021/12/19. doi: 10.1016/j.ijantimicag.2021.106496. PubMed PMID: 34921976.

10. Sands K, Carvalho MJ, Portal E, Thomson K, Dyer C, Akpulu C, et al. Characterization of antimicrobial-resistant Gram-negative bacteria that cause neonatal sepsis in seven low- and middle-income countries. Nat Microbiol. 2021;6(4):512-23. Epub 2021/03/31. doi: 10.1038/s41564-021-00870-7. PubMed PMID: 33782558; PubMed Central PMCID: PMCPMC8007471.

11. Yin L, He L, Miao J, Yang W, Wang X, Ma J, et al. Actively surveillance and appropriate patients placements' contact isolation dramatically decreased carbapenem-resistant *Enterobacteriaceae* infection and colonization in pediatric patients in China. J Hosp Infect. 2020. Epub 2020/04/04. doi: 10.1016/j.jhin.2020.03.031. PubMed PMID: 32243954.

12. Yin L, He L, Miao J, Yang W, Wang X, Ma J, et al. Carbapenem-resistant Enterobacterales colonization and subsequent infection in a neonatal intensive care unit in Shanghai, China. Infect Prev Pract. 2021;3(3):100147. Epub 2021/10/15. doi: 10.1016/j.infpip.2021.100147. PubMed PMID: 34647006; PubMed Central PMCID: PMCPMC8498732.

13. Zhou J, Yang J, Hu F, Gao K, Sun J, Yang J. Clinical and molecular epidemiologic characteristics of ceftazidime/avibactam-resistant carbapenem-resistant *Klebsiella pneumoniae* in a neonatal intensive care unit in China. Infect Drug Resist. 2020;13:2571-8. Epub 2020/08/18. doi: 10.2147/idr.S256922. PubMed PMID: 32801794; PubMed Central PMCID: PMCPMC7394509.

14. Liu Y, Li XY, Wan LG, Jiang WY, Yang JH, Li FQ. Acquisition of carbapenem resistance in multiresistant *Klebsiella pneumoniae* isolates of sequence type 11 at a university hospital in China. Diagn Microbiol Infect Dis. 2013;76(2):241-3. Epub 2013/03/23. doi: 10.1016/j.diagmicrobio.2013.02.002. PubMed PMID: 23518183.

15. Cienfuegos-Gallet AV, Zhou Y, Ai W, Kreiswirth BN, Yu F, Chen L. Multicenter genomic analysis of carbapenem-resistant *Klebsiella pneumoniae* from bacteremia in China. Microbiol Spectr. 2022;10(2):e0229021. Epub 2022/03/02. doi: 10.1128/spectrum.02290-21. PubMed PMID: 35230130; PubMed Central PMCID: PMCPMC9045280.

16. Yu J, Tan K, Rong Z, Wang Y, Chen Z, Zhu X, et al. Nosocomial outbreak of KPC-2- and NDM-1-producing *Klebsiella pneumoniae* in a neonatal ward: a retrospective study. BMC Infect Dis. 2016;16(1):563. doi: 10.1186/s12879-016-1870-y. PubMed PMID: 27733128; PubMed Central PMCID: PMCPMC5062924.

17. Kong Z, Liu X, Li C, Cheng S, Xu F, Gu B. Clinical molecular epidemiology of carbapenem-resistant *Klebsiella pneumoniae* among pediatric patients in Jiangsu Province, China. Infect Drug Resist. 2020;13:4627-35. Epub 2020/12/31. doi: 10.2147/idr.S293206. PubMed PMID: 33376368; PubMed Central PMCID: PMCPMC7764961.

18. Liu J, Yu J, Chen F, Yu J, Simner P, Tamma P, et al. Emergence and establishment of KPC-2-producing ST11 Klebsiella pneumoniae in a general hospital in Shanghai, China. Eur J Clin Microbiol Infect Dis. 2018;37(2):293-9. doi: 10.1007/s10096-017-3131-4. PubMed PMID: 29282569; PubMed Central PMCID: PMCPMC5780533.

19. Patil S, Chen H, Guo C, Zhang X, Ren PG, Francisco NM, et al. Emergence of *Klebsiella pneumoniae* ST307 co-producing CTX-M with SHV and KPC from paediatric patients at Shenzhen Children's Hospital, China. Infect Drug Resist. 2021;14:3581-8. Epub 2021/09/14. doi: 10.2147/idr.S324018. PubMed PMID: 34511949; PubMed Central PMCID: PMCPMC8422287.

20. Jin C, Shi R, Jiang X, Zhou F, Qiang J, An C. Epidemic characteristics of carbapenem-resistant *Klebsiella pneumoniae* in the pediatric intensive care unit of Yanbian University Hospital, China. Infect Drug Resist. 2020;13:1439-46. Epub 2020/06/18. doi: 10.2147/idr.S245397. PubMed PMID: 32547112; PubMed Central PMCID: PMCPMC7244351.

21. Pei N, Li Y, Liu C, Jian Z, Liang T, Zhong Y, et al. Large-scale genomic epidemiology of *Klebsiella pneumoniae* identified clone divergence with hypervirulent plus antimicrobial-resistant characteristics causing within-ward strain transmissions. Microbiol Spectr. 2022;10(2):e0269821. Epub 2022/04/14. doi: 10.1128/spectrum.02698-21. PubMed PMID: 35416698; PubMed Central PMCID: PMCPMC9045374.

22. Li P, Wang M, Li X, Hu F, Yang M, Xie Y, et al. ST37 *Klebsiella pneumoniae*: development of carbapenem resistance in vivo during antimicrobial therapy in neonates. Future Microbiol. 2017;12:891-904. doi: 10.2217/fmb-2016-0165. PubMed PMID: 28699768.

23. Chen CM, Wang M, Li XP, Li PL, Tian JJ, Zhang K, et al. Homology analysis between clinically isolated extraintestinal and enteral *Klebsiella pneumoniae* among neonates. BMC Microbiol. 2021;21(1):25. Epub 2021/01/13. doi: 10.1186/s12866-020-02073-2. PubMed PMID: 33430787; PubMed Central PMCID: PMCPMC7802202.

24. Yan J, Dong C, Shao C, Yong W, Yun L. Molecular epidemiology of clonally related metallo-β-lactamase-producing *Klebsiella pneumoniae* isolated from newborns in a hospital in Shandong, China. Jundishapur Journal of Microbiology. 2017;In Press(In Press).

25. Rada AM, De La Cadena E, Agudelo C, Capataz C, Orozco N, Pallares C, et al. Dynamics of *bla*_KPC-2_ dissemination from non-CG258 *Klebsiella pneumoniae* to other *Enterobacterales* via IncN plasmids in an area of high endemicity. Antimicrob Agents Chemother. 2020;64(12). Epub 2020/09/23. doi: 10.1128/aac.01743-20. PubMed PMID: 32958711; PubMed Central PMCID: PMCPMC7674068.

26. Gona F, Bongiorno D, Aprile A, Corazza E, Pasqua B, Scuderi MG, et al. Emergence of two novel sequence types (3366 and 3367) NDM-1- and OXA-48-co-producing *K. pneumoniae* in Italy. Eur J Clin Microbiol Infect Dis. 2019;38(9):1687-91. Epub 2019/06/06. doi: 10.1007/s10096-019-03597-w. PubMed PMID: 31165962.

27. Yu J, Wang Y, Chen Z, Zhu X, Tian L, Li L, et al. Outbreak of nosocomial NDM-1-producing *Klebsiella pneumoniae* ST1419 in a neonatal unit. J Glob Antimicrob Resist. 2017;8:135-9. Epub 2017/01/23. doi: 10.1016/j.jgar.2016.10.014. PubMed PMID: 28109845.

28. Yin D, Dong D, Li K, Zhang L, Liang J, Yang Y, et al. Clonal dissemination of OXA-232 carbapenemase-producing *Klebsiella pneumoniae* in neonates. Antimicrob Agents Chemother. 2017;61(8). Epub 2017/05/24. doi: 10.1128/aac.00385-17. PubMed PMID: 28533245; PubMed Central PMCID: PMCPMC5527636.

29. Jánvári L, Damjanova I, Lázár A, Rácz K, Kocsis B, Urbán E, et al. Emergence of OXA-162-producing *Klebsiella pneumoniae* in Hungary. Scand J Infect Dis. 2014;46(4):320-4. Epub 2014/02/21. doi: 10.3109/00365548.2013.879993. PubMed PMID: 24552581.

30. Stoesser N, Giess A, Batty EM, Sheppard AE, Walker AS, Wilson DJ, et al. Genome sequencing of an extended series of NDM-producing *Klebsiella pneumoniae* isolates from neonatal infections in a Nepali hospital characterizes the extent of community- versus hospital-associated transmission in an endemic setting. Antimicrob Agents Chemother. 2014;58(12):7347-57. Epub 2014/10/01. doi: 10.1128/AAC.03900-14. PubMed PMID: 25267672; PubMed Central PMCID: PMCPMC4249533.

31. Ahmad N, Ali SM, Khan AU. Molecular characterization of novel sequence type of carbapenem-resistant New Delhi metallo-β-lactamase-1-producing *Klebsiella pneumoniae* in the neonatal intensive care unit of an Indian hospital. Int J Antimicrob Agents. 2019;53(4):525-9. Epub 2018/12/24. doi: 10.1016/j.ijantimicag.2018.12.005. PubMed PMID: 30578964.

32. Naha S, Sands K, Mukherjee S, Saha B, Dutta S, Basu S. OXA-181-like carbapenemases in *Klebsiella pneumoniae* ST14, ST15, ST23, ST48, and ST231 from septicemic neonates: coexistence with NDM-5, resistome, transmissibility, and genome diversity. mSphere. 2021;6(1). Epub 2021/01/15. doi: 10.1128/mSphere.01156-20. PubMed PMID: 33441403; PubMed Central PMCID: PMCPMC7845606.

33. Heinz E, Ejaz H, Bartholdson Scott J, Wang N, Gujaran S, Pickard D, et al. Resistance mechanisms and population structure of highly drug resistant *Klebsiella* in Pakistan during the introduction of the carbapenemase NDM-1. Sci Rep. 2019;9(1):2392. Epub 2019/02/23. doi: 10.1038/s41598-019-38943-7. PubMed PMID: 30787414; PubMed Central PMCID: PMCPMC6382945.

34. Yin D, Zhang L, Wang A, He L, Cao Y, Hu F, et al. Clinical and molecular epidemiologic characteristics of carbapenem-resistant *Klebsiella pneumoniae* infection/colonization among neonates in China. J Hosp Infect. 2018;100(1):21-8. Epub 2018/05/16. doi: 10.1016/j.jhin.2018.05.005. PubMed PMID: 29763630.

35. Principe L, Meroni E, Conte V, Mauri C, Di Pilato V, Giani T, et al. Mother-to-child transmission of KPC-producing *Klebsiella pneumoniae*: potential relevance of a low microbial urinary load for screening purposes. J Hosp Infect. 2018;98(3):314-6. Epub 2017/10/19. doi: 10.1016/j.jhin.2017.10.008. PubMed PMID: 29042234.

36. Berglund B, Hoang NTB, Tärnberg M, Le NK, Welander J, Nilsson M, et al. Colistin- and carbapenem-resistant *Klebsiella pneumoniae* carrying *mcr-1* and *bla*_OXA-48_ isolated at a paediatric hospital in Vietnam. J Antimicrob Chemother. 2018;73(4):1100-2. Epub 2017/12/19. doi: 10.1093/jac/dkx491. PubMed PMID: 29253209.

37. Wang S, Zhao J, Liu N, Yang F, Zhong Y, Gu X, et al. IMP-38-producing high-risk sequence type 307 *Klebsiella pneumoniae* strains from a neonatal unit in China. mSphere. 2020;5(4). Epub 2020/07/03. doi: 10.1128/mSphere.00407-20. PubMed PMID: 32611699; PubMed Central PMCID: PMCPMC7333572.

38. Geraci DM, Bonura C, Giuffrè M, Saporito L, Graziano G, Aleo A, et al. Is the monoclonal spread of the ST258, KPC-3-producing clone being replaced in southern Italy by the dissemination of multiple clones of carbapenem-nonsusceptible, KPC-3-producing *Klebsiella pneumoniae*? Clin Microbiol Infect. 2015;21(3):e15-7. Epub 2015/02/07. doi: 10.1016/j.cmi.2014.08.022. PubMed PMID: 25658574.

39. Maida CM, Bonura C, Geraci DM, Graziano G, Carattoli A, Rizzo A, et al. Outbreak of ST395 KPC-producing *Klebsiella pneumoniae* in a neonatal intensive care unit in Palermo, Italy. Infect Control Hosp Epidemiol. 2018;39(4):496-8. Epub 2018/02/16. doi: 10.1017/ice.2017.267. PubMed PMID: 29444730.

40. Kopotsa K, Mbelle NM, Osei Sekyere J. Epigenomics, genomics, resistome, mobilome, virulome and evolutionary phylogenomics of carbapenem-resistant *Klebsiella pneumoniae* clinical strains. Microb Genom. 2020;6(12). Epub 2020/11/11. doi: 10.1099/mgen.0.000474. PubMed PMID: 33170117; PubMed Central PMCID: PMCPMC8116673.

41. Adler A, Solter E, Masarwa S, Miller-Roll T, Abu-Libdeh B, Khammash H, et al. Epidemiological and microbiological characteristics of an outbreak caused by OXA-48-producing *Enterobacteriaceae* in a neonatal intensive care unit in Jerusalem, Israel. J Clin Microbiol. 2013;51(9):2926-30. Epub 2013/06/28. doi: 10.1128/jcm.01049-13. PubMed PMID: 23804390; PubMed Central PMCID: PMCPMC3754643.

42. Dubodelov DV, Lubasovskaya LA, Shubina ES, Mukosey IS, Korostin DO, Kochetkova TO, et al. [Genetic determinants of resistance of hospital-associated strains of *Klebsiella pneumoniae* to β-lactam antibiotics isolated in neonates]. Genetika. 2016;52(9):1097-102. PubMed PMID: 29369564.

43. Mairi A, Touati A, Ait Bessai S, Boutabtoub Y, Khelifi F, Sotto A, et al. Carbapenemase-producing *Enterobacteriaceae* among pregnant women and newborns in Algeria: Prevalence, molecular characterization, maternal-neonatal transmission, and risk factors for carriage. Am J Infect Control. 2019;47(1):105-8. Epub 2018/09/18. doi: 10.1016/j.ajic.2018.07.009. PubMed PMID: 30220617.

44. Escobar Pérez JA, Olarte Escobar NM, Castro-Cardozo B, Valderrama Márquez IA, Garzón Aguilar MI, Martinez de la Barrera L, et al. Outbreak of NDM-1-producing *Klebsiella pneumoniae* in a neonatal unit in Colombia. Antimicrob Agents Chemother. 2013;57(4):1957-60. Epub 2013/01/30. doi: 10.1128/aac.01447-12. PubMed PMID: 23357776; PubMed Central PMCID: PMCPMC3623329.

45. Huang X, Cheng X, Sun P, Tang C, Ni F, Liu G. Characteristics of NDM-1-producing *Klebsiella pneumoniae* ST234 and ST1412 isolates spread in a neonatal unit. BMC Microbiol. 2018;18(1):186. Epub 2018/11/16. doi: 10.1186/s12866-018-1334-1. PubMed PMID: 30428842; PubMed Central PMCID: PMCPMC6234558.

46. Nagaraj G, Shamanna V, Govindan V, Rose S, Sravani D, Akshata KP, et al. High-resolution genomic profiling of carbapenem-resistant *Klebsiella pneumoniae* isolates: a multicentric retrospective Indian study. Clin Infect Dis. 2021;73(Suppl_4):S300-s7. Epub 2021/12/02. doi: 10.1093/cid/ciab767. PubMed PMID: 34850832; PubMed Central PMCID: PMCPMC8634558.

47. Naha S, Sands K, Mukherjee S, Roy C, Rameez MJ, Saha B, et al. KPC-2-producing *Klebsiella pneumoniae* ST147 in a neonatal unit: Clonal isolates with differences in colistin susceptibility attributed to AcrAB-TolC pump. Int J Antimicrob Agents. 2020;55(3):105903. Epub 2020/01/20. doi: 10.1016/j.ijantimicag.2020.105903. PubMed PMID: 31954832.

48. Mukherjee S, Bhattacharjee A, Naha S, Majumdar T, Debbarma SK, Kaur H, et al. Molecular characterization of NDM-1-producing *Klebsiella pneumoniae* ST29, ST347, ST1224, and ST2558 causing sepsis in neonates in a tertiary care hospital of North-East India. Infect Genet Evol. 2019;69:166-75. Epub 2019/01/25. doi: 10.1016/j.meegid.2019.01.024. PubMed PMID: 30677535.

49. Chen D, Hu X, Chen F, Li H, Wang D, Li X, et al. Co-outbreak of multidrug resistance and a novel ST3006 *Klebsiella pneumoniae* in a neonatal intensive care unit: A retrospective study. Medicine (Baltimore). 2019;98(4):e14285. Epub 2019/01/27. doi: 10.1097/md.0000000000014285. PubMed PMID: 30681632; PubMed Central PMCID: PMCPMC6358387.

50. Qiao F, Wei L, Feng Y, Ran S, Zheng L, Zhang Y, et al. Handwashing sink contamination and carbapenem-resistant *Klebsiella* infection in the intensive care unit: a prospective multicenter study. Clin Infect Dis. 2020;71(Suppl 4):S379-s85. Epub 2020/12/29. doi: 10.1093/cid/ciaa1515. PubMed PMID: 33367578.

51. Wei L, Feng Y, Wen H, Ya H, Qiao F, Zong Z. NDM-5-producing carbapenem-resistant *Klebsiella pneumoniae* of sequence type 789 emerged as a threat for neonates: a multicentre, genome-based study. Int J Antimicrob Agents. 2022;59(2):106508. Epub 2021/12/28. doi: 10.1016/j.ijantimicag.2021.106508. PubMed PMID: 34958865.

52. Zhu J, Sun L, Ding B, Yang Y, Xu X, Liu W, et al. Outbreak of NDM-1-producing *Klebsiella pneumoniae* ST76 and ST37 isolates in neonates. Eur J Clin Microbiol Infect Dis. 2016;35(4):611-8. doi: 10.1007/s10096-016-2578-z. PubMed PMID: 26803822.

53. Esposito EP, Gaiarsa S, Del Franco M, Crivaro V, Bernardo M, Cuccurullo S, et al. A novel IncA/C1 group conjugative plasmid, encoding VIM-1 metallo-beta-lactamase, mediates the acquisition of carbapenem resistance in ST104 *Klebsiella pneumoniae* Isolates from neonates in the intensive care unit of V. Monaldi Hospital in Naples. Front Microbiol. 2017;8:2135. Epub 2017/11/23. doi: 10.3389/fmicb.2017.02135. PubMed PMID: 29163422; PubMed Central PMCID: PMCPMC5675864.

54. Zheng R, Zhang Q, Guo Y, Feng Y, Liu L, Zhang A, et al. Outbreak of plasmid-mediated NDM-1-producing *Klebsiella pneumoniae* ST105 among neonatal patients in Yunnan, China. Ann Clin Microbiol Antimicrob. 2016;15:10. Epub 2016/02/21. doi: 10.1186/s12941-016-0124-6. PubMed PMID: 26896089; PubMed Central PMCID: PMCPMC4761218.

55. Sharma S, Banerjee T, Kumar A, Yadav G, Basu S. Extensive outbreak of colistin resistant, carbapenemase (*bla*_OXA-48_, *bla*_NDM_) producing *Klebsiella pneumoniae* in a large tertiary care hospital, India. Antimicrob Resist Infect Control. 2022;11(1):1. Epub 2022/01/08. doi: 10.1186/s13756-021-01048-w. PubMed PMID: 34991724; PubMed Central PMCID: PMCPMC8740481.

56. Banerjee T, Wangkheimayum J, Sharma S, Kumar A, Bhattacharjee A. Extensively drug-resistant hypervirulent *Klebsiella pneumoniae* from a series of neonatal sepsis in a tertiary care hospital, India. Front Med (Lausanne). 2021;8:645955. Epub 2021/03/26. doi: 10.3389/fmed.2021.645955. PubMed PMID: 33763435; PubMed Central PMCID: PMCPMC7982647.

57. Falco A, Ramos Y, Franco E, Guzmán A, Takiff H. A cluster of KPC-2 and VIM-2-producing *Klebsiella pneumoniae* ST833 isolates from the pediatric service of a Venezuelan Hospital. BMC Infect Dis. 2016;16(1):595. Epub 2016/10/25. doi: 10.1186/s12879-016-1927-y. PubMed PMID: 27770796; PubMed Central PMCID: PMCPMC5075218.

58. Kong Z, Cai R, Cheng C, Zhang C, Kang H, Ma P, et al. First reported nosocomial outbreak of NDM-5-producing *Klebsiella pneumoniae* in a neonatal unit in China. Infect Drug Resist. 2019;12:3557-66. Epub 2019/12/10. doi: 10.2147/idr.S218945. PubMed PMID: 31814744; PubMed Central PMCID: PMCPMC6863125.

59. Giuffrè M, Bonura C, Geraci DM, Saporito L, Catalano R, Di Noto S, et al. Successful control of an outbreak of colonization by *Klebsiella pneumoniae* carbapenemase-producing *K. pneumoniae* sequence type 258 in a neonatal intensive care unit, Italy. J Hosp Infect. 2013;85(3):233-6. Epub 2013/10/01. doi: 10.1016/j.jhin.2013.08.004. PubMed PMID: 24074641.

60. Kiaei S, Moradi M, Hosseini-Nave H, Ziasistani M, Kalantar-Neyestanaki D. Endemic dissemination of different sequence types of carbapenem-resistant *Klebsiella pneumoniae* strains harboring *bla*_NDM_ and *16S rRNA* methylase genes in Kerman hospitals, Iran, from 2015 to 2017. Infect Drug Resist. 2019;12:45-54. Epub 2019/01/08. doi: 10.2147/idr.S186994. PubMed PMID: 30613156; PubMed Central PMCID: PMCPMC6306073.

61. Bai Y, Shao C, Hao Y, Wang Y, Jin Y. Using whole genome sequencing to trace, control and characterize a hospital infection of IMP-4-producing *Klebsiella pneumoniae* ST2253 in a neonatal unit in a tertiary hospital, China. Front Public Health. 2021;9:755252. Epub 2022/01/04. doi: 10.3389/fpubh.2021.755252. PubMed PMID: 34976919; PubMed Central PMCID: PMCPMC8715938.

62. Brinkac LM, White R, D'Souza R, Nguyen K, Obaro SK, Fouts DE. Emergence of New Delhi metallo-β-Lactamase (NDM-5) in *Klebsiella quasipneumoniae* from neonates in a Nigerian hospital. mSphere. 2019;4(2). Epub 2019/03/15. doi: 10.1128/mSphere.00685-18. PubMed PMID: 30867330; PubMed Central PMCID: PMCPMC6416368.

63. Farzana R, Jones LS, Rahman MA, Andrey DO, Sands K, Portal E, et al. Outbreak of hypervirulent multidrug-resistant *Klebsiella variicola* causing high mortality in neonates in Bangladesh. Clin Infect Dis. 2019;68(7):1225-7. Epub 2018/09/12. doi: 10.1093/cid/ciy778. PubMed PMID: 30204843.

64. Mukherjee S, Naha S, Bhadury P, Saha B, Dutta M, Dutta S, et al. Emergence of OXA-232-producing hypervirulent *Klebsiella pneumoniae* ST23 causing neonatal sepsis. J Antimicrob Chemother. 2020;75(7):2004-6. Epub 2020/03/11. doi: 10.1093/jac/dkaa080. PubMed PMID: 32155265.

65. Bonfanti P, Bellù R, Principe L, Caramma I, Condò M, Giani T, et al. Mother-to-child transmission of KPC carbapenemase-producing *Klebsiella pneumoniae* at birth. Pediatr Infect Dis J. 2017;36(2):228-9. Epub 2016/11/16. doi: 10.1097/inf.0000000000001403. PubMed PMID: 27846056.

66. Zhang Y, Zeng J, Liu W, Zhao F, Hu Z, Zhao C, et al. Emergence of a hypervirulent carbapenem-resistant *Klebsiella pneumoniae* isolate from clinical infections in China. J Infect. 2015;71(5):553-60. Epub 2015/08/26. doi: 10.1016/j.jinf.2015.07.010. PubMed PMID: 26304687.

67. Ramsamy Y, Mlisana KP, Allam M, Amoako DG, Abia ALK, Ismail A, et al. Genomic analysis of carbapenemase-producing extensively drug-resistant *Klebsiella pneumoniae* isolates reveals the horizontal spread of p18-43_01 plasmid encoding *bla*_NDM-1_ in South Africa. Microorganisms. 2020;8(1). Epub 2020/01/23. doi: 10.3390/microorganisms8010137. PubMed PMID: 31963608; PubMed Central PMCID: PMCPMC7023316.

68. Bhattacharjee B, Bardhan T, Chakraborty M, Basu M. Resistance profiles and resistome mapping of multidrug resistant carbapenem-hydrolyzing *Klebsiella pneumoniae* strains isolated from the nares of preterm neonates. Int J Antimicrob Agents. 2019;53(4):535-7. Epub 2018/12/21. doi: 10.1016/j.ijantimicag.2018.12.002. PubMed PMID: 30572009.

69. Li J, Hu X, Yang L, Lin Y, Liu Y, Li P, et al. New Delhi metallo-β-Lactamase 1-producing *Klebsiella pneumoniae* ST719 isolated from a neonate in China. Microb Drug Resist. 2020;26(5):492-6. Epub 2019/11/16. doi: 10.1089/mdr.2019.0058. PubMed PMID: 31730396.

70. Abe R, Oyama F, Akeda Y, Nozaki M, Hatachi T, Okamoto Y, et al. Hospital-wide outbreaks of carbapenem-resistant *Enterobacteriaceae* horizontally spread through a clonal plasmid harbouring *bla*_IMP-1_ in children's hospitals in Japan. J Antimicrob Chemother. 2021;76(12):3314-7. Epub 2021/09/04. doi: 10.1093/jac/dkab303. PubMed PMID: 34477841.
