# Supplementary material for: Prevalence and clonal diversity of carbapenem-resistant Klebsiella pneumoniae causing neonatal infections: A systematic review of 128 articles across 30 countries
Source: PLoS Med. 2023 Jun 20;20(6):e1004233. doi: 10.1371/journal.pmed.1004233 (PMC10281588; doi:10.1371/journal.pmed.1004233)
Supplement: S6 Table — (DOCX) [file pmed.1004233.s009.docx]

S6 Table. Profiles of new STs compared with their nearest neighbour

| ST | *gapA* | *infB* | *mdh* | *pgi* | *phoE* | *rpoB* | *tonB* |
| --- | --- | --- | --- | --- | --- | --- | --- |
| ST11 | 3 | 3 | 1 | 1 | 1 | 1 | 4 |
| ST11V1 | 3 | 3 | 1 | 1 | 1 | 1 | **~4 (1)** |
| ST15 | 1 | 1 | 1 | 1 | 1 | 1 | 1 |
| ST15V1 | 1 | 1 | 1 | 1 | 1 | 1 | **~1 (1)** |
| ST15V2 | **~1 (1)** | 1 | 1 | 1 | 1 | 1 | 1 |
| ST15V3 | 1 | 1 | 1 | 1 | **~1 (1)** | 1 | 1 |
| ST15V4 | 1 | 1 | 1 | 1 | 1 | 1 | **~1 (2)** |
| ST3348 | 178 | 3 | 1 | 1 | 1 | 1 | 4 |
| ST3348V1 | **-** | 3 | 1 | 1 | **~1 (1)** | 1 | 4 |
| ST464 | 2 | 5 | 2 | 1 | 26 | 1 | 2 |
| ST464V1 | **-** | 5 | 2 | 1 | 26 | 1 | 2 |
| ST915 | 2 | 10 | 13 | 1 | 12 | 1 | 186 |
| ST915V1 | 2 | 10 | 13 | 1 | 12 | 1 | **18** |

New, undefined ST are assigned here according to their nearest STs with a V (representing variant).

Tilde (~) denotes a novel allele, the designation of which has not been assigned yet; the number in the brackets denotes the number of nucleotide mismatches compared to its nearest neighbour.

Hyphen (-) represents a missing allele resulted from truncation.
