# Supplementary material for: Prevalence and clonal diversity of carbapenem-resistant Klebsiella pneumoniae causing neonatal infections: A systematic review of 128 articles across 30 countries
Source: PLoS Med. 2023 Jun 20;20(6):e1004233. doi: 10.1371/journal.pmed.1004233 (PMC10281588; doi:10.1371/journal.pmed.1004233)
Supplement: S7 Table — (DOCX) [file pmed.1004233.s010.docx]

S7 Table. Carbapenemases in neonatal CRKP strains with information for STs.

| Ambler class | no. | ST | Country or Region | References |
| --- | --- | --- | --- | --- |
| A | 257 |  |  |  |
| KPC-2 | 181 | 11, 17, 36, 101, 140, 147, 307, 334, 502, 2053 | Bangladesh, China, Colombia, India, Vietnam | [1-16] |
| KPC-3 | 25 | 258, 307, 323, 395, 512 | Italy | [17-21] |
| KPC-nd | 49 | 11, 17, 101, 307, 395, 512 | China, Colombia, India, Italy, Portugal | [22-27] |
| SME-1 | 1 | 778 | China | [9] |
| SME-nd | 1 | - | China | [28] |
| B | 990 (824+166) | | | |
| MBL-nd | 25 | - | India, Pakistan | [29, 30] |
| IMP-1 | 1 | 915V1 | Japan | [31] |
| IMP-38 | 24 | 307 | China | [9, 12, 32, 33] |
| IMP-4 | 55 (52+3) | 34, 54, 705, 736, 1027, 2253, 29, 54, 628 | China | [5, 12, 13, 34-41] |
| IMP-8 | 13 | 290 | China | [13, 36, 38, 41] |
| IMP-nd | 3 | 307 | China | [23] |
| NDM-1 | 530 (441+89) | 11, 14, 15, 16, 17, 20, 22, 25, 29, 35, 37, 39, 45, 54, 70, 76, 101, 105, 147, 152, 188, 234, 278, 347, 377, 395, 433, 442, 477, 485, 611, 657, 719, 771, 846, 888, 967, 995, 1043, 1224, 1412, 1419, 2236, 2534, 2558, 2735, 2736, 2738, 2823, 3003, 3228, 3344, 3350, 3366, 3367, 3444, 4410, 4854, 5014, 5015, 5016, 5017, 5235, 42, 48, 244, 307, 323, 413, 571, 726, 736, 997, 1310, 1317, 1322, 4494, 4511, 4979, 4981, 11V1 | Bangladesh, China, Colombia, India, Italy, Nepal, Nigeria, Pakistan, South Africa, Vietnam, Kenya | [2, 4, 5, 9, 10, 12-16, 28, 33, 36, 38, 39, 42-70] |
| NDM-4 | 56 | 16, 11 | India, Pakistan, Vietnam | [15, 56, 68] |
| NDM-5 | 173 (119+54) | 11, 14, 15, 337, 476, 789, 873, 17, 24, 35, 37, 231, 340, 394, 2407 | Bangladesh, China, India, Nigeria, Vietnam, Kenya, | [3, 4, 14, 15, 37, 39, 56, 68, 70-73] |
| NDM-6 | 1 | 64 | China | [9] |
| NDM-7 | 25 (10+15) | 464, 2355, 11, 22, 36, 39, 711, 1031, 1998 | Bangladesh, Nigeria, India, Kenya, Pakistan | [4] |
| NDM-nd | 60 (56+4) | 17, 35, 43, 45, 268, 395, 1412, 11, 16 | Bangladesh, China, India, Iran, Italy, Pakistan, South Africa, United Kingdom | [23, 24, 74-77] |
| VIM-1 | 20 | 104 | Italy | [78] |
| VIM-12 | 1 | 17 | Greece |  |
| VIM-nd | 3 | - | Italy, Portugal, South Africa | [25, 75, 79] |
| B+B | 17 (14+3) |  |  |  |
| IMP-38+NDM-1 | 5 | - | China | [2] |
| IMP-4+NDM-1 | 7 | 54, 105, 29, 54, 628 | China | [36, 61] |
| NDM-1+VIM-nd | 1 | - | China | [28] |
| NDM-1+NDM-7 | 1 | 11 | Pakistan |  |
| NDM-4+NDM-nd | 1 | 11 | Pakistan |  |
| NDM-7+NDM-nd | 1 | 3348V1 | Pakistan |  |
| NDM-nd+VIM-nd | 1 | - | Egypt | [80] |
| D | 135 (131+4) | | | |
| OXA-162 | 1 | 15 | Hungary | [81] |
| OXA-181 | 36 | 14, 15, 17, 25, 48, 147 | Ghana, India, Pakistan, Vietnam | [4, 15, 82-84] |
| OXA-232 | 13 | 14, 15, 23, 231 | Bangladesh, China, India | [4, 33, 83, 85, 86] |
| OXA-48 | 84 (81+3) | 11, 13, 39, 45, 307, 395, 1878, 5235, 147 | Algeria, Egypt, France, India, Israel, Italy, Morocco, Portugal, Russia, South Africa, Vietnam, Pakistan | [24, 25, 87-97] |
| OXA-48-nd | 1 | 38 | Kenya |  |
| A+B | 50 |  |  |  |
| KPC-2+VIM-2 | 14 | 833 | Venezuela | [98] |
| KPC-nd+NDM-1 | 3 | - | China | [28] |
| KPC-nd+NDM-nd | 16 | 17, 395, 462, 3350 | China, Italy | [23, 24] |
| KPC-nd+VIM-nd | 19 | - | Tunisia | [99] |
| B+D | 120 (89+31) | | | |
| NDM-1+OXA-181 | 42 (20+22) | 15, 25, 413, 15V3 | India, Pakistan, Kenya | [4] |
| NDM-1+OXA-23 | 23 | 17, 278, 846 | China | [16] |
| NDM-1+OXA-232 | 9 (2+7) | 14 | Bangladesh | [4] |
| NDM-1+OXA-48 | 21 | 11, 16,39, 101, 485, 3366, 3367, 5235 | China, India, Italy, South Africa | [9, 50, 51, 56, 90, 100] |
| NDM-4+OXA-48 | 1 | 16 | India | [56] |
| NDM-5+OXA-181 | 3 | 14 | India | [83] |
| NDM-5+OXA-232 | 2 | 14, 437 | India | [82, 83] |
| NDM-5+OXA-48 | 1 | 873 | India | [56] |
| NDM-nd+OXA-48 | 16 | 5235 | Egypt, India, South Africa | [87, 91, 92] |
| NDM-nd+OXA-181 | 2 | 15 | Pakistan |  |
| A+B+B | 22 |  |  |  |
| KPC-nd+NDM-nd+VIM-nd | 22 | - | Egypt | [80] |
| B+B+D | 1 |  |  |  |
| IMP-38+NDM-1+OXA-48 | 1 | 147 | China | [9] |
| Total | 1592 (1388+204) | | | |

-nd, not determined. MBL-nd, metallo-β-lactamases, but the exact types have not been determined.

References

1. Liu Y, Li XY, Wan LG, Jiang WY, Yang JH, Li FQ. Acquisition of carbapenem resistance in multiresistant *Klebsiella pneumoniae* isolates of sequence type 11 at a university hospital in China. Diagn Microbiol Infect Dis. 2013;76(2):241-3. Epub 2013/03/23. doi: 10.1016/j.diagmicrobio.2013.02.002. PubMed PMID: 23518183.

2. Yin L, He L, Miao J, Yang W, Wang X, Ma J, et al. Actively surveillance and appropriate patients placements' contact isolation dramatically decreased carbapenem-resistant Enterobacteriaceae infection and colonization in pediatric patients in China. J Hosp Infect. 2020. Epub 2020/04/04. doi: 10.1016/j.jhin.2020.03.031. PubMed PMID: 32243954.

3. Wang B, Pan F, Wang C, Zhao W, Sun Y, Zhang T, et al. Molecular epidemiology of carbapenem-resistant *Klebsiella pneumoniae* in a paediatric hospital in China. Int J Infect Dis. 2020;93:311-9. Epub 2020/02/19. doi: 10.1016/j.ijid.2020.02.009. PubMed PMID: 32068096.

4. Sands K, Carvalho MJ, Portal E, Thomson K, Dyer C, Akpulu C, et al. Characterization of antimicrobial-resistant Gram-negative bacteria that cause neonatal sepsis in seven low- and middle-income countries. Nat Microbiol. 2021;6(4):512-23. Epub 2021/03/31. doi: 10.1038/s41564-021-00870-7. PubMed PMID: 33782558; PubMed Central PMCID: PMCPMC8007471.

5. Kong Z, Liu X, Li C, Cheng S, Xu F, Gu B. Clinical molecular epidemiology of carbapenem-resistant *Klebsiella pneumoniae* among pediatric patients in Jiangsu Province, China. Infect Drug Resist. 2020;13:4627-35. Epub 2020/12/31. doi: 10.2147/idr.S293206. PubMed PMID: 33376368; PubMed Central PMCID: PMCPMC7764961.

6. Saavedra SY, Bernal JF, Montilla-Escudero E, Arévalo SA, Prada DA, Valencia MF, et al. Complexity of genomic epidemiology of carbapenem-resistant *Klebsiella pneumoniae* isolates in Colombia urges the reinforcement of whole genome sequencing-based surveillance programs. Clin Infect Dis. 2021;73(Suppl_4):S290-s9. Epub 2021/12/02. doi: 10.1093/cid/ciab777. PubMed PMID: 34850835; PubMed Central PMCID: PMCPMC8634422.

7. Rada AM, De La Cadena E, Agudelo C, Capataz C, Orozco N, Pallares C, et al. Dynamics of *bla*_KPC-2_ dissemination from non-CG258 *Klebsiella pneumoniae* to other *Enterobacterales* via IncN plasmids in an area of high endemicity. Antimicrob Agents Chemother. 2020;64(12). Epub 2020/09/23. doi: 10.1128/aac.01743-20. PubMed PMID: 32958711; PubMed Central PMCID: PMCPMC7674068.

8. Liu J, Yu J, Chen F, Yu J, Simner P, Tamma P, et al. Emergence and establishment of KPC-2-producing ST11 *Klebsiella pneumoniae* in a general hospital in Shanghai, China. Eur J Clin Microbiol Infect Dis. 2018;37(2):293-9. Epub 2017/12/29. doi: 10.1007/s10096-017-3131-4. PubMed PMID: 29282569; PubMed Central PMCID: PMCPMC5780533 ETHICAL APPROVAL: Ethical approval was not required. INFORMED CONSENT: No informed consent was required since this was a retrospective study.

9. Patil S, Chen H, Guo C, Zhang X, Ren PG, Francisco NM, et al. Emergence of *Klebsiella pneumoniae* ST307 co-producing CTX-M with SHV and KPC from paediatric patients at Shenzhen Children's Hospital, China. Infect Drug Resist. 2021;14:3581-8. Epub 2021/09/14. doi: 10.2147/idr.S324018. PubMed PMID: 34511949; PubMed Central PMCID: PMCPMC8422287.

10. Jin C, Shi R, Jiang X, Zhou F, Qiang J, An C. Epidemic characteristics of carbapenem-resistant *Klebsiella pneumoniae* in the pediatric intensive care unit of Yanbian University Hospital, China. Infect Drug Resist. 2020;13:1439-46. Epub 2020/06/18. doi: 10.2147/idr.S245397. PubMed PMID: 32547112; PubMed Central PMCID: PMCPMC7244351.

11. Naha S, Sands K, Mukherjee S, Roy C, Rameez MJ, Saha B, et al. KPC-2-producing *Klebsiella pneumoniae* ST147 in a neonatal unit: Clonal isolates with differences in colistin susceptibility attributed to AcrAB-TolC pump. Int J Antimicrob Agents. 2020;55(3):105903. Epub 2020/01/20. doi: 10.1016/j.ijantimicag.2020.105903. PubMed PMID: 31954832.

12. Pei N, Li Y, Liu C, Jian Z, Liang T, Zhong Y, et al. Large-scale genomic epidemiology of *Klebsiella pneumoniae* identified clone divergence with hypervirulent plus antimicrobial-resistant characteristics causing within-ward strain transmissions. Microbiol Spectr. 2022;10(2):e0269821. Epub 2022/04/14. doi: 10.1128/spectrum.02698-21. PubMed PMID: 35416698; PubMed Central PMCID: PMCPMC9045374.

13. Dong F, Lu J, Wang Y, Shi J, Zhen JH, Chu P, et al. A five-year surveillance of carbapenemase-producing *Klebsiella pneumoniae* in a pediatric hospital in China reveals increased predominance of NDM-1. Biomed Environ Sci. 2017;30(8):562-9. Epub 2017/08/16. doi: 10.3967/bes2017.075. PubMed PMID: 28807096.

14. Zhou J, Yang J, Hu F, Gao K, Sun J, Yang J. Clinical and molecular epidemiologic characteristics of ceftazidime/avibactam-resistant carbapenem-resistant *Klebsiella pneumoniae* in a neonatal intensive care unit in China. Infect Drug Resist. 2020;13:2571-8. Epub 2020/08/18. doi: 10.2147/idr.S256922. PubMed PMID: 32801794; PubMed Central PMCID: PMCPMC7394509.

15. Berglund B, Hoang NTB, Lundberg L, Le NK, Tarnberg M, Nilsson M, et al. Clonal spread of carbapenem-resistant *Klebsiella pneumoniae* among patients at admission and discharge at a Vietnamese neonatal intensive care unit. Antimicrob Resist Infect Control. 2021;10(1):162. doi: ARTN 162

10.1186/s13756-021-01033-3. PubMed PMID: WOS:000720713000001.

16. Yin L, He L, Miao J, Yang W, Wang X, Ma J, et al. Carbapenem-resistant *Enterobacterales* colonization and subsequent infection in a neonatal intensive care unit in Shanghai, China. Infect Prev Pract. 2021;3(3):100147. Epub 2021/10/15. doi: 10.1016/j.infpip.2021.100147. PubMed PMID: 34647006; PubMed Central PMCID: PMCPMC8498732.

17. Giuffrè M, Bonura C, Geraci DM, Saporito L, Catalano R, Di Noto S, et al. Successful control of an outbreak of colonization by *Klebsiella pneumoniae* carbapenemase-producing *K. pneumoniae* sequence type 258 in a neonatal intensive care unit, Italy. J Hosp Infect. 2013;85(3):233-6. Epub 2013/10/01. doi: 10.1016/j.jhin.2013.08.004. PubMed PMID: 24074641.

18. Principe L, Meroni E, Conte V, Mauri C, Di Pilato V, Giani T, et al. Mother-to-child transmission of KPC-producing *Klebsiella pneumoniae*: potential relevance of a low microbial urinary load for screening purposes. J Hosp Infect. 2018;98(3):314-6. Epub 2017/10/19. doi: 10.1016/j.jhin.2017.10.008. PubMed PMID: 29042234.

19. Geraci DM, Bonura C, Giuffrè M, Saporito L, Graziano G, Aleo A, et al. Is the monoclonal spread of the ST258, KPC-3-producing clone being replaced in southern Italy by the dissemination of multiple clones of carbapenem-nonsusceptible, KPC-3-producing *Klebsiella pneumoniae*? Clin Microbiol Infect. 2015;21(3):e15-7. Epub 2015/02/07. doi: 10.1016/j.cmi.2014.08.022. PubMed PMID: 25658574.

20. Maida CM, Bonura C, Geraci DM, Graziano G, Carattoli A, Rizzo A, et al. Outbreak of ST395 KPC-producing *Klebsiella pneumoniae* in a neonatal intensive care unit in Palermo, Italy. Infect Control Hosp Epidemiol. 2018;39(4):496-8. Epub 2018/02/16. doi: 10.1017/ice.2017.267. PubMed PMID: 29444730.

21. Bonfanti P, Bellù R, Principe L, Caramma I, Condò M, Giani T, et al. Mother-to-child transmission of KPC carbapenemase-producing *Klebsiella pneumoniae* at birth. Pediatr Infect Dis J. 2017;36(2):228-9. Epub 2016/11/16. doi: 10.1097/inf.0000000000001403. PubMed PMID: 27846056.

22. Gayathiri Govindaraju VA, TM Uma Mageswari, Balakrishnan Rajaiah, and Srinivas Ramakrishnan. Sepsis in neonates: Prevalence of micro-organisms and their susceptibility pattern in neonatal intensive care unit of a tertiary care hospital – a retrospective study. J Basic Clin Pharma. 2020;11:11‐6.

23. Cienfuegos-Gallet AV, Zhou Y, Ai W, Kreiswirth BN, Yu F, Chen L. Multicenter genomic analysis of carbapenem-resistant *Klebsiella pneumoniae* from bacteremia in China. Microbiol Spectr. 2022;10(2):e0229021. Epub 2022/03/02. doi: 10.1128/spectrum.02290-21. PubMed PMID: 35230130; PubMed Central PMCID: PMCPMC9045280.

24. Agosta M, Bencardino D, Argentieri M, Pansani L, Sisto A, Ciofi Degli Atti ML, et al. Prevalence and molecular typing of carbapenemase-producing *Enterobacterales* among newborn patients in Italy. Antibiotics (Basel). 2022;11(4). Epub 2022/04/24. doi: 10.3390/antibiotics11040431. PubMed PMID: 35453183; PubMed Central PMCID: PMCPMC9032973.

25. Almeida TL, Mendo T, Costa R, Novais C, Marçal M, Martins F, et al. Carbapenemase-producing *Enterobacteriaceae* (CPE) newborn colonization in a Portuguese neonatal intensive care unit (NICU): epidemiology and infection prevention and control measures. Infect Dis Rep. 2021;13(2):411-7. Epub 2021/06/03. doi: 10.3390/idr13020039. PubMed PMID: 34062713; PubMed Central PMCID: PMCPMC8162345.

26. Magda Sánchez DJM. Detección rápida de *Enterobacterias* productoras de carbapenemasas en hisopados rectales de pacientes neonatos colonizados. Infection. 2021;25(2):89-92.

27. Ma MS, Wang DH, Sun XJ, Li ZH, Wang C. [Risk factors for *Klebsiella pneumoniae* carbapenemase-producing *Klebsiella pneumoniae* colonization in neonates]. Zhongguo Dang Dai Er Ke Za Zhi. 2014;16(10):970-4. PubMed PMID: 25344173.

28. Yang Y, Liu J, Muhammad M, Liu H, Min Z, Lu J, et al. Factors behind the prevalence of carbapenem-resistant *Klebsiella pneumoniae* in pediatric wards. Medicine (Baltimore). 2021;100(36):e27186. Epub 2021/09/14. doi: 10.1097/md.0000000000027186. PubMed PMID: 34516520; PubMed Central PMCID: PMCPMC8428699.

29. Humayun A, Siddiqui FM, Akram N, Saleem S, Ali A, Iqbal T, et al. Incidence of metallo-beta-lactamase-producing *Klebsiella pneumoniae* isolates from hospital setting in Pakistan. Int Microbiol. 2018;21(1-2):73-8. Epub 2019/02/28. doi: 10.1007/s10123-018-0006-1. PubMed PMID: 30810920.

30. Gajul SV, Mohite ST, Mangalgi SS, Wavare SM, Kakade SV. *Klebsiella Pneumoniae* in septicemic neonates with special reference to extended spectrum β-lactamase, AmpC, metallo β-lactamase production and multiple drug resistance in tertiary care hospital. J Lab Physicians. 2015;7(1):32-7. Epub 2015/05/08. doi: 10.4103/0974-2727.151689. PubMed PMID: 25949057; PubMed Central PMCID: PMCPMC4411807.

31. Abe R, Oyama F, Akeda Y, Nozaki M, Hatachi T, Okamoto Y, et al. Hospital-wide outbreaks of carbapenem-resistant Enterobacteriaceae horizontally spread through a clonal plasmid harbouring *bla*_IMP-1_ in children's hospitals in Japan. J Antimicrob Chemother. 2021;76(12):3314-7. Epub 2021/09/04. doi: 10.1093/jac/dkab303. PubMed PMID: 34477841.

32. Wang S, Zhao J, Liu N, Yang F, Zhong Y, Gu X, et al. IMP-38-producing high-risk sequence type 307 *Klebsiella pneumoniae* strains from a neonatal unit in China. mSphere. 2020;5(4). Epub 2020/07/03. doi: 10.1128/mSphere.00407-20. PubMed PMID: 32611699; PubMed Central PMCID: PMCPMC7333572.

33. Yin D, Zhang L, Wang A, He L, Cao Y, Hu F, et al. Clinical and molecular epidemiologic characteristics of carbapenem-resistant *Klebsiella pneumoniae* infection/colonization among neonates in China. J Hosp Infect. 2018;100(1):21-8. Epub 2018/05/16. doi: 10.1016/j.jhin.2018.05.005. PubMed PMID: 29763630.

34. Chen S, Feng W, Chen J, Liao W, He N, Wang Q, et al. Spread of carbapenemase-producing enterobacteria in a southwest hospital in China. Ann Clin Microbiol Antimicrob. 2014;13:42. Epub 2014/08/13. doi: 10.1186/s12941-014-0042-4. PubMed PMID: 25113057; PubMed Central PMCID: PMCPMC4236511.

35. Bai Y, Shao C, Hao Y, Wang Y, Jin Y. Using whole genome sequencing to trace, control and characterize a hospital infection of IMP-4-producing *Klebsiella pneumoniae* ST2253 in a neonatal unit in a tertiary hospital, China. Front Public Health. 2021;9:755252. Epub 2022/01/04. doi: 10.3389/fpubh.2021.755252. PubMed PMID: 34976919; PubMed Central PMCID: PMCPMC8715938.

36. Jin Y, Song X, Liu Y, Wang Y, Zhang B, Fan H, et al. Characteristics of carbapenemase-producing *Klebsiella pneumoniae* as a cause of neonatal infection in Shandong, China. Exp Ther Med. 2017;13(3):1117-26. Epub 2017/04/30. doi: 10.3892/etm.2017.4070. PubMed PMID: 28450951; PubMed Central PMCID: PMCPMC5403258.

37. Qiao F, Wei L, Feng Y, Ran S, Zheng L, Zhang Y, et al. Handwashing sink contamination and carbapenem-resistant *Klebsiella* infection in the intensive care unit: a prospective multicenter study. Clin Infect Dis. 2020;71(Suppl 4):S379-s85. Epub 2020/12/29. doi: 10.1093/cid/ciaa1515. PubMed PMID: 33367578.

38. Yan J, Dong C, Shao C, Yong W, Yun L. Molecular epidemiology of clonally related metallo-β-lactamase-producing *Klebsiella pneumoniae* isolated from newborns in a hospital in Shandong, China. Jundishapur Journal of Microbiology. 2017;In Press(In Press).

39. Luo K, Tang J, Qu Y, Yang X, Zhang L, Chen Z, et al. Nosocomial infection by *Klebsiella pneumoniae* among neonates: a molecular epidemiological study. J Hosp Infect. 2021;108:174-80. Epub 2020/12/09. doi: 10.1016/j.jhin.2020.11.028. PubMed PMID: 33290814.

40. Yu F, Ying Q, Chen C, Li T, Ding B, Liu Y, et al. Outbreak of pulmonary infection caused by *Klebsiella pneumoniae* isolates harbouring *bla*_IMP-4_ and *bla*_DHA-1_ in a neonatal intensive care unit in China. J Med Microbiol. 2012;61(Pt 7):984-9. Epub 2012/04/03. doi: 10.1099/jmm.0.043000-0. PubMed PMID: 22466031.

41. Pang F, Jia XQ, Song ZZ, Li YH, Wang B, Zhao QG, et al. Characteristics and management of *Enterobacteriaceae* harboring IMP-4 or IMP-8 carbapenemase in a tertiary hospital. Afr Health Sci. 2016;16(1):153-61. Epub 2016/07/01. doi: 10.4314/ahs.v16i1.21. PubMed PMID: 27358627; PubMed Central PMCID: PMCPMC4915421.

42. Reddy K, Bekker A, Whitelaw AC, Esterhuizen TM, Dramowski A. A retrospective analysis of pathogen profile, antimicrobial resistance and mortality in neonatal hospital-acquired bloodstream infections from 2009-2018 at Tygerberg Hospital, South Africa. PLoS One. 2021;16(1):e0245089. Epub 2021/01/15. doi: 10.1371/journal.pone.0245089. PubMed PMID: 33444334; PubMed Central PMCID: PMCPMC7808607.

43. Li P, Wang M, Li X, Hu F, Yang M, Xie Y, et al. ST37 *Klebsiella pneumoniae*: development of carbapenem resistance *in vivo* during antimicrobial therapy in neonates. Future Microbiol. 2017;12:891-904. Epub 2017/07/13. doi: 10.2217/fmb-2016-0165. PubMed PMID: 28699768.

44. Zou H, Shen Y, Li C, Li Q. Two phenotypes of *Klebsiella pneumoniae* ST147 outbreak from neonatal sepsis with a slight increase in virulence. Infect Drug Resist. 2022;15:1-12. Epub 2022/01/14. doi: 10.2147/idr.S343292. PubMed PMID: 35023933; PubMed Central PMCID: PMCPMC8748007.

45. Li J, Hu X, Yang L, Lin Y, Liu Y, Li P, et al. New Delhi metallo-β-Lactamase 1-producing *Klebsiella pneumoniae* ST719 isolated from a neonate in China. Microb Drug Resist. 2020;26(5):492-6. Epub 2019/11/16. doi: 10.1089/mdr.2019.0058. PubMed PMID: 31730396.

46. Yu J, Tan K, Rong Z, Wang Y, Chen Z, Zhu X, et al. Nosocomial outbreak of KPC-2- and NDM-1-producing *Klebsiella pneumoniae* in a neonatal ward: a retrospective study. BMC Infect Dis. 2016;16(1):563. Epub 2016/10/14. doi: 10.1186/s12879-016-1870-y. PubMed PMID: 27733128; PubMed Central PMCID: PMCPMC5062924.

47. Zhang X, Li X, Wang M, Yue H, Li P, Liu Y, et al. Outbreak of NDM-1-producing *Klebsiella pneumoniae* causing neonatal infection in a teaching hospital in mainland China. Antimicrob Agents Chemother. 2015;59(7):4349-51. Epub 2015/05/06. doi: 10.1128/aac.03868-14. PubMed PMID: 25941224; PubMed Central PMCID: PMCPMC4468712.

48. Escobar Pérez JA, Olarte Escobar NM, Castro-Cardozo B, Valderrama Márquez IA, Garzón Aguilar MI, Martinez de la Barrera L, et al. Outbreak of NDM-1-producing *Klebsiella pneumoniae* in a neonatal unit in Colombia. Antimicrob Agents Chemother. 2013;57(4):1957-60. Epub 2013/01/30. doi: 10.1128/aac.01447-12. PubMed PMID: 23357776; PubMed Central PMCID: PMCPMC3623329.

49. Huang X, Cheng X, Sun P, Tang C, Ni F, Liu G. Characteristics of NDM-1-producing *Klebsiella pneumoniae* ST234 and ST1412 isolates spread in a neonatal unit. BMC Microbiol. 2018;18(1):186. Epub 2018/11/16. doi: 10.1186/s12866-018-1334-1. PubMed PMID: 30428842; PubMed Central PMCID: PMCPMC6234558.

50. Gona F, Bongiorno D, Aprile A, Corazza E, Pasqua B, Scuderi MG, et al. Emergence of two novel sequence types (3366 and 3367) NDM-1- and OXA-48-co-producing *K. pneumoniae* in Italy. Eur J Clin Microbiol Infect Dis. 2019;38(9):1687-91. Epub 2019/06/06. doi: 10.1007/s10096-019-03597-w. PubMed PMID: 31165962.

51. Kopotsa K, Mbelle NM, Osei Sekyere J. Epigenomics, genomics, resistome, mobilome, virulome and evolutionary phylogenomics of carbapenem-resistant *Klebsiella pneumoniae* clinical strains. Microb Genom. 2020;6(12). Epub 2020/11/11. doi: 10.1099/mgen.0.000474. PubMed PMID: 33170117; PubMed Central PMCID: PMCPMC8116673.

52. Stoesser N, Giess A, Batty EM, Sheppard AE, Walker AS, Wilson DJ, et al. Genome sequencing of an extended series of NDM-producing *Klebsiella pneumoniae* isolates from neonatal infections in a Nepali hospital characterizes the extent of community- versus hospital-associated transmission in an endemic setting. Antimicrob Agents Chemother. 2014;58(12):7347-57. Epub 2014/10/01. doi: 10.1128/aac.03900-14. PubMed PMID: 25267672; PubMed Central PMCID: PMCPMC4249533.

53. Ramsamy Y, Mlisana KP, Allam M, Amoako DG, Abia ALK, Ismail A, et al. Genomic analysis of carbapenemase-producing extensively drug-resistant *Klebsiella pneumoniae* isolates reveals the horizontal spread of p18-43_01 plasmid encoding *bla*_NDM-1_ in South Africa. Microorganisms. 2020;8(1). Epub 2020/01/23. doi: 10.3390/microorganisms8010137. PubMed PMID: 31963608; PubMed Central PMCID: PMCPMC7023316.

54. Chen CM, Wang M, Li XP, Li PL, Tian JJ, Zhang K, et al. Homology analysis between clinically isolated extraintestinal and enteral *Klebsiella pneumoniae* among neonates. BMC Microbiol. 2021;21(1):25. Epub 2021/01/13. doi: 10.1186/s12866-020-02073-2. PubMed PMID: 33430787; PubMed Central PMCID: PMCPMC7802202.

55. Mukherjee S, Bhattacharjee A, Naha S, Majumdar T, Debbarma SK, Kaur H, et al. Molecular characterization of NDM-1-producing *Klebsiella pneumoniae* ST29, ST347, ST1224, and ST2558 causing sepsis in neonates in a tertiary care hospital of North-East India. Infect Genet Evol. 2019;69:166-75. Epub 2019/01/25. doi: 10.1016/j.meegid.2019.01.024. PubMed PMID: 30677535.

56. Ahmad N, Ali SM, Khan AU. Molecular characterization of novel sequence type of carbapenem-resistant New Delhi metallo-β-lactamase-1-producing *Klebsiella pneumoniae* in the neonatal intensive care unit of an Indian hospital. Int J Antimicrob Agents. 2019;53(4):525-9. Epub 2018/12/24. doi: 10.1016/j.ijantimicag.2018.12.005. PubMed PMID: 30578964.

57. Khajuria A, Praharaj AK, Kumar M, Grover N, Aggarwal A. Multidrug resistant NDM-1 metallo-beta-lactamase producing *Klebsiella pneumoniae* sepsis outbreak in a neonatal intensive care unit in a tertiary care center at central India. Indian J Pathol Microbiol. 2014;57(1):65-8. Epub 2014/04/18. doi: 10.4103/0377-4929.130900. PubMed PMID: 24739834.

58. Jin Y, Shao C, Li J, Fan H, Bai Y, Wang Y. Outbreak of multidrug resistant NDM-1-producing *Klebsiella pneumoniae* from a neonatal unit in Shandong Province, China. PLoS One. 2015;10(3):e0119571. Epub 2015/03/24. doi: 10.1371/journal.pone.0119571. PubMed PMID: 25799421; PubMed Central PMCID: PMCPMC4370709.

59. Zhu J, Sun L, Ding B, Yang Y, Xu X, Liu W, et al. Outbreak of NDM-1-producing *Klebsiella pneumoniae* ST76 and ST37 isolates in neonates. Eur J Clin Microbiol Infect Dis. 2016;35(4):611-8. Epub 2016/01/25. doi: 10.1007/s10096-016-2578-z. PubMed PMID: 26803822.

60. Yu J, Wang Y, Chen Z, Zhu X, Tian L, Li L, et al. Outbreak of nosocomial NDM-1-producing *Klebsiella pneumoniae* ST1419 in a neonatal unit. J Glob Antimicrob Resist. 2017;8:135-9. Epub 2017/01/23. doi: 10.1016/j.jgar.2016.10.014. PubMed PMID: 28109845.

61. Zheng R, Zhang Q, Guo Y, Feng Y, Liu L, Zhang A, et al. Outbreak of plasmid-mediated NDM-1-producing *Klebsiella pneumoniae* ST105 among neonatal patients in Yunnan, China. Ann Clin Microbiol Antimicrob. 2016;15:10. Epub 2016/02/21. doi: 10.1186/s12941-016-0124-6. PubMed PMID: 26896089; PubMed Central PMCID: PMCPMC4761218.

62. Heinz E, Ejaz H, Bartholdson Scott J, Wang N, Gujaran S, Pickard D, et al. Resistance mechanisms and population structure of highly drug resistant *Klebsiella* in Pakistan during the introduction of the carbapenemase NDM-1. Sci Rep. 2019;9(1):2392. Epub 2019/02/23. doi: 10.1038/s41598-019-38943-7. PubMed PMID: 30787414; PubMed Central PMCID: PMCPMC6382945.

63. Khan E, Irfan S, Sultan BA, Nasir A, Hasan R. Dissemination and spread of New Delhi metallo-beta-lactamase-1 superbugs in hospital settings. J Pak Med Assoc. 2016;66(8):999-1004. Epub 2016/08/16. PubMed PMID: 27524536.

64. Kk S, Ekedahl E, Hoang NTB, Sewunet T, Berglund B, Lundberg L, et al. High diversity of *bla*_NDM-1_-encoding plasmids in *Klebsiella pneumoniae* isolated from neonates in a Vietnamese hospital. Int J Antimicrob Agents. 2022;59(2):106496. Epub 2021/12/19. doi: 10.1016/j.ijantimicag.2021.106496. PubMed PMID: 34921976.

65. Farzana R, Jones LS, Rahman MA, Andrey DO, Sands K, Portal E, et al. Outbreak of hypervirulent multidrug-resistant *Klebsiella variicola* causing high mortality in neonates in Bangladesh. Clin Infect Dis. 2019;68(7):1225-7. Epub 2018/09/12. doi: 10.1093/cid/ciy778. PubMed PMID: 30204843.

66. Roy S, Viswanathan R, Singh AK, Das P, Basu S. Sepsis in neonates due to imipenem-resistant *Klebsiella pneumoniae* producing NDM-1 in India. J Antimicrob Chemother. 2011;66(6):1411-3. Epub 2011/03/12. doi: 10.1093/jac/dkr068. PubMed PMID: 21393155.

67. Datta S, Mitra S, Viswanathan R, Saha A, Basu S. Characterization of novel plasmid-mediated β-lactamases (SHV-167 and ACT-16) associated with New Delhi metallo-β-lactamase-1 harbouring isolates from neonates in India. J Med Microbiol. 2014;63(Pt 3):480-2. Epub 2013/12/18. doi: 10.1099/jmm.0.067223-0. PubMed PMID: 24336426.

68. Ahmad N, Khalid S, Ali SM, Khan AU. Occurrence of *bla*_NDM_ variants among *Enterobacteriaceae* from a neonatal intensive care unit in a northern India hospital. Front Microbiol. 2018;9:407. Epub 2018/03/23. doi: 10.3389/fmicb.2018.00407. PubMed PMID: 29563908; PubMed Central PMCID: PMCPMC5845868.

69. Khalid S, Ahmad N, Ali SM, Khan AU. Outbreak of efficiently transferred carbapenem-resistant *bla*_NDM_-producing Gram-negative bacilli isolated from neonatal intensive care unit of an Indian hospital. Microb Drug Resist. 2020;26(3):284-9. Epub 2019/08/10. doi: 10.1089/mdr.2019.0092. PubMed PMID: 31397624.

70. Bhattacharjee B, Bardhan T, Chakraborty M, Basu M. Resistance profiles and resistome mapping of multidrug resistant carbapenem-hydrolyzing *Klebsiella pneumoniae* strains isolated from the nares of preterm neonates. Int J Antimicrob Agents. 2019;53(4):535-7. Epub 2018/12/21. doi: 10.1016/j.ijantimicag.2018.12.002. PubMed PMID: 30572009.

71. Brinkac LM, White R, D'Souza R, Nguyen K, Obaro SK, Fouts DE. Emergence of New Delhi metallo-β-Lactamase (NDM-5) in *Klebsiella quasipneumoniae* from neonates in a Nigerian hospital. mSphere. 2019;4(2). Epub 2019/03/15. doi: 10.1128/mSphere.00685-18. PubMed PMID: 30867330; PubMed Central PMCID: PMCPMC6416368.

72. Kong Z, Cai R, Cheng C, Zhang C, Kang H, Ma P, et al. First reported nosocomial outbreak of NDM-5-producing *Klebsiella pneumoniae* in a neonatal unit in China. Infect Drug Resist. 2019;12:3557-66. Epub 2019/12/10. doi: 10.2147/idr.S218945. PubMed PMID: 31814744; PubMed Central PMCID: PMCPMC6863125.

73. Wei L, Feng Y, Wen H, Ya H, Qiao F, Zong Z. NDM-5-producing carbapenem-resistant *Klebsiella pneumoniae* of sequence type 789 emerged as a threat for neonates: a multicentre, genome-based study. Int J Antimicrob Agents. 2022;59(2):106508. Epub 2021/12/28. doi: 10.1016/j.ijantimicag.2021.106508. PubMed PMID: 34958865.

74. Datta S, Roy S, Chatterjee S, Saha A, Sen B, Pal T, et al. A five-year experience of carbapenem resistance in *Enterobacteriaceae* causing neonatal septicaemia: predominance of NDM-1. PLoS One. 2014;9(11):e112101. Epub 2014/11/19. doi: 10.1371/journal.pone.0112101. PubMed PMID: 25406074; PubMed Central PMCID: PMCPMC4236051.

75. Ballot DE, Bandini R, Nana T, Bosman N, Thomas T, Davies VA, et al. A review of multidrug-resistant *Enterobacteriaceae* in a neonatal unit in Johannesburg, South Africa. BMC Pediatr. 2019;19(1):320. Epub 2019/09/09. doi: 10.1186/s12887-019-1709-y. PubMed PMID: 31493789; PubMed Central PMCID: PMCPMC6731552.

76. Drew RJ, Turton JF, Hill RL, Livermore DM, Woodford N, Paulus S, et al. Emergence of carbapenem-resistant Enterobacteriaceae in a UK paediatric hospital. J Hosp Infect. 2013;84(4):300-4. Epub 2013/07/09. doi: 10.1016/j.jhin.2013.05.003. PubMed PMID: 23831281.

77. Kiaei S, Moradi M, Hosseini-Nave H, Ziasistani M, Kalantar-Neyestanaki D. Endemic dissemination of different sequence types of carbapenem-resistant *Klebsiella pneumoniae* strains harboring *bla*_NDM_ and *16S rRNA* methylase genes in Kerman hospitals, Iran, from 2015 to 2017. Infect Drug Resist. 2019;12:45-54. Epub 2019/01/08. doi: 10.2147/idr.S186994. PubMed PMID: 30613156; PubMed Central PMCID: PMCPMC6306073.

78. Esposito EP, Gaiarsa S, Del Franco M, Crivaro V, Bernardo M, Cuccurullo S, et al. A novel IncA/C1 group conjugative plasmid, encoding VIM-1 metallo-beta-lactamase, mediates the acquisition of carbapenem resistance in ST104 *Klebsiella pneumoniae* Isolates from neonates in the intensive care unit of V. Monaldi Hospital in Naples. Front Microbiol. 2017;8:2135. Epub 2017/11/23. doi: 10.3389/fmicb.2017.02135. PubMed PMID: 29163422; PubMed Central PMCID: PMCPMC5675864.

79. Matilde C, Simone A, Luca G, Fabrizio S. Verona integron-encoded metallo-β-lactamase-producing *Klebsiella pneumoniae* sepsis in an extremely premature infant. Case Reports in Perinatal Medicine. 2018;7.

80. Hassuna NA, AbdelAziz RA, Zakaria A, Abdelhakeem M. Extensively-drug resistant *Klebsiella pneumoniae* recovered from neonatal sepsis cases from a major NICU in Egypt. Front Microbiol. 2020;11:1375. Epub 2020/07/09. doi: 10.3389/fmicb.2020.01375. PubMed PMID: 32636828; PubMed Central PMCID: PMCPMC7317144.

81. Jánvári L, Damjanova I, Lázár A, Rácz K, Kocsis B, Urbán E, et al. Emergence of OXA-162-producing *Klebsiella pneumoniae* in Hungary. Scand J Infect Dis. 2014;46(4):320-4. Epub 2014/02/21. doi: 10.3109/00365548.2013.879993. PubMed PMID: 24552581.

82. Nagaraj G, Shamanna V, Govindan V, Rose S, Sravani D, Akshata KP, et al. High-resolution genomic profiling of carbapenem-resistant *Klebsiella pneumoniae* isolates: a multicentric retrospective Indian study. Clin Infect Dis. 2021;73(Suppl_4):S300-s7. Epub 2021/12/02. doi: 10.1093/cid/ciab767. PubMed PMID: 34850832; PubMed Central PMCID: PMCPMC8634558.

83. Naha S, Sands K, Mukherjee S, Saha B, Dutta S, Basu S. OXA-181-like carbapenemases in *Klebsiella pneumoniae* ST14, ST15, ST23, ST48, and ST231 from septicemic neonates: coexistence with NDM-5, resistome, transmissibility, and genome diversity. mSphere. 2021;6(1). Epub 2021/01/15. doi: 10.1128/mSphere.01156-20. PubMed PMID: 33441403; PubMed Central PMCID: PMCPMC7845606.

84. Labi AK, Nielsen KL, Marvig RL, Bjerrum S, Enweronu-Laryea C, Bennedbæk M, et al. Oxacillinase-181 carbapenemase-producing *Klebsiella pneumoniae* in neonatal intensive care unit, Ghana, 2017-2019. Emerg Infect Dis. 2020;26(9):2235-8. Epub 2020/08/21. doi: 10.3201/eid2609.200562. PubMed PMID: 32818427; PubMed Central PMCID: PMCPMC7454046.

85. Yin D, Dong D, Li K, Zhang L, Liang J, Yang Y, et al. Clonal dissemination of OXA-232 carbapenemase-producing *Klebsiella pneumoniae* in neonates. Antimicrob Agents Chemother. 2017;61(8). Epub 2017/05/24. doi: 10.1128/aac.00385-17. PubMed PMID: 28533245; PubMed Central PMCID: PMCPMC5527636.

86. Mukherjee S, Naha S, Bhadury P, Saha B, Dutta M, Dutta S, et al. Emergence of OXA-232-producing hypervirulent *Klebsiella pneumoniae* ST23 causing neonatal sepsis. J Antimicrob Chemother. 2020;75(7):2004-6. Epub 2020/03/11. doi: 10.1093/jac/dkaa080. PubMed PMID: 32155265.

87. Essel V, Tshabalala K, Ntshoe G, Mphaphuli E, Feller G, Shonhiwa AM, et al. A multisectoral investigation of a neonatal unit outbreak of *Klebsiella pneumoniae* bacteraemia at a regional hospital in Gauteng Province, South Africa. S Afr Med J. 2020;110(8):783-90. Epub 2020/09/04. doi: 10.7196/SAMJ.2020.v110i8.14471. PubMed PMID: 32880307.

88. Berglund B, Hoang NTB, Tärnberg M, Le NK, Welander J, Nilsson M, et al. Colistin- and carbapenem-resistant *Klebsiella pneumoniae* carrying *mcr-1* and *bla*_OXA-48_ isolated at a paediatric hospital in Vietnam. J Antimicrob Chemother. 2018;73(4):1100-2. Epub 2017/12/19. doi: 10.1093/jac/dkx491. PubMed PMID: 29253209.

89. Taoufik L, Amrani Hanchi A, Fatiha B, Nissrine S, Mrabih Rabou MF, Nabila S. Emergence of OXA-48 carbapenemase producing *Klebsiella pneumoniae* in a neonatal intensive care unit in Marrakech, Morocco. Clin Med Insights Pediatr. 2019;13:1179556519834524. Epub 2019/03/23. doi: 10.1177/1179556519834524. PubMed PMID: 30899152; PubMed Central PMCID: PMCPMC6419253.

90. Sharma S, Banerjee T, Kumar A, Yadav G, Basu S. Extensive outbreak of colistin resistant, carbapenemase (*bla*_OXA-48_, *bla*_NDM_) producing *Klebsiella pneumoniae* in a large tertiary care hospital, India. Antimicrob Resist Infect Control. 2022;11(1):1. Epub 2022/01/08. doi: 10.1186/s13756-021-01048-w. PubMed PMID: 34991724; PubMed Central PMCID: PMCPMC8740481.

91. Banerjee T, Wangkheimayum J, Sharma S, Kumar A, Bhattacharjee A. Extensively drug-resistant hypervirulent *Klebsiella pneumoniae* from a series of neonatal sepsis in a tertiary care hospital, India. Front Med (Lausanne). 2021;8:645955. Epub 2021/03/26. doi: 10.3389/fmed.2021.645955. PubMed PMID: 33763435; PubMed Central PMCID: PMCPMC7982647.

92. Ghaith DM, Zafer MM, Said HM, Elanwary S, Elsaban S, Al-Agamy MH, et al. Genetic diversity of carbapenem-resistant *Klebsiella pneumoniae* causing neonatal sepsis in intensive care unit, Cairo, Egypt. Eur J Clin Microbiol Infect Dis. 2020;39(3):583-91. Epub 2019/11/28. doi: 10.1007/s10096-019-03761-2. PubMed PMID: 31773363.

93. Levast M, Poirel L, Carrër A, Deiber M, Decroisette E, Mallaval FO, et al. Transfer of OXA-48-positive carbapenem-resistant *Klebsiella pneumoniae* from Turkey to France. J Antimicrob Chemother. 2011;66(4):944-5. Epub 2011/03/12. doi: 10.1093/jac/dkq504. PubMed PMID: 21393135.

94. Mairi A, Touati A, Ait Bessai S, Boutabtoub Y, Khelifi F, Sotto A, et al. Carbapenemase-producing *Enterobacteriaceae* among pregnant women and newborns in Algeria: Prevalence, molecular characterization, maternal-neonatal transmission, and risk factors for carriage. Am J Infect Control. 2019;47(1):105-8. Epub 2018/09/18. doi: 10.1016/j.ajic.2018.07.009. PubMed PMID: 30220617.

95. Adler A, Solter E, Masarwa S, Miller-Roll T, Abu-Libdeh B, Khammash H, et al. Epidemiological and microbiological characteristics of an outbreak caused by OXA-48-producing *Enterobacteriaceae* in a neonatal intensive care unit in Jerusalem, Israel. J Clin Microbiol. 2013;51(9):2926-30. Epub 2013/06/28. doi: 10.1128/jcm.01049-13. PubMed PMID: 23804390; PubMed Central PMCID: PMCPMC3754643.

96. Dubodelov DV, Lubasovskaya LA, Shubina ES, Mukosey IS, Korostin DO, Kochetkova TO, et al. [Genetic determinants of resistance of hospital-associated strains of *Klebsiella pneumoniae* to β-lactam antibiotics isolated in neonates]. Genetika. 2016;52(9):1097-102. PubMed PMID: 29369564.

97. Singh SK, Gupta M. *bla*_OXA-48_ carrying clonal colistin resistant-carbapenem resistant *Klebsiella pneumoniae* in neonate intensive care unit, India. Microb Pathog. 2016;100:75-7. Epub 2016/10/28. doi: 10.1016/j.micpath.2016.09.009. PubMed PMID: 27622347.

98. Falco A, Ramos Y, Franco E, Guzmán A, Takiff H. A cluster of KPC-2 and VIM-2-producing *Klebsiella pneumoniae* ST833 isolates from the pediatric service of a Venezuelan Hospital. BMC Infect Dis. 2016;16(1):595. Epub 2016/10/25. doi: 10.1186/s12879-016-1927-y. PubMed PMID: 27770796; PubMed Central PMCID: PMCPMC5075218.

99. Battikh H, Harchay C, Dekhili A, Khazar K, Kechrid F, Zribi M, et al. Clonal spread of colistin-resistant *Klebsiella pneumoniae* coproducing KPC and VIM carbapenemases in neonates at a Tunisian university hospital. Microb Drug Resist. 2017;23(4):468-72. Epub 2016/11/02. doi: 10.1089/mdr.2016.0175. PubMed PMID: 27802107.

100. Monaco F, Mento GD, Cuscino N, Conaldi PG, Douradinha B. Infant colonisation with *Escherichia coli* and *Klebsiella pneumoniae* strains co-harbouring *bla*_OXA-48_ and *bla*_NDM-1_ carbapenemases genes: a case report. Int J Antimicrob Agents. 2018;52(1):121-2. Epub 2018/05/13. doi: 10.1016/j.ijantimicag.2018.04.018. PubMed PMID: 29753131.
