# Supplementary material for: Prevalence and clonal diversity of carbapenem-resistant Klebsiella pneumoniae causing neonatal infections: A systematic review of 128 articles across 30 countries
Source: PLoS Med. 2023 Jun 20;20(6):e1004233. doi: 10.1371/journal.pmed.1004233 (PMC10281588; doi:10.1371/journal.pmed.1004233)
Supplement: S8 Table — (DOCX) [file pmed.1004233.s011.docx]

S8 Table. The association of carbapenemases with plasmid replicon types

| Carbapenemase^a^ | Replicon  type | Strain  no.^b^ | ST | Ref | Accession no. |
| --- | --- | --- | --- | --- | --- |
| KPC-nd | FII  (pBK30683) | (0+1) | 147(1) |  | SRR6883068 |
| KPC-2 | FII | 4(4+0) | 147(4) | [1] | SRR6883069 |
| KPC-2 | IncN | 18  (14+4) | 502(10), 140(2), 101(1), 36(1), 17(1), 15(3) | [2] | SRR13246697, SRR13246700, SRR13246701, ERR4795702, GCA_013170405 |
| IMP-1 | IncM2 | 1(1+0) | 915V1(1) | [3] | DRR252233 |
| IMP-4 | IncN | 7(7+0) | 2253(7) | [4] |  |
| IMP-38 | IncHI5 | 13  (13+0) | 307(13) | [5] |  |
| NDM-nd | IncC | 3(0+3) | 15(2), 15V4(1) |  | ERR4920556, ERR5685342, ERR5685449 |
| NDM-1 | IncC | 45  (13+32) | 11(2), 15(26), 15V3(1), 20(1), 22(2), 101(8), 3366(3), 3367(2) | [6] | GCA_021502995, GCA_021503675, ERR4920548, ERR4920554, ERR4920558, ERR4920561, ERR4920564, ERR4920578, ERR4920587, ERR4920590, ERR4920591, ERR5685344, ERR5685345, ERR5685346, ERR5685347, ERR5685362, ERR5685373, ERR5685382, ERR5685386, ERR5685455, ERR5685461, ERR5685465, ERR5685466, ERR5685471, ERR5685474, ERR5685477, ERR5685479, GCA_021502915, ERR5685462, ERR5685464, SRR12149863, SRR12149872 |
| NDM-1 | FI | 21  (18+3) | 14(1), 105(18), 244(1), 2534(1) | [7] | GCA_021503395, GCA_021503755, ERR4920490 |
| NDM-1 | FIIK | 12  (12+0) | 347(5), 29(2), 2558(2), 1224(3) | [8] |  |
| NDM-1 | IncHI1B/  IncFIB | 25  (25+0) | 15(25) | [9] |  |
| NDM-1 | IncX3 | 46  (42+4) | 14(1), 15(5), 37(1), 188(1), 278(23), 719(1), 2735(7), 2736(2), 2738(2), 3350(3) | [10, 11] | SRR19593030, CNA0015420, CNA0015425, SRR16540031, GCA_003429065 |
| NDM-4 | IncC | 1(0+1) | 11(1) |  | GCA_021503115 |
| NDM-5 | FII | 4(4+0) | 14(4) | [12] |  |
| NDM-5 | IncX3 | 61  (44+17) | 11(1), 17(10), 37(1), 334(3), 337(12), 476(5), 789(28), 3003(1) | [13-15] | ERR4920501, SRR17931028, SRR17931029, SRR17931030, SRR17931031, SRR17931032, SRR17931050, SRR17931051, SRR17931052, SRR17931053, SRR17931064, SRR13246761, SRR13246668, SRR13246680, SRR13246726,GCA_002853355, GCA_014655215 |
| NDM-7 | IncC | 2(0+2) | 11(1), 3348V1(1) |  | GCA_021503035, ERR5685335 |
| NDM-7 | IncFIB | 1(0+1) | 711(1) |  | GCA_003368365 |
| NDM-7 | IncX3 | 4(0+4) | 11(1), 22(1), 1998(2) |  | GCA_021502995, ERR5685194, GCA_021503375, ERR5685305 |
| VIM-1 | IncC | 20  (20+0) | 104(20) | [16] |  |
| OXA-48 | FIC | 4(4+0) | 5235(4) | [17] |  |
| OXA-48 | IncHI2 | 2(2+0) | 307(2) | [18] |  |
| OXA-48 | IncL/M | 18  (18+0) | 13(5), 45(1), 1878(1), 101(8), 3366(3), 3367(2) | [6, 19] |  |
| OXA-181 | ColKP3 | 7(7+0) | 48(2), 15(2), 14(3) | [12] | SRR9966444 |
| OXA-181 | IncC | 1(0+1) | 15(1) |  | ERR5685347 |
| OXA-181 | IncX3 | 1(0+1) | 15(1) |  | GCA_021502915 |
| OXA-232 | ColE | 2(2+0) | 15(2) | [20] |  |
| OXA-232 | ColKP3 | 16  (5+11) | 14(12), 23(3), 231(1) | [12, 21] | ERR4920382, ERR4920383, ERR4920386, ERR4920387, ERR5685078, ERR5685080, ERR5685085, ERR5685089, ERR5685090, ERR5685201, GCA_021503755, SRR9966443, SRR9218215, SRR10566676 |

^a^-nd, not assigned

^b^Strain no., those from literature and those from GenBank only (underlined) are shown in parentheses.

^c^IncC also represents IncA/C, IncA/C_1_ and IncA/C_2_ as IncA and IncC are compatible [22].

References

1. Naha S, Sands K, Mukherjee S, Roy C, Rameez MJ, Saha B, et al. KPC-2-producing *Klebsiella pneumoniae* ST147 in a neonatal unit: Clonal isolates with differences in colistin susceptibility attributed to AcrAB-TolC pump. Int J Antimicrob Agents. 2020;55(3):105903. Epub 2020/01/20. doi: 10.1016/j.ijantimicag.2020.105903. PubMed PMID: 31954832.

2. Rada AM, De La Cadena E, Agudelo C, Capataz C, Orozco N, Pallares C, et al. Dynamics of *bla*_KPC-2_ dissemination from non-CG258 *Klebsiella pneumoniae* to other *Enterobacterales* via IncN plasmids in an area of high endemicity. Antimicrob Agents Chemother. 2020;64(12). Epub 2020/09/23. doi: 10.1128/aac.01743-20. PubMed PMID: 32958711; PubMed Central PMCID: PMCPMC7674068.

3. Abe R, Oyama F, Akeda Y, Nozaki M, Hatachi T, Okamoto Y, et al. Hospital-wide outbreaks of carbapenem-resistant *Enterobacteriaceae* horizontally spread through a clonal plasmid harbouring *bla*_IMP-1_ in children's hospitals in Japan. J Antimicrob Chemother. 2021;76(12):3314-7. Epub 2021/09/04. doi: 10.1093/jac/dkab303. PubMed PMID: 34477841.

4. Bai Y, Shao C, Hao Y, Wang Y, Jin Y. Using whole genome sequencing to trace, control and characterize a hospital infection of IMP-4-producing *Klebsiella pneumoniae* ST2253 in a neonatal unit in a tertiary hospital, China. Front Public Health. 2021;9:755252. Epub 2022/01/04. doi: 10.3389/fpubh.2021.755252. PubMed PMID: 34976919; PubMed Central PMCID: PMCPMC8715938.

5. Wang S, Zhao J, Liu N, Yang F, Zhong Y, Gu X, et al. IMP-38-producing high-risk sequence type 307 *Klebsiella pneumoniae* strains from a neonatal unit in China. mSphere. 2020;5(4). Epub 2020/07/03. doi: 10.1128/mSphere.00407-20. PubMed PMID: 32611699; PubMed Central PMCID: PMCPMC7333572.

6. Gona F, Bongiorno D, Aprile A, Corazza E, Pasqua B, Scuderi MG, et al. Emergence of two novel sequence types (3366 and 3367) NDM-1- and OXA-48-co-producing *K. pneumoniae* in Italy. Eur J Clin Microbiol Infect Dis. 2019;38(9):1687-91. Epub 2019/06/06. doi: 10.1007/s10096-019-03597-w. PubMed PMID: 31165962.

7. Zheng R, Zhang Q, Guo Y, Feng Y, Liu L, Zhang A, et al. Outbreak of plasmid-mediated NDM-1-producing *Klebsiella pneumoniae* ST105 among neonatal patients in Yunnan, China. Ann Clin Microbiol Antimicrob. 2016;15:10. Epub 2016/02/21. doi: 10.1186/s12941-016-0124-6. PubMed PMID: 26896089; PubMed Central PMCID: PMCPMC4761218.

8. Mukherjee S, Bhattacharjee A, Naha S, Majumdar T, Debbarma SK, Kaur H, et al. Molecular characterization of NDM-1-producing *Klebsiella pneumoniae* ST29, ST347, ST1224, and ST2558 causing sepsis in neonates in a tertiary care hospital of North-East India. Infect Genet Evol. 2019;69:166-75. Epub 2019/01/25. doi: 10.1016/j.meegid.2019.01.024. PubMed PMID: 30677535.

9. Stoesser N, Giess A, Batty EM, Sheppard AE, Walker AS, Wilson DJ, et al. Genome sequencing of an extended series of NDM-producing *Klebsiella pneumoniae* isolates from neonatal infections in a Nepali hospital characterizes the extent of community- versus hospital-associated transmission in an endemic setting. Antimicrob Agents Chemother. 2014;58(12):7347-57. Epub 2014/10/01. doi: 10.1128/AAC.03900-14. PubMed PMID: 25267672; PubMed Central PMCID: PMCPMC4249533.

10. Yin D, Zhang L, Wang A, He L, Cao Y, Hu F, et al. Clinical and molecular epidemiologic characteristics of carbapenem-resistant *Klebsiella pneumoniae* infection/colonization among neonates in China. J Hosp Infect. 2018;100(1):21-8. Epub 2018/05/16. doi: 10.1016/j.jhin.2018.05.005. PubMed PMID: 29763630.

11. Li J, Hu X, Yang L, Lin Y, Liu Y, Li P, et al. New Delhi metallo-β-Lactamase 1-producing *Klebsiella pneumoniae* ST719 isolated from a neonate in China. Microb Drug Resist. 2020;26(5):492-6. Epub 2019/11/16. doi: 10.1089/mdr.2019.0058. PubMed PMID: 31730396.

12. Naha S, Sands K, Mukherjee S, Saha B, Dutta S, Basu S. OXA-181-like carbapenemases in *Klebsiella pneumoniae* ST14, ST15, ST23, ST48, and ST231 from septicemic neonates: coexistence with NDM-5, resistome, transmissibility, and genome diversity. mSphere. 2021;6(1). Epub 2021/01/15. doi: 10.1128/mSphere.01156-20. PubMed PMID: 33441403; PubMed Central PMCID: PMCPMC7845606.

13. Brinkac LM, White R, D'Souza R, Nguyen K, Obaro SK, Fouts DE. Emergence of New Delhi metallo-β-Lactamase (NDM-5) in *Klebsiella quasipneumoniae* from neonates in a Nigerian hospital. mSphere. 2019;4(2). Epub 2019/03/15. doi: 10.1128/mSphere.00685-18. PubMed PMID: 30867330; PubMed Central PMCID: PMCPMC6416368.

14. Kong Z, Cai R, Cheng C, Zhang C, Kang H, Ma P, et al. First reported nosocomial outbreak of NDM-5-producing *Klebsiella pneumoniae* in a neonatal unit in China. Infect Drug Resist. 2019;12:3557-66. Epub 2019/12/10. doi: 10.2147/idr.S218945. PubMed PMID: 31814744; PubMed Central PMCID: PMCPMC6863125.

15. Wei L, Feng Y, Wen H, Ya H, Qiao F, Zong Z. NDM-5-producing carbapenem-resistant *Klebsiella pneumoniae* of sequence type 789 emerged as a threat for neonates: a multicentre, genome-based study. Int J Antimicrob Agents. 2022;59(2):106508. Epub 2021/12/28. doi: 10.1016/j.ijantimicag.2021.106508. PubMed PMID: 34958865.

16. Esposito EP, Gaiarsa S, Del Franco M, Crivaro V, Bernardo M, Cuccurullo S, et al. A novel IncA/C1 group conjugative plasmid, encoding VIM-1 metallo-beta-lactamase, mediates the acquisition of carbapenem resistance in ST104 *Klebsiella pneumoniae* Isolates from neonates in the intensive care unit of V. Monaldi Hospital in Naples. Front Microbiol. 2017;8:2135. Epub 2017/11/23. doi: 10.3389/fmicb.2017.02135. PubMed PMID: 29163422; PubMed Central PMCID: PMCPMC5675864.

17. Banerjee T, Wangkheimayum J, Sharma S, Kumar A, Bhattacharjee A. Extensively drug-resistant hypervirulent *Klebsiella pneumoniae* from a series of neonatal sepsis in a tertiary care hospital, India. Front Med (Lausanne). 2021;8:645955. Epub 2021/03/26. doi: 10.3389/fmed.2021.645955. PubMed PMID: 33763435; PubMed Central PMCID: PMCPMC7982647.

18. Berglund B, Hoang NTB, Tärnberg M, Le NK, Welander J, Nilsson M, et al. Colistin- and carbapenem-resistant *Klebsiella pneumoniae* carrying *mcr-1* and *bla*_OXA-48_ isolated at a paediatric hospital in Vietnam. J Antimicrob Chemother. 2018;73(4):1100-2. Epub 2017/12/19. doi: 10.1093/jac/dkx491. PubMed PMID: 29253209.

19. Mairi A, Touati A, Ait Bessai S, Boutabtoub Y, Khelifi F, Sotto A, et al. Carbapenemase-producing *Enterobacteriaceae* among pregnant women and newborns in Algeria: Prevalence, molecular characterization, maternal-neonatal transmission, and risk factors for carriage. Am J Infect Control. 2019;47(1):105-8. Epub 2018/09/18. doi: 10.1016/j.ajic.2018.07.009. PubMed PMID: 30220617.

20. Yin D, Dong D, Li K, Zhang L, Liang J, Yang Y, et al. Clonal dissemination of OXA-232 carbapenemase-producing *Klebsiella pneumoniae* in neonates. Antimicrob Agents Chemother. 2017;61(8). Epub 2017/05/24. doi: 10.1128/aac.00385-17. PubMed PMID: 28533245; PubMed Central PMCID: PMCPMC5527636.

21. Mukherjee S, Naha S, Bhadury P, Saha B, Dutta M, Dutta S, et al. Emergence of OXA-232-producing hypervirulent *Klebsiella pneumoniae* ST23 causing neonatal sepsis. J Antimicrob Chemother. 2020;75(7):2004-6. Epub 2020/03/11. doi: 10.1093/jac/dkaa080. PubMed PMID: 32155265.

22. Ambrose SJ, Harmer CJ, Hall RM. Compatibility and entry exclusion of IncA and IncC plasmids revisited: IncA and IncC plasmids are compatible. Plasmid. 2018;96-97:7-12. Epub 2018/02/28. doi: 10.1016/j.plasmid.2018.02.002. PubMed PMID: 29486211.
