# Supplementary material for: Prevalence and clonal diversity of carbapenem-resistant Klebsiella pneumoniae causing neonatal infections: A systematic review of 128 articles across 30 countries
Source: PLoS Med. 2023 Jun 20;20(6):e1004233. doi: 10.1371/journal.pmed.1004233 (PMC10281588; doi:10.1371/journal.pmed.1004233)
Supplement: S9 Table — (DOCX) [file pmed.1004233.s012.docx]

S9 Table. Strain types and virulence factors of the 35 identified possibly hypervirulent neonatal CRKP strains^a^

| Accession no. | Country | ST^b^ | Carbapen-  emase | KL  type^c^ | Virulence factors^d^ | | | | |
| --- | --- | --- | --- | --- | --- | --- | --- | --- | --- |
|  |  |  |  |  | *iuc* | *rmpA* | *rmpA*2 | *iro* | *ybt* |
| ERR4920551 | Pakistan | 15V1 | NDM-1 | nd (112) | *+* | - | - | - | *+* |
| SRR13246721 | Vietnam | 15 | KPC-2 | 10 | *+* | - | *+* | - | - |
| SRR13246686 | Vietnam | 15 | KPC-2 | 10 | *+* | - | *+* | - | - |
| SRR13246681 | Vietnam | 15 | KPC-2 | 10 | *+* | - | *+* | - | - |
| SRR13246670 | Vietnam | 15 | KPC-2 | 10 | *+* | - | *+* | - | - |
| SRR13246638 | Vietnam | 15 | KPC-2 | 10 | *+* | - | *+* | - | - |
| SRR13246636 | Vietnam | 15 | KPC-2 | 10 | *+* | - | *+* | - | - |
| SRR13246629 | Vietnam | 15 | KPC-2 | 10 | *+* | - | *+* | - | - |
| SRR13246624 | Vietnam | 15 | KPC-2 | 10 | *+* | - | *+* | - | - |
| SRR13246620 | Vietnam | 15 | KPC-2 | 10 | *+* | - | *+* | - | - |
| SRR13246615 | Vietnam | 15 | KPC-2 | 10 | *+* | - | *+* | - | - |
| SRR13246612 | Vietnam | 15 | KPC-2 | 10 | *+* | - | *+* | - | - |
| SRR13246611 | Vietnam | 15 | KPC-2 | 10 | *+* | - | *+* | - | - |
| SRR13246606 | Vietnam | 15 | KPC-2 | 10 | *+* | - | *+* | - | - |
| SRR13246605 | Vietnam | 15 | KPC-2 | 10 | *+* | - | *+* | - | - |
| SRR13246603 | Vietnam | 15 | KPC-2 | 10 | *+* | - | *+* | - | - |
| SRR13246740 | Vietnam | 15 | KPC-2 | 10 | *+* | - | *+* | - | - |
| SRR13246716 | Vietnam | 15 | KPC-2 | 10 | *+* | - | *+* | - | - |
| SRR13246714 | Vietnam | 15 | KPC-2 | 10 | *+* | - | *+* | - | - |
| SRR13246713 | Vietnam | 15 | KPC-2 | 10 | *+* | - | *+* | - | - |
| SRR13246705 | Vietnam | 15 | KPC-2 | 10 | *+* | - | *+* | - | - |
| SRR13246694 | Vietnam | 15 | KPC-2 | 10 | *+* | - | *+* | - | - |
| CNA0015900 | China | 11 | KPC-2 | 64 | *+* | *+* | - | - | *+* |
| SRR16539969 | China | 11 | KPC-2 | 64 | *+* | *+* | *+* | - | *+* |
| SRR16539966 | China | 11 | KPC-2 | 64 | *+* | *+* | *+* | - | *+* |
| SRR16539965 | China | 11 | KPC-2 | 64 | *+* | *+* | *+* | - | *+* |
| SRR16539957 | China | 11 | KPC-2 | nd (64) | *+* | *+* | - | - | *+* |
| SRR16539956 | China | 11 | KPC-2 | 64 | *+* | *+* | *+* | - | *+* |
| SRR16539955 | China | 11 | KPC-2 | nd (64) | *+* | *+* | *+* | - | *+* |
| SRR16539950 | China | 11 | KPC-2 | 64 | *+* | *+* | *+* | - | *+* |
| SRR16539945 | China | 11 | KPC-2 | 64 | *+* | *+* | *+* | - | *+* |
| SRR9218215 | India | 23 | OXA-232 | 1 | *+* | *+* | *+* | *+* | *+* |
| SRR10566676 | India | 231 | OXA-232 | 51 | *+* | *+* | - | - | *+* |
| SRR13237632 | India | 15 | NDM-1 | 112 | *+* | *+* | *+* | *+* | *+* |
| SRR13237631 | India | 11 | NDM-1 | 25 | *+* | *+* | *+* | *+* | *+* |

^a^The detailed information of these strains are available in S6 Dataset.

^b^ST, sequence type. 15V1, an unnamed ST with one allele different from ST15.

^c^KL type, capsular type. nd, not determined, with the closest type shown in parentheses.

^d^*iuc* encodes an aerobactin; *rmpA* and *rmpA2* are regulators of mucoid phenotype; *iro* encodes a salmochelin; *ybt* encodes a yersiniabactin. Of note, none of the strains have colibactin-encoding genes.
